# Open Challenges and Opportunities in Piezoelectricity for Tissue Regeneration

**DOI:** 10.1002/advs.202510349

**Published:** 2025-08-18

**Authors:** Xiaoling Deng, Yu Zhuang, Jinjie Cui, Liyun Wang, Huilu Zhan, Xudong Wang, Kaili Lin, Changyong Yuan

**Affiliations:** ^1^ Department of Oral and Maxillofacial Surgery Shanghai Ninth People's Hospital Shanghai Jiao Tong University School of Medicine, College of Stomatology Shanghai Jiao Tong University National Center for Stomatology National Clinical Research Center for Oral Diseases Shanghai Key Laboratory of Stomatology Shanghai Research Institute of Stomatology Shanghai Jiao Tong University School of Medicine Shanghai 200011 China; ^2^ School of Stomatology Xuzhou Medical University Affiliated Stomatological Hospital of Xuzhou Medical University Xuzhou 221004 China

**Keywords:** biomaterials, electrical stimulation, piezoelectric effect, tissue regeneration

## Abstract

Emerging piezoelectric biomaterials have demonstrated their huge potential in diverse medical applications, including ultrasound diagnosis and tissue regeneration. Human body possesses inherent piezoelectricity, producing electrical signal under endogenous load or external pressure to modulate cellular behaviors. Tissue defects caused by traumatic injury will disrupt the electrophysiological microenvironment of the injured area, resulting in unenviable self‐healing. Triggered by physical activities or external stimulation, piezoelectric biomaterials exhibit a unique capability to generate electrical fields to restore the electrophysical microenvironment, reprogram cell fate and ultimately facilitate tissue repair. In this review, endogenous piezoelectric substances in the body and tissue piezoelectricity are introduced. Then, the characteristics and piezoelectricity of piezoelectric biomaterials in regenerative medicine are discussed, as well as strategies to prepare novel piezoelectric composites. Moreover, the molecular mechanisms for the piezoelectric effect on regulating tissue regeneration are systematically summarized. Recent advancements in piezoelectric biomaterials are comprehensively overviewed, including in the regeneration of bone, cartilage, skeletal muscles, tendon, skin, nerve, teeth and periodontal, myocardium, blood vessel and cornea tissues. Finally, the major challenges and future perspectives of piezoelectric biomaterials in regenerative medicine are proposed, hoping to boost the advancement in this promising scientific territory.

## Graphical abstract

This review provides an intriguing review of the recent advancements, challenges, and future perspectives regarding the application of piezoelectric scaffolds in tissue regeneration. The detailed exposition is anticipated to propel the advancement of personalized and intelligent piezoelectric implants, thereby fostering significant progress in this field in the foreseeable future.

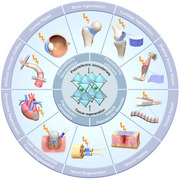

## 1 Introduction

Tissue regeneration involves the use of autologous or allogeneic grafts, stem cell transplantation, or engineered biomaterials to repair damaged tissues and restore their normal function.^[^ ^1^ ^]^ Engineered biomaterials have shown significant potential in filling damaged tissues, restoring cellular activity, and maintaining the physiological microenvironment of the tissue.^[^ ^2^ ^]^ An emerging focus within this field is on piezoelectric biomaterials, which exhibit a notable capability to produce electric signals in response to endogenous load or external pressure. This property makes piezoelectric biomaterials highly adaptable for various medical applications, including ultrasound diagnosis, tumor therapy, and tissue engineering.^[^ ^3^ ^]^ Under mechanical force, piezoelectric biomaterials undergo a piezoelectric reaction, leading to the redistribution of electric charges and the generation of electric dipoles, thereby converting mechanical energy into an electric field.^[^ ^4^ ^]^

Researchers have highlighted that electric fields can modulate cellular activity by altering membrane potential and affecting various signaling pathways.^[^ ^5^ ^]^ The incorporation of piezoelectric materials into biological scaffolds offers a distinct advantage over traditional engineered biomaterials by enabling sustained and targeted electrical stimulation for tissue regeneration, eliminating the reliance on additional growth factors or therapeutic molecules, and thereby mitigating therapeutic limitations and minimizing site‐specific adverse effects.^[^ ^6^ ^]^

In addition, piezoelectricity is a natural property found in various tissues within the body, including bone, articular cartilage, muscles, ligament, skin, and hair.^[^ ^7^ ^]^ These tissues, acting as natural piezoelectric materials, are characterized by a dense arrangement of highly oriented and patterned collagen fibers, making them responsive to mechanical loads.^[^ ^8^ ^]^ When mechanical forces are exerted on these collagen fibrils, they slide relative to one another and undergo deformation, resulting in the generation of a piezoelectric effect.^[^ ^9^ ^]^ As an extracellular matrix (ECM) protein in vertebrate arteries, researchers have also discovered its ferroelectricity ^[^ ^10^ ^]^ at a macroscopic level.^[^ ^11^ ^]^ While it remains an open question whether natural piezoelectricity is related to specific functions, numerous studies have shown its influence on the physical activity of the body.

Piezoelectric biomaterials have the remarkable ability to produce electrical stimulation in response to changes in endogenous load or external pressure.^[^ ^12^ ^]^ This inherent property makes piezoelectric materials highly versatile in tissue engineering.^[^ ^3^, ^13^ ^]^ Moreover, because of this capacity, piezoelectric biomaterials could contribute to restoring the electrophysical microenvironment of tissues.^[^ ^14^ ^]^ Currently, piezoelectric biomaterials employed in tissue engineering can be classified into four primary categories: piezoelectric bioceramics, piezoelectric biopolymers, natural piezoelectric biomaterials, and piezoelectric composites. Piezoelectric bioceramics are polycrystals formed through the sintering of oxides at high temperatures and solid reactions, followed by high‐voltage polarization treatment to induce piezoelectric effects.^[^ ^15^ ^]^ Examples of extensively studied piezoelectric bioceramics for tissue regeneration include zinc oxide (ZnO),^[^ ^16^ ^]^ barium titanate (BaTiO_3_),^[^ ^17^ ^]^ sodium potassium niobate (NaKNbO_3_),^[^ ^18^ ^]^ and Lithium niobate (LiNbO_3_),^[^ ^19^ ^]^ etc. Additionally, piezoelectric polymers, like poly‐L‐lactic acid (PLLA),^[^ ^20^ ^]^ polyvinylidene fluoride (PVDF),^[^ ^21^ ^]^ poly‐β‐hydroxybutyrate (PHB),^[^ ^22^ ^]^ are preferred materials for tissue repair owing to the satisfied biocompatibility and piezoelectric properties.^[^ ^23^ ^]^ Natural piezoelectric biomaterials, such as collagen, cellulose, and silk fibroin, exhibit endogenous piezoelectricity, which can be harnessed in developing biocompatible piezoelectric scaffolds for tissue engineering.^[^ ^24^ ^]^

Understanding the piezoelectric properties, application scenarios, and current limitations of suitable piezoelectric scaffold materials for the restoration of tissue electrophysical microenvironment is imperative. For example, piezoelectric bioceramics are broadly utilized in tissue repair applications because of stable chemical properties, superior mechanical strength, and robust piezoelectric characteristics.^[^ ^25^ ^]^ For instance, BaTiO_3_, as one of the extensively researched piezoelectric bioceramics, exhibits excellent electromechanical coupling ability and minimal dielectric losses, while ZnO demonstrates high electron mobility.^[^ ^26^ ^]^ In contrast to piezoelectric polycrystalline ceramics produced through high‐temperature sintering, the piezoelectric properties of biopolymers can be achieved via various strategies, such as mechanical deformation (e.g., tension, compression) or by applying an external electric field.^[^ ^27^ ^]^ Typical piezoelectric polymers, like PLLA, possess good flexibility and low density, making them particularly suitable for repairing defects in areas subjected to frequent physical movement, such as joints and skin.^[^ ^28^ ^]^

Traditional piezoelectric materials face limitations in the preparation strategy for fabricating personalized tissue piezoelectric scaffold materials, hindering their development and application.^[^ ^29^ ^]^ Injectable piezoelectric composites and intelligent 3D printing bio‐piezoelectric scaffolds, capable of responding to external stimuli, expand the application scenarios of novel piezoelectric biomaterials in tissue regenerative medicine.^[^ ^30^ ^]^ Furthermore, relying solely on electrical fields to modulate cell behaviors often falls short of meeting the requirements for large‐scale defect repair. As a result, researchers have recently explored combining piezoelectric materials with bioactive ions or growth factors, envisioning their coordinated role in tissue engineering.^[^ ^31^ ^]^ While numerous existing reviews focus on the piezoelectric properties and application range of materials, the mechanism by which piezoelectric biomaterials respond to external stimuli and remodel the electrophysical microenvironments at defects has not yet been fully elucidated, not to mention a full spectrum of piezoelectric biomaterials for tissue regeneration.

Understanding how electrical stimulation regulates the behavior of cells in tissue microenvironments is crucial in harnessing piezoelectricity to aid tissue repair and regeneration. For instance, electrical fields have been found to drive bone repair by regulating bone marrow stem cells (BMSCs) to differentiate into osteocytes.^[^ ^32^ ^]^ Under electrical stimulation, the cell membrane potential of BMSCs changes, and ion channels, particularly calcium channels, are activated, allowing extracellular calcium ions to enter the cells.^[^ ^33^ ^]^ These calcium ions, in turn, activate calmodulin within the cells, facilitating the dephosphorylation of nuclear factor of activated T‐cells (NF‐AT) and ultimately regulating stem cell fate.^[^ ^14^, ^34^ ^]^ Furthermore, electrical stimulation also increases Adenosine triphosphate (ATP) production, leading to F‐actin remodeling.^[^ ^35^ ^]^

Interestingly, in the repair progress of cartilage, electrical stimulation induces endogenous transforming growth factor‐β (TGF‐β), leading to chondrification and deposition of extracellular matrix proteins that facilitate cartilage repair.^[^ ^36^ ^]^ However, the underlying mechanisms of this phenomenon remain poorly understood. Apart from bone and cartilage, the microstructure of tendon is intensely hierarchical, and the alignment of collagen fibrils and the direction of the force are consistent.^[^ ^37^ ^]^ A profound understanding of the piezoelectricity and fibers orientation controlled the regenerative progress of tendon tissues has been established by Fernandez‐Yague et al.^[^ ^38^ ^]^, showing that the piezoelectricity of piezoelectric biomaterials have great significance and application prospects in tendon repair. Moreover, the electrical field produced by piezoelectric biomaterials has been proven to possess a therapeutic outcome on osteogenic differentiation of periodontal ligament stem cells (PDLSCs),^[^ ^39^ ^]^ nerve regeneration,^[^ ^40^ ^]^ and chronic wound repair.^[^ ^41^ ^]^ Apart from modulating cell fate, the piezoelectric effect of biomaterials can also promote tissue regeneration by enhancing ECM formation,^[^ ^42^ ^]^ angiogenesis,^[^ ^43^ ^]^ immune microenvironment regulation,^[^ ^44^ ^]^ and bacterial eradication.^[^ ^45^ ^]^ The fifth section of this review will give an elaborate description of the mechanism through which the piezoelectric effect regulates tissue regeneration.

**Figure 1:**
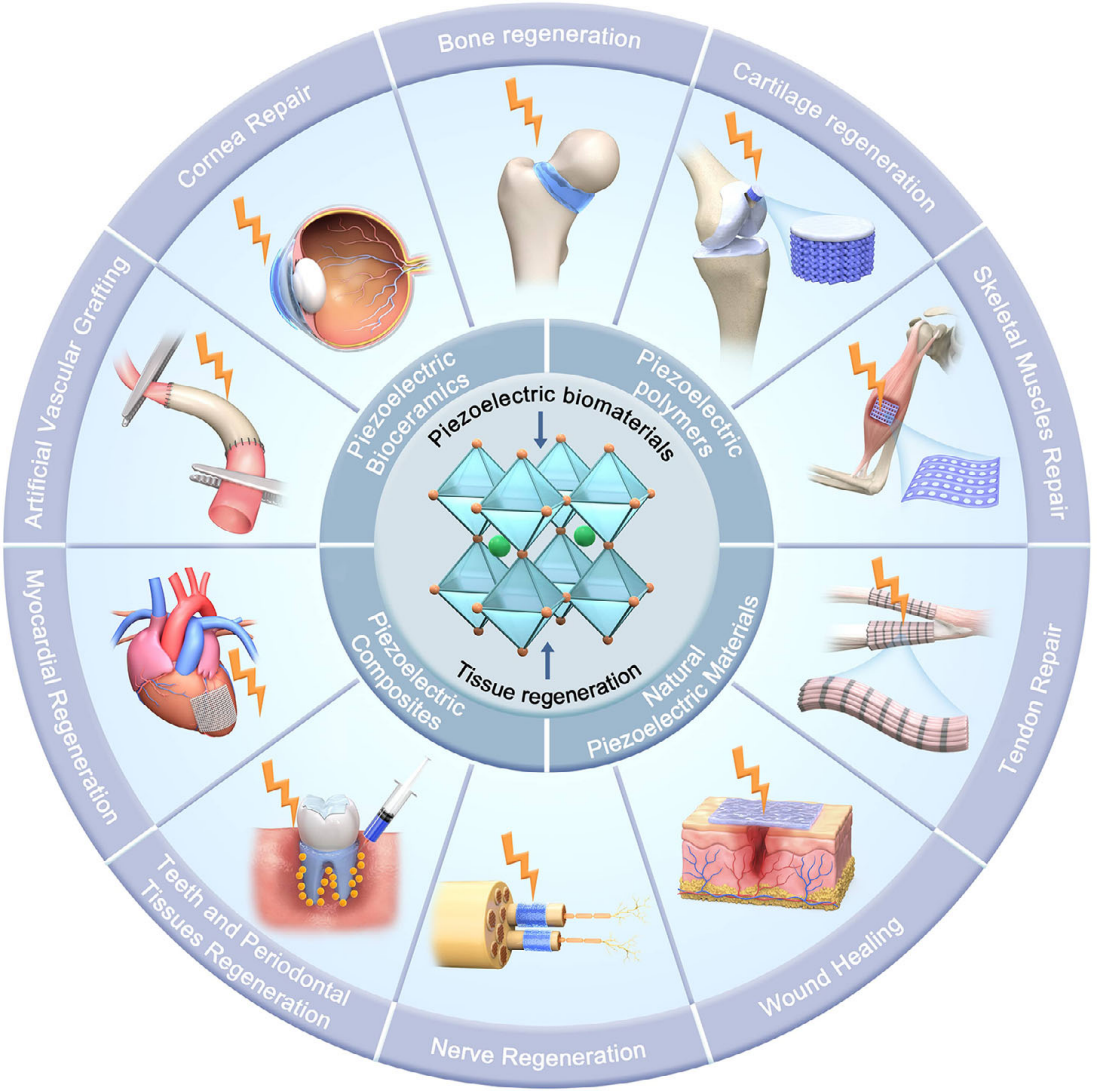
Schematic illustration of piezoelectric biomaterials for tissue regeneration.

This review determines to present a detailed discussion on the synthesis and piezoelectricity of piezoelectric biomaterials, highlighting the pivotal role of endogenous piezoelectricity and electrophysical microenvironments in cellular activity and tissue regrowth. Our work summarizes the major groups of current piezoelectric biomaterials and their distinctive applications in tissue repair, utilizing their responsiveness to external stimuli and regulation of cell fate in tissue microenvironments. (**Figure** **1**) Finally, we discuss the prospect of piezoelectric biomaterials for stimulus response and tissue structure and function adaptation, emphasizing the potential challenges that need to be addressed for clinical translation.

## 2 Endogenous Piezoelectricity

**Figure 2:**
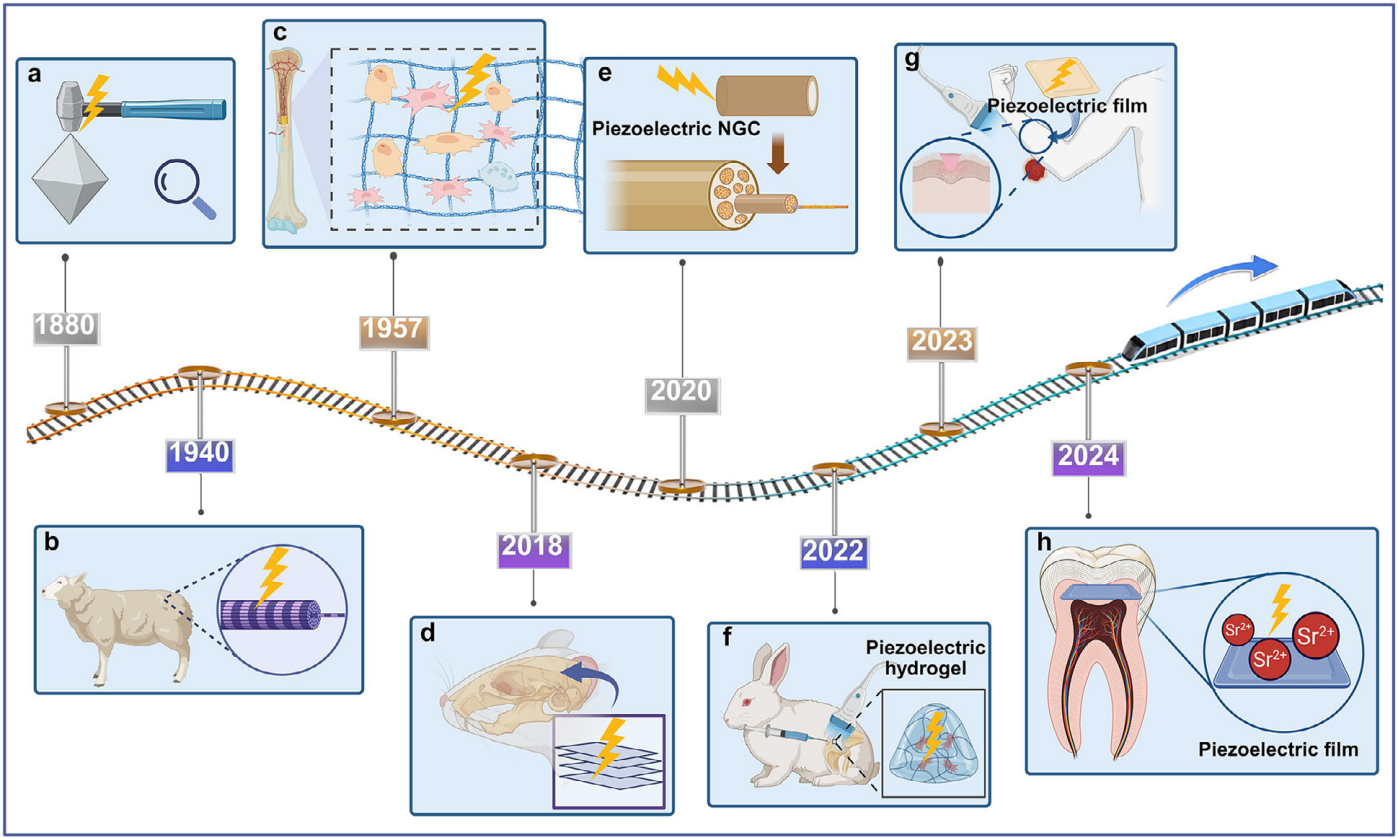
Historical timeline of major developments in piezoelectric biomaterials for regenerative medicine. a) In 1880, Curie brothers first discovered the piezoelectric effect. ^[^ ^46^ ^]^ b) In 1940, the biological piezoelectricity in wool was discovered.^[^ ^48^ ^]^ c) In 1957, Fukada et al. ^[^ ^52^ ^]^ reported the piezoelectricity of bone. d) In 2018, researchers have found that controlling β phases of P(VDF‐TrFE) for bone regeneration. ^[^ ^53^ ^]^ e) In 2020, Piezoelectric materials were applied for nerve repair. ^[^ ^54^ ^]^ f) In 2022, piezoelectric scaffolds were applied for rabbit chondrogenesis. ^[^ ^55^ ^]^ g) In 2023, wearable piezoelectric scaffolds were applied for wound healing. ^[^ ^56^ ^]^ h) In 2024, piezoelectric films were applied for dentin regeneration.^[^ ^57^ ^]^

Since Curie brothers first discovered the mechanical‐electric energy conversion in 1880,^[^ ^46^ ^]^ the fascinating discovery of piezoelectric effect have experienced rapid development in various field. (**Figure** **2**) The relative connection of mechanical variates (strain, S and stress, X) and electrical variates (electric displacement, D and electrical field, E) is manifested as piezoelectricity.^[^ ^47^ ^]^ The piezoelectric coefficient (d) could be described in Equations (1) and (2), where E and X are the independent variates:

(1) $$d=\left(\frac{\partial D}{2a}\right)E$$

(2) $$d=\left(\frac{\partial S}{2E}\right)X$$

Piezoelectric coefficient (d_ij_ = [pC/N]) is commonly used to characterize the piezoelectric properties, containing the shear piezoelectric coefficient (d_15_), longitudinal piezoelectric coefficient (d_33_), and transverse piezoelectric coefficients (d_31_) and (d_24_). In 1940, Martin encapsulated wool in shellac and compressed it between two brass plates, observing its piezoelectric properties.^[^ ^48^ ^]^ During physiological activities, piezoelectricity derived from natural piezoelectric biomacromolecules under mechanical loading participates in the deposition of bone hydroxyapatite (HAp),^[^ ^49^ ^]^ the production of ECM in cartilage,^[^ ^50^ ^]^ the synthesis of tendon proteins,^[^ ^51^ ^]^ and osteogenic differentiation of ligament cells,^[^ ^39^ ^]^ etc. In this part, we provide a systematic review of the discovery history of the piezoelectric effect, as well as the composition and sources of endogenous piezoelectric effects in the body, laying a theoretical foundation for further elucidating the development of piezoelectric biomaterials in tissue regeneration.

### 2.1 Natural Piezoelectric Substances in the Body

**Figure 3:**
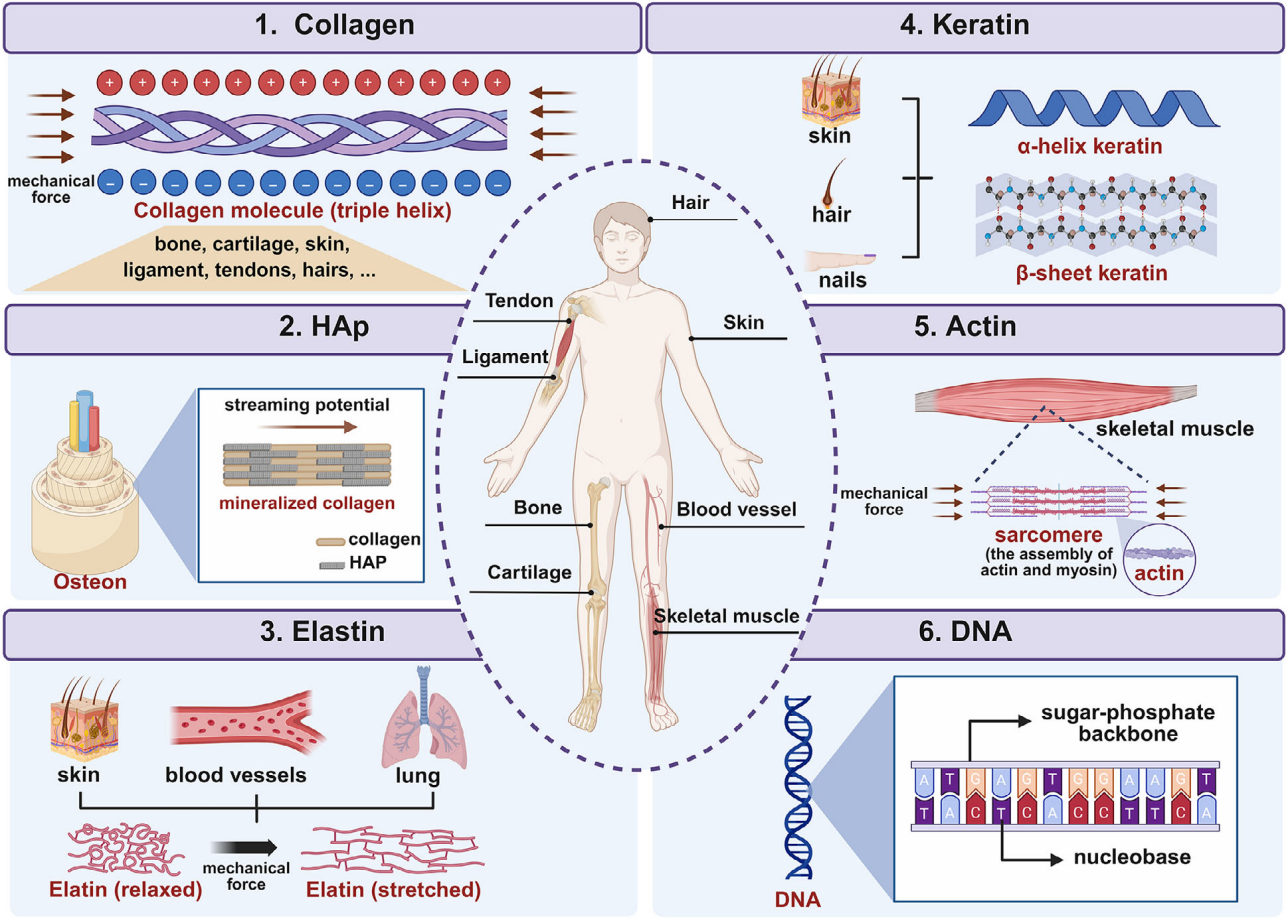
The sources and distribution of endogenous piezoelectricity. (Created with Biorender.com).

Natural piezoelectric substances such as collagen and keratin possess an orchestrated structure with helical or chiral asymmetry characteristics, producing piezoelectricity that regulate cell behaviors and stem cells differentiation, closely related to multiple physiological processes. At the tissue level, the piezoelectricity generated by deformation of ECM in both hard tissue and soft tissue is considered to be connected with tissue growth (**Figure** **3**). The impairment of the natural piezoelectric microenvironment hinders the healing of tissue defect. However, the functionality of piezoelectricity derived from the natural substances and the mechanism of endogenous piezoelectricity in tissue regeneration remains elusive.

#### 2.1.1 Collagen

The triple helix collagen molecule, consisting of three intertwined polypeptide chains, has a molecular weight of ≈300 000.^[^ ^58^ ^]^ It is ≈300 nanometers in length and 1.5 nanometers in diameter. This unique structure imparts significant strength and elasticity to the molecule, highlighting the importance of preserving the integrity of connective tissues such as bones, cartilage, tendons, ligaments, and skin.^[^ ^59^ ^]^ In the 1960s, researchers proposed the concept of collagen piezoelectricity as a possible mechanism by which osteocytes could sense areas of high stress in the bone matrix.^[^ ^9b^ ^]^

As an intrinsic property, the piezoelectricity of collagen fibrils originates from their non‐centrosymmetric structure.^[^ ^60^ ^]^ Specifically, the absence of centrosymmetry in the molecular structure of collagen fibrils enables them to generate electrical charge under mechanical stress, in which the application of external force causes a redistribution of internal charges, leading to a potential difference.^[^ ^61^ ^]^ From an engineering perspective, their piezoelectricity plays a crucial contribution in bone and other connective tissues, as it may influence tissue repair and regeneration processes.^[^ ^62^ ^]^ For example, in bone tissue, pressure‐induced changes in charge distribution may stimulate osteoblast activity, promoting bone formation and repair.^[^ ^63^ ^]^ Thus, the piezoelectric effect of collagen fibrils has important applications in biomaterials, medical engineering, and tissue regeneration. Moreover, the incorporation of collagen into piezoelectric scaffolds holds significant potential for the repair of tissues such as bones, tendons and skin. For instance, collagen is frequently combined with hydroxyapatite to mimic bone matrix, where its inherent piezoelectricity can promote osteoblast activity and mineralization.^[^ ^64^ ^]^ Collagen‐based piezoelectric scaffolds can promote the proliferation, migration and collagen deposition of fibroblasts, thereby facilitating wound healing.^[^ ^56^ ^]^ Additionally, the piezoelectric stimulation generated by collagen in response to mechanical loads can also promote the regeneration of tendon cells and ligament fibroblasts.

#### 2.1.2 Hydroxyapatite (HAp)

Hydroxyapatite (Ca_10_(PO_4_)_6_(OH)_2_, HAp), the major inorganic composition found in bones and teeth, provides rigidity and strength and works in conjunction with the collagen matrix in bone to ensure both resilience and support. Several decades ago, it was widely assumed that HAp had a centrosymmetric crystal structure, implying that it was not a typical piezoelectric molecule and, consequently, could not generate the well‐known piezoelectric effect in bones.^[^ ^65^ ^]^ In 2005, experiments on the stability of the polar phase of HAp proved that it has a monoclinic P2/b structure, and due to the ferroelectric order of the hydroxyl dipoles in its polar phase, HAp may have piezoelectric and pyroelectric properties.^[^ ^66^ ^]^ Bystrov et al.^[^ ^67^ ^]^ employed HyperChem molecular modeling to explore the structure of HAp, revealing the coexistence of its monoclinic and hexagonal phases, particularly in their ordered states along the OH groups. The calculated piezoelectric coefficient d_(yy)_ is approximately equal to d_(33)_, with a value of ≈15.7 pm V^−1^.

#### 2.1.3 Elastin

Elastin is a crucial protein of the extracellular matrix found in all connective tissues of vertebrates, including skin, blood vessels, and lungs, granting elasticity to tissues that repeatedly endure physiological stress.^[^ ^68^ ^]^ As early as the 1960s, Fukada et al.^[^ ^69^ ^]^ observed the piezoelectricity of blood vessel walls, demonstrating that a 1 Hz cyclic deformation in the blood vessel walls generates an alternating current of ≈3 picoamperes across the surfaces of the blood vessel walls. With advances in histology and piezoresponse force microscopy, researchers have elucidated the hierarchical microstructure and ferroelectric properties of elastin.^[^ ^11^ ^]^ The microstructure of elastin is composed of amorphous elastin fibers formed by the cross‐linking and assembly of tropoelastin, which are surrounded by microfibrils to form an extensive, stable, and elastic network, enabling tissues to return to their original shape after deformation while preserving their intrinsic functions. Notably, in tissues such as arteries and lungs, the durability of elastin enables it to retain functionality under continuous mechanical stress for several decades (with a half‐life of ≈70 years), withstanding over a billion cycles of stretching.^[^ ^70^ ^]^ It is equally remarkable that elastin could keep its ferroelectricity at 473K, with polarization stemming from cooperative dipole rotation driven by thermal activation.

The origin of elastin's piezoelectricity involves both its microstructure and molecular composition, along with its capacity to generate charges under mechanical stress. First of all, elastin contains polar groups within its amino acid sequence, which rearrange when exposed to mechanical stress, such as stretching or compression, leading to the separation and accumulation of charges. The interaction among these polar groups induces elastin's piezoelectric properties.^[^ ^68^, ^71^ ^]^ The cross‐linking and arrangement of elastin fibers further contributes to their piezoelectric properties. Lysine cross‐linking within elastin holds protein molecules together through covalent bonds, and this cross‐linking network induces microstructural displacement under mechanical deformation, resulting in an alter in the electric dipole moment, which generates an electric charge.^[^ ^72^ ^]^ This molecular‐level cross‐linked structure plays a crucial role in elastin's piezoelectric effect. Moreover, the interaction between elastin fibers and other components, such as collagen and microfibrils, along with ions present in the biological environment, may further amplify their piezoelectric effect.^[^ ^73^ ^]^ However, recent studies indicate that the piezoelectricity of elastin may be suppressed by glucose during aging and disease, making the switching process significantly more difficult.^[^ ^74^ ^]^

#### 2.1.4 Keratin

Keratin is a structural protein with piezoelectric properties that can respond to mechanical forces by creating weak electrical fields. It is mainly found in keratinized tissues such as skin, hair, and nails.^[^ ^75^ ^]^ The piezoelectric effect of keratin helps tissues, such as the skin, sense external physical stimuli like pressure and stretching, and it may contribute to biosensing and wound repair.^[^ ^76^ ^]^ Its piezoelectric properties are mainly influenced by its molecular structure and arrangement.^[^ ^77^ ^]^ From a molecular perspective, the amino acid sequence of keratin and its folding pattern impart specific electrical properties. Its secondary structure mainly consists of α‐helical and β‐sheet structures, which can undergo slight displacements when subjected to stress, leading to alterations in charge distribution. Polar groups, such as polar amino acids like tyrosine and glutamic acid, can generate an electromotive force when subjected to stress, thereby conferring piezoelectric properties to keratin.^[^ ^78^ ^]^ At the same time, the orientation of keratin fibers influences their piezoelectric properties to some degree. For example, when mechanical pressure is applied along this alignment, it generates electric charge more efficiently.

#### 2.1.5 Actin

Regarding the piezoelectric properties of actin, there is still relatively unexplored within the academic community. Although actin plays an important structural and functional role in the cytoskeleton, its piezoelectric properties have not been subjected to the same level of extensive research and detailed characterization as other proteins, such as collagen or keratin. Early research on actin primarily centered on its biomechanical and structural roles, whereas its piezoelectric properties have garnered increased attention recently with the advancement of biomaterials and nanotechnology. Specifically, scientists have started investigating the electrical responses of many biomolecules under stress, including actin, as these molecules frequently undergo mechanical stimuli such as stretching and compression in a biomechanical environment.

#### 2.1.6 Deoxyribonucleic Acid (DNA)

During the past decades, piezoelectric effect has been found in various biopolymers.^[^ ^79^ ^]^ DNA, composed of a double‐helix structure formed by four bases, is the essential genetic material required for the development and normal functioning of all living organisms.^[^ ^80^ ^]^ Dating back to the 1960s, Duchesne et al.^[^ ^81^ ^]^ have indicated the piezoelectric properties of DNA, and the piezoelectric strain constant of DNA‐oriented films has been determined across a temperature spectrum of −170 to 9 °C. Because of the non‐centrosymmetric nature of DNA's helical structure, mechanical forces (such as stretching or bending) may cause a redistribution of charge, thereby inducing a piezoelectric effect.^[^ ^82^ ^]^

Additionally, intermolecular interactions within DNA, such as hydrogen bonds between base pairs and covalent bonds within the phosphate backbone, may lead to subtle charge displacement under mechanical stress, thereby imparting piezoelectric properties. Studies have shown that DNA molecules may exhibit more pronounced piezoelectricity when oriented in a specific direction. Specifically, when DNA molecules are aligned in a certain manner, the charge distribution under mechanical force may become more ordered, thereby amplifying the piezoelectric effect. Although scientists have identified the piezoelectric effect of DNA and its potential origins, its piezoelectricity remains a field that is not yet fully understood. Future research may further clarify the specific mechanisms behind DNA's piezoelectric properties and explore its potential applications.

### 2.2 Endogenous Piezoelectricity in Biological Tissues

Many tissues in the body display piezoelectric properties, particularly those containing specific structural proteins like collagen and keratin. When exposed to mechanical stress, these tissues undergo changes in electric charge. For example, bone is a piezoelectric composite, containing 69 wt% HAp nanocrystals and 22 wt% collagen.^[^ ^83^ ^]^ First reported by Fukada and Yasuda in 1957,^[^ ^52^ ^]^ piezoelectricity of bone is mainly attributed to collagen fibers, impregnated by HAp nanocrystals.^[^ ^84^ ^]^ It has been proven that the slipping between collagen fibers results in the piezoelectricity of natural bone.^[^ ^85^ ^]^ The piezoelectric strain coefficients of human tibia were monitored by Halperin et al.^[^ ^62a^ ^]^, ranging from 7.7 to 8.7 pC N^−1^ without sizable scatter. The piezoelectric reply of bone is anisotropic due to its anisotropic structure. The highest piezoelectric efficiency of femur could be reached at 0.7 pC N^−1^ under the shear mode.^[^ ^86^ ^]^ Nevertheless, because of the existence of streaming potential and shear force generated by fluid, the sole relevance between piezoelectricity and bone remodeling is still unclear to a certain extent.^[^ ^87^ ^]^ That is, the electrical field generated from the deformation of bone under mechanical mainly results from the piezoelectricity of bone and streaming potential of fluid.^[^ ^88^ ^]^ A consensus among researchers is that the electrical potential is dominantly evoked by inherent piezoelectricity of dry bone, as for the wet one, the ion flow in interstitial fluid functions a lot in generating electrical potential.^[^ ^7^, ^89^ ^]^

As a ferroelectric phenomenon, piezoelectricity couples the mechanical energy with electrical energy. When a nonsymmetric structure subjected to mechanical stress, the inner polar groups would redirect along the applied strain, generating a dipole about the axis.^[^ ^62^, ^90^ ^]^ Cartilage tissues, including articular cartilage and intervertebral disc, possess piezoelectricity that is caused by the deformation of collagen under mechanical stress. Due to the existence of natural fluid‐driven streaming potential, it is difficult to measure the separated piezoelectricity of cartilaginous tissues. In 2020, Poillot et al.^[^ ^91^ ^]^ have attempted to monitor the piezoelectricity of intervertebral disc via using piezoresponse force microscopy (PFM) to measure the electrical change which was directly induced by mechanical stress. The longitudinal piezoelectricity of the intervertebral disc has been investigated to ≈1 pC N^−1^, while the piezoelectric effect of Annulus Fibrosus was higher than that in Nucleus Pulposus because of the organized collagen networks in Annulus Fibrosus. As for articular cartilage, 80% of it is made up of water, and the rest is mostly collagen fibers and proteoglycans, and there are a small number of chondrocytes between collagen fibers and proteoglycans.^[^ ^92^ ^]^ The extracellular matrix of cartilage is composed of collagen, proteoglycan, various proteins, and tissue fluid. Among them, type II collagen could resist the mechanical tension while the corresponding piezoelectric materials possess a piezoelectric coefficient (d_33_) of 0.7 pC N^−1^.^[^ ^93^ ^]^

Tendons, as a resilient bridge of connective tissue, serve to connect muscles to bones and to resist tension. Like ligaments and fascia, tendons consist of a highly parallel and taut collagen structure, which primarily consists of 97–98% type I collagen alongside small amounts of other collagen types. The hierarchical collagen fibers could generate electrical field and polarization current under mechanical deformation, resulting in a piezoelectrical phenomenon in tendons.^[^ ^94^ ^]^ The piezoelectricity of tendons was first measured by Marino et al.^[^ ^95^ ^]^ in 1975, in form of dry dehydrated, and frozen hydrated conditions. Recent study has demonstrated that the piezoelectricity of tendon is related to the room humidity, a low humidity (10% relative humidity) would lead to a decreased piezoelectric response of a rat tail tendon.^[^ ^96^ ^]^ This result suggested that the relative humidity of tendon might affect the deformability and electromechanical coupling ability of collagen fibers.

Both tendons and ligaments share a parallel arrangement of collagen fibers. In vitro experiments have found that very oriented collagen in the ECM can sustain the phenotype of ligament cells.^[^ ^97^ ^]^ However, detailed research is necessary to figure out the piezoelectric effect and flow potential of ligaments under mechanical stress. Contrastingly, the collagen fibers in the skin form a reticular structure, providing it with omnidirectional stretchability. When subjected to external pressure, the skin exhibits pyroelectric and piezoelectric properties, generating electrical potential.^[^ ^98^ ^]^ In 1986, Rossi et al.^[^ ^99^ ^]^ conducted measurements to determine the piezoelectric origin of each skin layer, specifically the dermis, epidermis, and stratum corneum. Research has shown that collagen serves as the source of piezoelectricity in the dermis, while keratin serves as the source of piezoelectricity in the epidermis and stratum corneum. The maximum piezoelectric constants for the dermis, epidermis, and stratum corneum are determined to be 0.1, 0.03, and 0.2 pC N^−1^, respectively.^[^ ^59^ ^]^

In 1969, Fukada and coworkers first discovered the piezoelectric effect of blood vessels, measuring an electric current of ≈3 picoampere produced on the surface of the blood vessel wall during deformation by one tilt cycle at a frequency of ≈1 cycle per second.^[^ ^69^ ^]^ Subsequently, in 2012, Liu et al.^[^ ^100^ ^]^ quantified the piezoelectric coefficient of porcine aortic wall to be ≈1 pm V^−1^ via the help of PFM. However, due to the complex composition of the aorta, considering the intima containing longitudinal collagen fibers, an elastic membrane arranged in concentric circles, and an adventitia consisting of longitudinally spiral collagen fibers and elastic fibers, it remains challenging to determine if the entire structure of porcine aortic walls is ferroelectric. In 2017, Lenz et al.^[^ ^73a^ ^]^ conducted tests on whole porcine aortic walls, suggesting that the aortic walls acted as a dielectric material, yet macroscopic measurements failed to definitively ascertain whether the electromechanical response stems from the piezoelectricity of aortic walls rather than Maxwell force and electrostriction. Recently, researchers detected the piezoelectric response in the intima of mouse arteries using PFM, indicating that the intimal layer of the mouse arteries was predominantly linear piezoelectric.^[^ ^101^ ^]^

## 3 Piezoelectric Biomaterials for Regenerative Medicine

**Figure 4:**
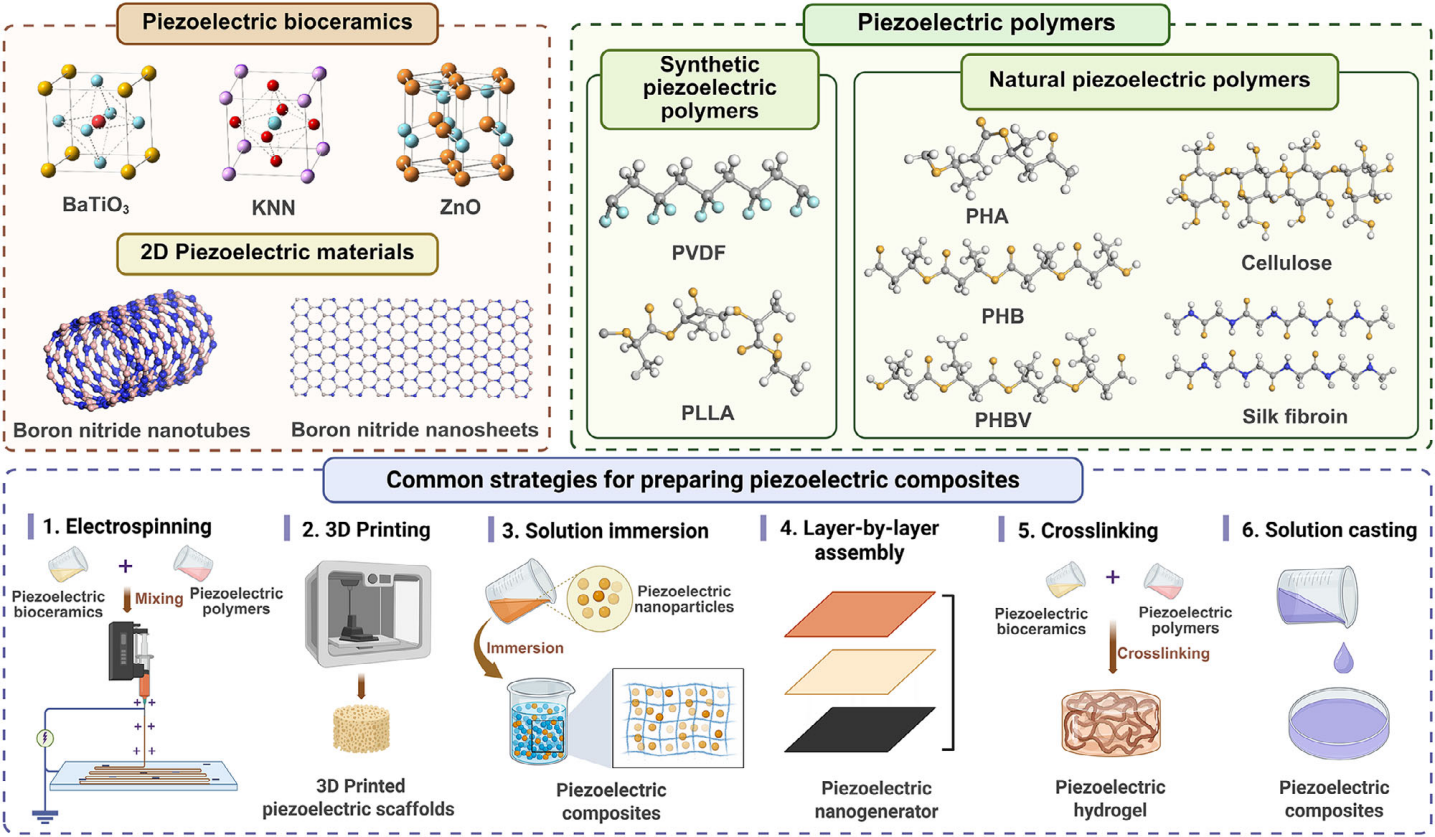
The schematic diagram illustrates the piezoelectric biomaterials used in regenerative medicine and common strategies for preparing piezoelectric composites. (Created with Biorender.com).

Piezoelectric scaffolds, which could generate electrical stimulation under mechanical force or other stimulations, are widely used for the repair of bone,^[^ ^102^ ^]^ cartilage,^[^ ^103^ ^]^ skeletal muscles,^[^ ^104^ ^]^ tendons,^[^ ^105^ ^]^ ligament,^[^ ^106^ ^]^ nerve,^[^ ^107^ ^]^ tooth,^[^ ^108^ ^]^ wound healing,^[^ ^109^ ^]^ and so on. Specifically, piezoelectric effect is observed in some dielectric materials, where polarization occurs when the material is subjected to an external force. Once the external force is removed, the material will shift from the charged state to the original uncharged state.^[^ ^110^ ^]^ In 1880, the French physicists first observed this piezoelectric phenomenon while studying quartz crystals.^[^ ^111^ ^]^ While how the piezoelectric effect at work in bone repair was first presented in 1969 by Peccatori and colleagues.^[^ ^112^ ^]^ Since their introduction into regenerative medicine, piezoelectric materials have evolved from structured ceramics to include various categories, such as piezoelectric bioceramics, piezoelectric biopolymers, and piezoelectric composites (**Figure** **4**). This section offers an overview of piezoelectric materials in tissue regeneration, aiming to provide useful insights for the design of innovative and individualized piezoelectric scaffolds (**Table** **1**).

**Table 1:** Integration about piezoelectricity and application scenarios of piezoelectric materials.

Type	Representative materials	Piezoelectricity	Preparation method	Application scenarios	Refs.
Piezoelectric bioceramics	Ca/Mn‐doped BaTiO_3_ nanofibers	d_33_ = 0.9–3.7 pC N^−1^	Sol–gel system	Bone regeneration	[126]
	BaTiO_3_/OHA hydrogel	N/C	Hydrothermal method	Infected wound healing	[216]
	BaTiO_3_@MMSa	N/C	Membrane coating	Infected wound healing	[217]
	BaTiO_3_ hydrogel	Output voltage ≈10 mV mm^−2^	Continuous mixture	Periodontal disease treatment	[218]
	Tetragonal BaTiO_3_ hydrogel	Piezopotential = 45.4 mV	Thermal calcination	Periodontal disease treatment	[39]
	P‐KNN/BG	Surface potential = 322.04 ± 12.85 mV	Solid‐state synthesis	Angiogenesis	[31]
	PCL/ZnO nanofiber	N/C	Electrospinning	Nerve regeneration	[137b]
	PCL/ZnO scaffolds	N/C	Electrospinning	Burn injuries repair	[219]
	Boron nitride nanosheets (BNNS)@PCL	d_33_ = 3.3–25.7 pC N^−1^	layer‐by‐layer droplet spraying	Nerve repair	[147]
Piezoelectric polymers	PVDF/PCL	d_33_ = 13.2 p.m. V^−1^	Cast/annealing‐solvent displacement	Nerve repair	[162]
	SF/PEDOT‐PVDF/PLCL	N/C	Gradient freezing	Nerve repair	[54]
	PVDF/ZIF‐8 foam	d_33_ = 3.5 pC N^−1^	Solid‐state shearing milling	Bone repair	[164b]
	TiO_2_@PVDF	d_33_ = 20.81 p.m. V^−1^	Electrospinning	Bone repair	[167]
	PPy/ PDA/PLLA	N/C	Electrospinning	Nerve regeneration	[174]
	CNTs/GelMA/PLLA	Output voltage = 5.96 V	Electrospinning	Nerve regeneration	[173]
	P(VDF‐TrFE)	d_33_ = −24.61pm V^−1^	Electrospinning	Tendon repair	[38]
	PLLA	N/C	Electrospinning	Wound healing	[56]
	P(VDF‐TrFE)	Output voltage = 6278 ± 954.92 mV	Electrospinning	Wound healing	[220]
	SrCl_2_/ P(VDF‐TrFE)	d_33_ = 14 pC N^−1^	Dry process and Annealing	Dentin tissue regeneration	[57]
	VMLRE	Output voltage = 0.15–4.7 V	Copolymerization	Muscle tissue repair	[189]
	PPBE	Output voltage = 0.06 to 0.43 V	Copolymerization	Muscle tissue repair	[104]
	PETRR	Output voltage = 0.149 to 0.43 V	Copolymerization	Tendon regeneration	[190]
Natural piezoelectric polymers	CaCO_3_‐mineralized PHBV	d_33_ = 0.7–1.0 pC N^−1^	Electrospinning	Bone engineering	[183]
	Cellulose acetate (CA) fibers	d_31_ = 6.68 ± 1.70 pmV^−1^	Electrospinning	Bone regeneration	[196]
Piezoelectric Composites	PLLA/BaTiO_3_‐GO	The piezoelectric current reached to 147.3nA	Selective laser sintering	Bone regeneration	[127b]
	PLA/KNN@PDA	d_33_ = 20 pC N^−1^	Electrospinning	Nerve regeneration	[135]
	PVDF/ZnO/SA hydrogel	N/C	3D printing	Wound healing	[141]
	PLLA/CMBT	d_33_ = 1.92 pC N^−1^	Sol‐gel electrospinning and calcination	Bone regeneration	[171]
	PHBV/PLLA/KNN	d_33_ = 1.59 pC N^−1^	Spin coating	Nerve regeneration	[221]
	SF/PVDF‐HFP/MXene	Output voltage = 100 mV	Electrospinning	Nerve regeneration	[204]
	PLLA‐collagen scaffold	Output voltage = 3.6 V	Layer‐by‐layer assembly	Cartilage regeneration	[55]
	NF‐sPLLA collagen hydrogel	Output voltage ≈33.7 mV	Electrospinning	Cartilage regeneration	[222]
	PHBV/BaTiO_3_	d_33_ = 1.4 ± 0.03 pC N^−1^	Electrospinning	Cartilage regeneration	[14a]
	PHBV/PLLA	N/C	3D printing	Cartilage regeneration	[223]
	dECM/Gel‐PC	N/C	Freeze‐drying and compression	Osteochondral defects repair	[224]
	PLLA/ZnO and PLLA/BaTiO_3_	Output voltage = 2.5 V	Electrospinning	Tendon‐to‐bone healing	[225]
	Patterned PVDF/BaTiO_3_	Output voltage = 21.6 V	Electrospinning	Nerve regeneration	[226]
	BTNPs/P(VDF‐TrFE)	Output voltage = 1.69 V	Electrospinning	Peripheral nerve regeneration	[227]
	Smart patch (PRG‐G‐C)	Output voltage = ±35 mV	Electrospinning and crosslinking	Skin nerve regeneration	[228]
	Au@BaTiO_3_	Output voltage = 0.59 V	In situ bottom–up growth	Spinal cord injury repair	[229]
	TiO_2_/ BaTiO_3_/Au	N/C	In situ reaction	Infected wound healing	[230]
	PVDF/CPDA hydrogel	Output voltage ≈1.0 V	Electrospinning	Wound healing	[231]
	PVA/PVDF	Output voltage = 3V	Freezing/thawing‐solvent replacement‐annealing‐swelling	Diabetic wound repair	[232]
	CoFe_2_O_4_@CTAB/PVDF	Output voltage = 0.5 V	Electrospinning	Chronic wound healing	[41]
	PVDF/PCL/PCL‐Fe_3_O_4_	N/C	Multimaterial 3D printing	Cardiac regeneration	[233]
	PCL/PVDF/AgNW/PCL	Piezopotential ≈0.4 V	Electrospinning	Vascular graft	[234]
	pHEMA/PDMS/PEG/ITO	d_33_∼145 pC N^−1^	Femtosecond laser patterning	Corneal injury repair	[235]

### 3.1 Piezoelectric Bioceramics

In several non‐centrosymmetric crystal classes, spontaneous polarization occurs in response to temperature variations, and its magnitude can be altered through externally applied electrical fields.^[^ ^113^ ^]^ This inherent property of crystals is termed ferroelectricity, and materials possessing ferroelectricity are referred to as ferroelectrics.^[^ ^110^ ^]^ Specifically, ferroelectric ceramics that exhibit piezoelectric effects post‐polarization are categorized as piezoelectric ceramics.^[^ ^89^ ^]^ Among the array of piezoelectric bioceramics, potential candidates for tissue regeneration include barium titanate (BaTiO_3_), (K, Na) NbO_3_ (KNN) or KNN‐based ceramics, and Zinc oxide (ZnO). These materials highlighted the advantages of low cost, satisfied biocompatibility, and chemical stability.

#### 3.1.1 Barium Titanate (BaTiO_3_)

Barium titanate (BaTiO_3_), with BaTiO_3_ or its solid solution as the primary crystal phase, represents a prototypical ferroelectric material featuring an ABO_3_ perovskite structure.^[^ ^114^ ^]^ The piezoelectricity of BaTiO_3_ ceramics was first found in the 1940s, initiating over 80 years of development in piezoelectric BaTiO_3_ ceramics.^[^ ^115^ ^]^ BaTiO_3_ is a wide bandgap ferroelectric semiconductor characterized by predominantly cubic and tetragonal crystalline phases. Notably, the tetragonal phase of BaTiO_3_ stands out as a typical piezoelectric material, exhibiting polarization under pressure, leading to the separation of internal electrons and holes, ultimately generating voltage.^[^ ^116^ ^]^ BaTiO_3_ emerges as a promising piezoelectric scaffold in tissue regeneration, demonstrating good in vivo biocompatibility and distinctive nonlinear optical properties.^[^ ^117^ ^]^ Nevertheless, the utilization of BaTiO_3_ in tissue regeneration faces multiple limitations because of its suboptimal biological activity and osteoconductivity.^[^ ^118^ ^]^

The piezoelectric strain coefficient, denoted as the relationship of strain and the electric field, is typically expressed as the piezoelectric strain coefficient “d”.^[^ ^119^ ^]^ The crystal structure and orientation of BaTiO_3_ result in directional variations in its piezoelectric strain coefficient.^[^ ^120^ ^]^ The primary methods for preparing BaTiO_3_ ceramics include: 1. Pre‐synthesis of BaCO_3_ and TiO_2_, followed by sintering at 1280–1400 °C; 2. Chemical preparation of high‐purity ultra‐fine BaTiO_3_ powder, followed by direct firing after molding.^[^ ^121^ ^]^ However, the piezoelectric constant d_3__3_ value of BaTiO_3_ ceramics prepared using these methods is ≈190 pC N^−1^, limiting its application in electronic sensing and regenerative medicine.^[^ ^122^ ^]^ Various factors influence the d_33_ of BaTiO_3_ ceramics, including porosity,^[^ ^123^ ^]^ sintering temperature and atmosphere,^[^ ^124^ ^]^ grain size,^[^ ^125^ ^]^ among others.

As previously mentioned, BaTiO_3_ ceramics have demonstrated their potential in regenerative medicine because of their high piezoelectric properties. However, they are not often employed independently due to the absence of bioactive ions essential for promoting tissue regeneration, particularly in bone regeneration. To make them more suitable for cell culture and implantation in the body, ion‐doping stands out as a commonly employed strategy to improve the biological activity of BaTiO_3_ ceramics. Calcium ions, fundamental components of bones, play an important role in regulating the growth and differentiation of BMSCs, contributing significantly to bone formation and fracture healing. In 2021, Zheng and colleagues successfully doped Ca^2^⁺ (10 mol%) and Mn⁴⁺ (2 mol%) into BaTiO_3_ nanofibers, achieving enhanced osteogenic differentiation of BMSCs without inducing cytotoxicity.^[^ ^126^ ^]^ Another critical limitation hindering the application of BaTiO_3_ in bone regeneration is the presence of ubiquitous oxygen vacancies, resulting in lattice defects and interfering the arrangement of polarized charges. To address this issue and impairing the commencement of oxygen vacancies, graphene oxide (GO) was introduced, allowing for the in situ development of BaTiO_3_.^[^ ^127^ ^]^ The C═O and C─OH groups of GO were cleaved and dehydrogenated under high‐temperature treatment, producing negatively charged oxygen groups that occupied the oxygen vacancies of BaTiO_3_.^[^ ^128^ ^]^ Additionally, reduced GO could serve as a conductive phase to promote the polarized charge transfer of BaTiO_3_. It has been demonstrated that the introduction of GO has enhanced the piezoelectric current of materials and accelerated osteogenic differentiation in vitro.

#### 3.1.2 Potassium Sodium Niobate (KNN) and KNN‐Based Ceramics

Potassium sodium niobate (KNN) ceramics are anticipated to have prevalent applications in tissue regeneration owing to substantial piezoelectric constant and high Curie temperature.^[^ ^129^ ^]^ Disc samples of KNN ceramics are fabricated through a process involving solid‐state reaction, sintering, and polarization.^[^ ^3c^ ^]^ KNN, characterized by a complicated orthorhombic composition, demonstrates spontaneous polarization along a specific direction and possesses commendable electromechanical properties (k^p^ = 0.36, d_33_ = 80 pC N^−1^).^[^ ^130^ ^]^ A pivotal step in confirming in vitro biocompatibility involves assessing the material's impact on protein adsorption as well as cell proliferation. The polarized surface of KNN has been validated to promote protein adsorption and enhance cell growth compared to unpolarized KNN.^[^ ^18^ ^]^

Element doping influences the microstructure, macrostructure, and functions of piezoelectric ceramics. In 2021, researchers discovered that the macroscopic tetragonal phase structure formed through multiple doping significantly enhances the piezoelectric abilities of the KNN ceramics.^[^ ^131^ ^]^ In the realm of regenerative medicine, bioelectrical signals play a crucial role in mediating stem cell fate, angiogenesis, and other activities. Exogenous electrical signal has been shown to regulate the directed migration and differentiation of stem cells.^[^ ^132^ ^]^ KNN, classified as one of the perovskite ceramics, features Na^+^ and K^+^ ions occupying eight vertices in the crystal cell, while O^2−^ and Nb^5+^ ions are stationed at the surface and centers of the cube. The piezoelectric properties of KNN are intricately linked to the displacement of Nb^5+^ in response to external forces or external electric field.^[^ ^133^ ^]^ To bolster the potential of KNN‐based ceramics for bone implant applications, researchers have undertaken the elemental doping of KNN (e.g., Li^+^, Ca^2+^, Mg^2+^).^[^ ^134^ ^]^ However, a comprehensive investigation into their cytotoxicity, piezoelectric properties, and osteogenic potentials remains imperative.

As mentioned above, piezoelectric KNN ceramics have excellent piezoelectric properties, which provides ideas for regenerative medicine. Except the application in bone repair, electroactive KNN scaffolds also have great prospects in repairing nerves and myocardium. In 2022, Chen et al.^[^ ^135^ ^]^ used electrospinning to prepare polylactic acid nanofibers and KNN nanowires into 3D piezoelectric scaffolds to realize the “three‐in‐one” function, that is, the composite piezoelectric biomaterial combined the biodegradability, controllable electrical stimulation and tissue engineering porous scaffold simultaneously. In vitro experiments demonstrated that this kind of wireless electrical stimulation generated from ultrasound‐driven 3D piezoelectric nanofibers could differentiate neural stem cells into neurons, while in vivo findings demonstrated that the electrical stimulation delivered through this 3D piezoelectric scaffolds facilitated motor recovery and the regeneration of spinal cord defects when properly irradiated with ultrasound. This study corroborated the vigorous prospect of programmable ultrasound combined with degradable 3D piezoelectric KNN scaffold in tissue engineering.

#### 3.1.3 Zinc Oxide (ZnO)

Zinc oxide (ZnO) crystals exhibit commendable piezoelectric properties derived from the deformation of an asymmetrical crystal structure under external stimulation. Within the asymmetric crystal structure of ZnO, a tetrahedral coordination structure forms between zinc and oxygen atoms. When the electroneutral tetrahedral structure encounters an external force, the center of electric charges shifts, resulting in polarization. This leads to the generation of charge enrichment along the stress direction, creating a piezoelectric potential.

Researchers extensively leverage the piezoelectric abilities of ZnO in tissue regeneration, particularly in nerve regeneration and wound healing. In the realm of scaffold materials for nerve repair, it is crucial to use materials with appropriate morphology, mechanical stability, and the capability for continuous endogenous electrical stimulation to promote nerve growth. Given the intricate structure of neural tissue, the design of materials that mimic the ECM is crucial for guiding neural tissue growth.^[^ ^136^ ^]^ Electrospinning emerges as a viable technique for fabricating nanofibers with a high‐porosity structure akin to the ECM. In 2022, Mao et al.^[^ ^137^ ^]^ successfully prepared a composite comprising ZnO nanoparticles and polycaprolactones (PCL) for peripheral nerve regeneration. ZnO nanoparticles, at biosafety concentrations, were transformed into piezoelectric films through electrospinning, providing endogenous electrical stimulation conducive to sciatic nerve repair.

ZnO emerges as a multifunctional nanomaterial endowed with commendable piezoelectric properties, robust antibiotic performance, rapid reactive oxygen species (ROS) scavenging ability, and the potential to foster cell proliferation.^[^ ^138^ ^]^ Previous investigations have demonstrated that ZnO nanoparticles can significantly augment the expression of fibroblast growth factor (FGF) and vascular endothelial growth factor (VEGF), thereby promoting fibroblast proliferation and angiogenesis.^[^ ^139^ ^]^ In wound healing, a decrease in bioelectrical stimulation around the wound may disrupt collagen fiber deposition, subsequently impacting wound healing and scar formation.^[^ ^140^ ^]^ Consequently, the advancement of piezoelectric adjuncts providing continuous electrical signals holds profound significance for skin regeneration.

In a notable research, Liang et al.^[^ ^141^ ^]^ harnessed the piezoelectric and antibacterial attributes of ZnO nanoparticles to fabricate ZnO‐modified PVDF and sodium alginate (SA) hydrogel piezoelectric scaffolds via 3D printing technology. These piezoelectric composites aimed to mimic and amplify endogenous bioelectricity for the healing of infected wounds. In vitro studies underscored that the incorporation of 0.5% ZnO exhibited commendable biocompatibility and uniform distribution in PVDF, modifying it with stable hydrophilic polarization. In conclusion, ZnO, with its outstanding piezoelectric properties and antimicrobial capabilities, holds promise for widespread application in tissue engineering. Nevertheless, caution is warranted as high concentrations of ZnO can be cytotoxic, necessitating careful consideration of its biocompatibility in specific applications.

#### 3.1.4 2D Piezoelectric Materials

Different from traditional piezoelectric bioceramics, boron nitride is a unique 2D piezoelectric ceramic material.^[^ ^142^ ^]^ Under specific conditions, certain forms of boron nitride, such as boron nitride nanosheets and nanotubes, exhibit piezoelectric properties.^[^ ^143^ ^]^ This 2D characteristic distinguishes boron nitride, as its piezoelectric response is influenced by its nanostructure and the specific conditions applied, including stress and electric fields, making it a candidate of interest in flexible electronics and tissue regeneration.^[^ ^144^ ^]^ The remarkable advantages of boron nitride for tissue regeneration lie in its excellent biocompatibility and high mechanical strength.^[^ ^145^ ^]^ However, boron nitride typically possesses a low piezoelectric coefficient (d_33_ = 0.3 pC N^−1^). Scientists are actively exploring alternative synthesis methods, innovative structural designs, and the preparation of piezoelectric composites to enhance its piezoelectric properties, aiming to make it more viable for applications requiring a stronger piezoelectric response.^[^ ^146^ ^]^ For instance, Qian and coworkers have constructed boron nitride nanosheet‐incorporated PCL scaffolds for neuronal repair, and their piezoelectric coefficients ranged from 3.3 to 25.7 pC N^−1^, which could be comparable to traditional piezoelectric biomaterials.^[^ ^147^ ^]^ Overall, boron nitride's excellent biocompatibility and mechanical strength position it as a potential candidate for tissue engineering applications. While its piezoelectric abilities require further enhancement, boron nitride's potential in tissue regeneration, for example, in bone regeneration and nerve repair scaffolds, is anticipated to be increasingly realized through advancements in composite design and innovative preparation technologies.

### 3.2 Piezoelectric Polymers

Among the multiple ranges of piezoelectric materials, piezoelectric polymers are widely chosen for applications of regeneration medicine because of easy processing, high flexibility and excellent mechanical properties.^[^ ^148^ ^]^ Piezoelectric polymers have high compliance, and their compliance coefficient is ten times that of piezoelectric ceramics, so they are capable of being processed into large and thin films with excellent mechanical strength and toughness, which can endure large impact forces.^[^ ^149^ ^]^ Many piezoelectric polymers have been shown to promote cell differentiation and immunomodulation in answer to mechanical or electrical stimuli.^[^ ^150^ ^]^ The first piezoelectric polymer with piezoelectric effect to be discovered was polyvinylidene fluoride (PVDF) in 1969, and piezoelectric coefficient of the polarized PVDF film was 6–7 pC N^−1^, which was more than 10 times greater than the corresponding value for the polymers discovered at the time.^[^ ^151^ ^]^ With the development of research, researchers have discovered more polymers with piezoelectric effects, like poly‐L‐lactic acid (PLLA), etc., providing more options in developing materials for regenerative medicine.

#### 3.2.1 Polyvinylidene Fluoride (PVDF) and Its Copolymers

Polyvinylidene fluoride (PVDF) stands out as a promising material for tissue engineering owing to its robust piezoelectric abilities and biocompatibility, with a history of over half a century, stemming from its discovery by Japanese scholar Kawai.^[^ ^89^, ^152^ ^]^ The piezoelectric characteristics of PVDF hinge significantly on the polymer structure, encompassing four main phases: α, β, γ, and δ. Notably, only the β phase demonstrates exceptional piezoelectric abilities.^[^ ^153^ ^]^ Upon deformation induced by an external force, the vinylidene fluoride units along the PVDF molecular chain undergo polarization, resulting in charge separation within the material.^[^ ^154^ ^]^ This phenomenon induces a voltage disparity in PVDF, directly proportional to the magnitude of external force or electric signal.^[^ ^155^ ^]^

PVDF manifests multiple crystalline phases, and notably, the ferroelectric β phase demonstrates superior piezoelectric characteristics (k_33_ = 27% and d_33_ = −28 pm V^−1^).^[^ ^156^ ^]^ Despite these attributes, PVDF falls short of practical application demands when compared to ferroelectric lead zirconate titanate (PZT), which boasts higher values for k_33_ and d_33_, reaching 80% and 800 pm V^−1^, respectively. To promote the piezoelectric abilities of PVDF, researchers have undertaken the synthesis of a range of PVDF‐based copolymers through random copolymerization. Notably, the binary copolymer P (VDF‐TrFE), characterized by higher crystallinity (TrFE representing trifluoroethylene), exhibits a substantial enhancement in piezoelectric performance (k_33_ = 37% and d_33_ = −38 pm V^−1^).^[^ ^23^, ^157^ ^]^ Moreover, advancements in research reveal that nanoparticles can promote PVDF's piezoelectric abilities through enhancing polarization and inducing the transition to the β phase.^[^ ^129^, ^158^ ^]^

In the realm of regenerative applications, PVDF and its copolymers find diverse applications ranging from nerve repair of central and peripheral nervous systems^[^ ^159^ ^]^ to the mending of bone defects.^[^ ^160^ ^]^ Nerve defects stand out as one of the gravest consequences of peripheral nerve injuries, necessitating the urgent development of nerve guidance conduits. Piezoelectric films have recently garnered increasing attention in nerve regeneration for their capacity to produce piezoelectricity in answer to mechanical force or ultrasound stimulation, thereby stimulating the proliferation and differentiation of neural stem cells.^[^ ^161^ ^]^ To bolster the regenerative capabilities of NGCs, numerous researchers have implemented various enhancements, such as the incorporation of microporous structures to facilitate cell migration and the careful selection of scaffold materials. In 2020, Cheng et al.^[^ ^162^ ^]^ devised a piezoelectric NGC comprising a 3D PVDF/PCL composite to address rat sciatic nerve defect. The addition of PCL aimed to ameliorate the unsatisfactory biodegradability of PVDF, albeit resulting in a slight reduction in piezoelectric activity due to the PCL blend (the d_33_ values of PVDF, PVDF/PCL, and PCL were ≈30.4, 13.2, and 1.1 p.m. V^−1^, respectively). Inspired by this endeavor, Ma and colleagues developed a novel composite NGC by fabricating a multi‐channel silk fibroin cryogel and subsequently introducing poly(3,4‐ethylenedioxythiophene) (PEDOT) through in situ polymerization. Additionally, a PVDF/poly(L‐lactic acid‐co‐caprolactone) (PLCL) membrane was applied to the outermost layer to induce piezoelectric activity.^[^ ^54^ ^]^ This biomimetic composite scaffold has demonstrated its efficacy in promoting neuronal proliferation, differentiation, and axonal regeneration. However, the piezoelectric abilities of PVDF/PLCL were not explicitly addressed, warranting further validation of in vivo biodegradation.

In addition to its role in nerve regeneration, PVDF stands out as an optimal piezoelectric material for expedited or critical bone repair, because of its notable piezoelectric abilities and exceptional biocompatibility.^[^ ^94a^ ^]^ The intrinsic potential generated by PVDF or its copolymers in response to mechanical forces is adept at mimicking the endogenous electric microenvironment that contributes to bone repair.^[^ ^160^ ^]^ As previously mentioned, PVDF has diverse crystalline phases, including the non‐polar α‐phase, easily obtainable through processing, and the polar β‐, γ‐, and δ‐phases, attainable only through stretching or polarization. The most polar β‐phase of polymer is particularly noteworthy as a crucial piezoelectric and ferroelectric material in bone repair. Research has established that endogenous electrical field significantly fosters the healing of bone defects. Consequently, enhancing the piezoelectric abilities of PVDF and promoting its β‐phase proportion holds considerable implications for bone tissue engineering. Recent findings indicate that composite PVDF nanofibers, incorporating polyhedral oligosilsesquioxane‐epigallocatechin gallate (POSS‐EGCG), significantly elevated the piezoelectric β‐phase of PVDF from 77.4% to 88.1%. This enhancement led to a noticeable augmentation of the osteogenic differentiation of MC3T3 cells.^[^ ^163^ ^]^

Angiogenesis, a critical process during bone formation, has seen limited exploration concerning how piezoelectric biomaterials convert stress into electrical signals to influence vascular cells.^[^ ^164^ ^]^ Chen and colleagues adorned PVDF with ZIF‐8 nanocrystals to achieve sustained release of Zn^2+^, enhancing angiogenesis and antibacterial activity.^[^ ^164b^ ^]^ Furthermore, the incorporation of metal‐organic frameworks (MOFs) could elevate the β‐phase content of PVDF, thereby influencing the conductivity of composite scaffolds. Beyond phase structure modification, enhancing the hydrophilicity of PVDF is pivotal for establishing strong osteointegration.^[^ ^165^ ^]^ Titanium dioxide (TiO_2_) nanomaterials emerge as promising candidates for PVDF modification because of their remarkable structural firmness and hydrophilicity.^[^ ^166^ ^]^ Liu et al.^[^ ^167^ ^]^ has developed a composite PVDF film with 0.3wt% TiO_2_ nanoparticles, significantly increased the osteogenic differentiation of BMSCs compared with pure PVDF, offering a novel perspective on PVDF modification.

#### 3.2.2 Poly‐L‐Lactic Acid (PLLA)

Poly (L‐lactic acid) (PLLA), a biodegradable polymer synthesized from L‐lactic acid monomers, exhibits commendable biocompatibility and biodegradation properties, positioning it as a versatile material in tissue regeneration. The piezoelectric response of PLLA arises from β‐sheet formation during C═O rearrangement under polarization.^[^ ^168^ ^]^ Despite its advantageous characteristics, PLLA's piezoelectric response is comparatively modest when contrasted with traditional piezoelectric materials. This limitation necessitates further modifications to align with the demands of practical applications. Various approaches have been explored to enhance PLLA's piezoelectricity, encompassing alterations in crystallinity, incorporation of fillers, introduction of functionalized groups, and blending nanoparticles onto PLLA film surfaces. In the context of bone regeneration, the piezoelectric and mechanical attributes of PLLA assume a pivotal role, influencing the adhesion and migration of bone cells. Recent investigations have unveiled a controllable aspect of PLLA's piezoelectric effect through the application of different voltage polarities during electrospinning.^[^ ^169^ ^]^ Specifically, elevating positive voltage polarity correlates with a heightened surface potential of PLLA, fostering advantageous adhesion and proliferation of osteoblasts. This intriguing phenomenon can be elucidated by the influence of electrostatic gravity, wherein the negatively charged cell membrane (−10 to −90 mV) exhibits a predilection for adherence to the surface of positively charged films. This behavior, in turn, exerts biological forces, leading to the generation of more endogenous electrical signals that regulate cell proliferation and differentiation.^[^ ^170^ ^]^

As previously mentioned, enhanced the piezoelectric properties and osteogenic induction capacity of PLLA can be realized through incorporating nanoparticles or the doping of bioactive elements. Zheng et al.^[^ ^171^ ^]^ developed composite PLLA scaffolds by introducing Ca/Mn‐doped BaTiO_3_ (CMBT) nanofibers into the PLLA. The addition of CMBT nanofibers proved instrumental in augmenting the piezoelectric properties of standard PLLA. Following immersion in a Fetal Bovine Serum (FBS)‐containing culture medium for 21 days, the d_33_ value transitioned from 0.38 pC N^−1^ (pure PLLA) to 3.5 pC N^−1^ (PLLA/CMBT), approaching physiological piezoelectricity levels (d_33_ = 0.7–2.3 pC N^−1^).^[^ ^15^ ^]^ Beyond BaTiO_3_, the osteogenic characteristics of the composites were further enhanced by incorporating glass fiber‐reinforced hydroxyapatite particles (gHA) into PLLA during the electrospinning process. This aimed to augment the porosity of PLLA fibers and increase the surface area available for cell interactions.^[^ ^127b^ ^]^

Due to its commendable biocompatibility, degradability, controllable 3D structure, and efficient drug slow‐release properties, PLLA has become a potential piezoelectric candidate for nerve regeneration and wound repair in recent years. Leveraging ultrasound to activate the electrical signal of piezoelectric PLLA thin film allows for controlled and sustained endogenous electrical stimulation, facilitating nerve defect repair without transcutaneous intervention. Wu and coworkers have developed piezoelectric composite films using polymers Poly(3‐hydroxybutyrate‐co‐3‐hydroxyvalerate) (PHBV) and PLLA as the matrix, incorporating soluble KNN nanowires as a kind of dopant with high‐performance piezoelectricity.^[^ ^172^ ^]^ The 1:1 PHBV/PLLA blends exhibited outstanding biological and mechanical properties, with the doping of 50% KNN nanowires ensuring their uniform distribution on the composite film's surface. However, during ultrasound treatment, electric charges produced from the encapsulation layer due to friction effects could diminish the output voltage and current.

To fabricate a neural guidance catheter conducive to cell growth, mimicking the extracellular matrix's humid environment, researchers embedded carbon nanotube‐incorporated hydrogel into PLLA, forming a piezoelectric scaffold that promotes Schwann cell growth, myelination, and axon growth. This innovative approach offers an alternative to autologous nerve repair for defects.^[^ ^173^ ^]^ Additionally, the modification of polypyrrole (PPy) enhances PLLA's piezoelectric properties. Xiong et al.^[^ ^174^ ^]^ demonstrated that in situ polymerization of PPy achieves directional distribution of PLLA fibers, with polydopamine (PDA) strengthening the combination of PPy and PLLA. This composite promotes nerve repair by activating the calcium and AMP‐activated protein kinase signaling pathways.

Addressing wound repair, under external stimuli such as mechanical force or ultrasound, piezoelectric materials generate piezoelectric potential, electrochemically reacting with water molecules around the wound to promote ROS production, exhibiting antibacterial activity.^[^ ^175^ ^]^ Inspired by this property, researchers prepared multifunctional piezoelectric nanofibers through electrospinning technology, modified with calcium peroxide to convert ROS produced by piezoelectric catalysis into oxygen, promoting cell proliferation. Combined with the intrinsic photothermal activity of reduced graphene oxide, these nanofibers synergistically enhance the repair of infected wounds.^[^ ^176^ ^]^ In summary, modifying crystallinity, incorporating fillers, introducing functionalized groups, and blending nanoparticles onto PLLA film surfaces can significantly enhance PLLA's piezoelectric activity, offering novel insights for designing composite PLLA scaffolds in regenerative medicine.

#### 3.2.3 Polyhydroxyalkanoate (PHA)

Polyhydroxyalkanoate (PHA) is an intracellular polyester synthesized by bacteria, possessing significant application potential in packaging and medicine because of its environmental protection and biodegradability.^[^ ^177^ ^]^ As a kind of natural biocompatible piezoelectric material, the piezoelectric abilities of PHA are closely associated with their molecular structure and crystalline arrangement, particularly the orientation of their polar molecular chains and the alignment within the crystalline regions.^[^ ^178^ ^]^ Poly(3‐hydroxybutyrate) (PHB) and poly(3‐hydroxybutyrate‐co‐3‐hydroxyvalerate) (PHBV) are the two primary forms of PHA, exhibiting complete biodegradability, excellent biocompatibility, and inherent piezoelectric properties.^[^ ^179^ ^]^ PHB is a homopolymer, while PHBV is produced through copolymerization with other monomers, such as valeric acid. As a result, there are some differences in their properties, with PHBV generally being more flexible and elastic.^[^ ^180^ ^]^ The piezoelectric characteristics of PHB are comparatively weaker compared with other piezoelectric polymers or ceramics, registering only 1.6–2 pC N^−1^.^[^ ^181^ ^]^

In the pursuit of developing piezoelectric scaffolds for tissue regeneration, mechanical strength and piezoelectricity of pure PHB or PHBV fall short of meeting clinical requirements. Consequently, researchers have endeavored to enhance the strength and piezoelectricity of composite PHB scaffolds through the incorporation of inorganic fillers. The in situ synthesis of inorganic fillers within polymers stands out as a commonly employed method for creating hybrid materials.^[^ ^182^ ^]^ Research has demonstrated the in situ synthesis of calcium carbonate (CaCO_3_) through ultrasound on the surfaces of PHB and PHBV in spherulitic aragonite and calcite polymorphs.^[^ ^183^ ^]^ This surface mineralization transforms the materials from hydrophobic to hydrophilic, fostering the adhesion and proliferation of BMSCs. Notably, ZnO, widely recognized for its piezoelectric properties and antimicrobial capabilities, is extensively used as a piezoelectric nanoparticle.^[^ ^184^ ^]^ ZnO modification enhances the piezoelectric response of polymers, potentially maintaining or elevating the piezoelectric properties of PHB compared to non‐piezoelectric CaCO_3_ modification. Additionally, hydrophilic ZnO coatings enhance the wetting ability and cell adhesion of PHB scaffolds, positioning them as promising candidates for scaffold modification.^[^ ^185^ ^]^ In 2019, researchers successfully prepared a composite PHB/ZnO through electrospinning and hydrothermal deposition, significantly elevating the piezoelectric coefficient of PHB from 2.9 ± 0.1 to 13.7 ± 1.6 pC N^−1^.^[^ ^186^ ^]^

In its role as a non‐piezoelectric nanofiller, reduced graphene oxide (rGO) has exhibited the capability to promote the piezoelectric response of PHB owing to its versatile properties.^[^ ^183^, ^187^ ^]^ In its role as a non‐piezoelectric nanofiller, rGO has exhibited the capability to enhance the piezoelectric response of PHB owing to its versatile properties. In 2023, researchers further explored the effects of doping with varying levels of rGO or polyaniline (PANI) on the piezoelectric effect of PHB and the interaction of these materials with BMSCs.^[^ ^129^ ^]^ The study revealed that the addition of rGO or PANI nanofillers to PHB did not impact the adhesion density of BMSCs. Compared to the PHB control group, there was an obvious increase in the aspect ratio of BMSCs attached to the composite scaffold. This suggests that PHB‐based composites hold promising applications in regenerative medicine, particularly in bone regeneration.

#### 3.2.4 Piezoelectric Elastomer

A piezoelectric elastomer is a flexible piezoelectric material synthesized by copolymerizing bio‐based diacids and diols, offering high flexibility and making it suitable for applications in soft tissue regeneration, like skeletal muscle and Achilles tendon repair.^[^ ^188^ ^]^ For instance, Ge and coworkers developed a piezoelectric elastomer (VMLRE) with excellent biocompatibility and stretchability by copolymerizing lactic acid, sebacic acid, butanediol, and itaconic acid to repair volumetric muscle loss.^[^ ^189^ ^]^ The VMLRE's long and straight C═O dipoles within the ester bonds ensured its high piezoelectricity, enabling in situ electrical stimulation to promote myoblast proliferation and differentiation.

Another study developed a piezoelectric elastomer (PPBE) for the repair of skeletal muscle loss through random copolymerization of 1,3‐PDO, 2,3‐butanediol, sebacic acid, succinic acid, and itaconic acid.^[^ ^104^ ^]^ The C═O dipoles in the ester bonds from copolymerization of selected diacids and diols provided PPBE with excellent piezoelectricity, which could be activated by ultrasound and physical exercise after implantation. For tendon rupture repair, researchers developed a piezoelectric elastomer (PETRR) with an elastic modulus of 0.3 MPa and a recoverable strain of up to 300%.^[^ ^190^ ^]^ PETRR exhibited high biocompatibility and piezoelectricity, allowing it to withstand long‐term reciprocating motion, adapting to the physiological demands of the Achilles tendon.

### 3.3 Natural Piezoelectric Materials

Natural biocompatible piezoelectric materials represent an exemplary category within the realm of piezoelectric substances, offering notable advantages such as commendable biocompatibility and stability. In contrast to conventional piezoelectric bioceramics and synthetic polymers, such as BaTiO_3_ and PVDF, the piezoelectricity exhibited by natural piezoelectric materials is relatively modest.^[^ ^181^ ^]^

#### 3.3.1 Cellulose

Cellulose is a naturally occurring polysaccharide widely found in plants. It has shear piezoelectric abilities and a wide spectrum of applications in biomedical sensors and flexible electronic devices.^[^ ^191^ ^]^ At the microscopic level, cellulose is a linear polysaccharide consisting of glucose units that connected by β‐1,4‐glycosidic bonds.^[^ ^192^ ^]^ Its molecular structure comprises rigid crystalline regions and relatively flexible amorphous regions. Within the crystalline regions, the glucose units are arranged in a highly ordered manner, which can lead to charge rearrangement under external stress, thereby exhibiting piezoelectric properties.^[^ ^193^ ^]^ That is to say, when subjected to external mechanical stress, like shear, tension, or compression, these highly ordered microfibrils deform, resulting in molecular polarization.^[^ ^194^ ^]^ This polarization generates electrical charges, producing the piezoelectric effect. As a result, the piezoelectric abilities of cellulose are closely associated with its fiber orientation and degree of crystallinity.

Nevertheless, the weak piezoelectricity of natural cellulose restricts its applications in tissue regeneration. Thus, it is commonly used in conjunction with other piezoelectric polymers, such as PVDF, to form composites that achieve a more robust piezoelectric effect.^[^ ^195^ ^]^ Recently, studies have indicated that the piezoelectric effect of cellulose can be enhanced through physical treatments such as tensile orientation, compressive or shear stress, as well as chemical modification. For example, Szewczyk et al.^[^ ^196^ ^]^ fabricated cellulose acetate fiber scaffolds using electrospinning technology, achieving a piezoelectric coefficient of 6.68 ± 1.70 pmV^−1^ and a high surface potential (718 mV), enabling it to mimic the natural electric environment favorable for osteoblast attachment and growth.

Moreover, the aspect ratio of cellulose fibers directly influences their piezoelectric properties. By optimizing the aspect ratio, the crystallinity, orientation, and mechanical performance of the fibers can be optimized, thereby enhancing their piezoelectric performance^[^ ^197^ ^]^: 1) Cellulose fibers with high aspect ratios generally exhibit better orientation, particularly in nanocellulose materials. Greater fiber orientation leads to a more orderly arrangement of molecular chains along the fiber axis, facilitating easier molecular polarization under external forces, thereby enhancing the piezoelectric effect. 2) Cellulose fibers with a larger length‐to‐diameter ratio tend to exhibit higher crystallinity during preparation, as longer fibers are better able to align and stretch, facilitating the ordered crystallization of molecular chains. Increased crystallinity enhances the degree of polarization of the fibers under stress, thereby improving their piezoelectric properties. 3) Cellulose fibers with higher aspect ratios exhibit greater mechanical strength and elastic modulus, enabling them to undergo stable deformation under external mechanical stress, which facilitates the generation of the piezoelectric effect as well as the accumulation and conduction of charge.

Cellulose‐based piezoelectric composites have found extensive application in bone tissue engineering and wound dressing design. For example, Kordbacheh et al.^[^ ^198^ ^]^ fabricated a piezoelectric composite for bone regeneration by integrating cellulose nanocrystals (CNCs) with piezoelectric nanomaterials BaTiO_3_ and thermoplastic polyurethane (TPU) to construct a piezoelectric composite for bone regeneration. The incorporation of CNCs not only enhanced the piezoelectric performance of the scaffolds but also improved the distribution of BaTiO_3_ and augmented mechanoelectrical transduction capabilities. Furthermore, electrospinning enables the fabrication of cellulose‐based piezoelectric mats for wound healing.^[^ ^199^ ^]^ Therefore, cellulose, whether in crystalline form or processed into films, can be combined with other piezoelectric nanoparticles or polymer to fabricate piezoelectric composites. Moreover, it demonstrates significant potential for applications in diverse tissue regeneration contexts beyond bone repair and wound healing.

#### 3.3.2 Silk Fibroin (SF)

Silk fibroin (SF) is a natural protein primarily derived from silk, which exhibits certain piezoelectric properties, particularly in its nanostructured or thin‐film form.^[^ ^200^ ^]^ This piezoelectric response is attributed to the ordered molecular arrangement that can be achieved under specific conditions, making it a promising material in biomedical devices, sensors, and tissue‐engineered scaffolds. The silk fibroin possesses good biocompatibility and tunable mechanical properties with an innate piezoelectricity of 1 pC N^−1^.^[^ ^201^ ^]^

Multiple treatments have been employed to enhance the piezoelectricity of silk fibroin; for instance, solvent treatment of ethanol (EtOH)‐immersed silk can produce an output voltage of 7 V and 150 nA, resulted from the piezoelectricity of the β phase.^[^ ^200^ ^]^ Moreover, the elongation of silk fibroin results in a higher piezoelectric constant than that of silk fibroin in its original length.^[^ ^202^ ^]^ Using electrospinning technology, silk fibroin can be fabricated in various dimensions to construct piezoelectric silk fibroin scaffolds for tissue regeneration. Researchers have investigated the relationship between fiber dimension, chemical treatment, and the piezoelectricity of silk fibroin, indicating that the electrical field derived from silk fibroin scaffolds can facilitate the maturation of human neural stem cells (hNSCs).^[^ ^203^ ^]^ The composite scaffolds with conductive materials and other piezoelectric polymers, such as modified PVDF, have been applied to accelerate the repair of peripheral nerves, achieving an output voltage of up to 100 mV under ultrasonication.^[^ ^204^ ^]^ Moreover, Yue et al.^[^ ^205^ ^]^ developed a SF‐based wound dressing functionalized with LiNbO_3_ nanoparticles, exhibiting a piezoelectric output voltage of 0.6 V and significantly enhanced tensile strength. In addition to electrospinning and compounding with piezoelectric nanoparticles, SF can also be processed into gradient scaffolds with biomimetic pore architectures via 3D printing or freeze‐drying techniques. These engineered structures efficiently simulate the microenvironment of bone and cartilage regeneration. By adjusting its structure and piezoelectric characteristics, SF can also be designed into a drug‐carrying platform, the rate of drug release can be controlled, thus enhancing the effectiveness of tissue regeneration.

### 3.4 Piezoelectric Composites

A composite piezoelectric material is a composite of two or more kinds of materials (for example, piezoelectric ceramics and piezoelectric polymers), designed to combine the advantages of each component to obtain better overall properties.^[^ ^206^ ^]^ Composite piezoelectric materials hold significant potential in regenerative medicine, as they integrate the abilities of different piezoelectric scaffolds. For instance, piezoelectric ceramics typically exhibit a high piezoelectric response, yet their brittleness and low mechanical strength limit their application. In contrast, piezoelectric polymers offer excellent flexibility and biocompatibility. By combining these materials, the strengths and limitations of each can be balanced, resulting in a composite material with enhanced overall performance. Additionally, compared to single‐component materials, piezoelectric composites generally offer enhanced mechanical strength and toughness, exhibit greater durability, and are especially well‐suited for environments subject to complex loading conditions.

Various treatments have been applied to integrate piezoelectric bioceramics with polymers in constructing piezoelectric composites to develop highly efficient materials that promote tissue regeneration. For example, electrospinning technology can incorporate piezoelectric bioceramics nanoparticles into polymers such as PVDF or PLLA to fabricate a piezoelectric composite membrane that generates in vivo electrical stimulation to promote stem cell differentiation under mechanical stress or ultrasound.

Piezoelectric composites like magnetoelectric materials can control magnetization and electrical charge simultaneously by applying a distant magnetic field, thus providing a manageable magnetoelectric microenvironment for tissue regeneration.^[^ ^207^ ^]^ As an important component of natural biophysical microenvironment, the endogenous magnetoelectric property of natural tissues is very important for osteogenic differentiation,^[^ ^208^ ^]^ chondrogenic differentiation,^[^ ^209^ ^]^ and neurogenic differentiation,^[^ ^210^ ^]^ etc. Restoring the natural magnetic and/or electric microenvironment at the defect sites through implanting magnetoelectric biomaterials is a potential approach to achieve tissue regeneration.^[^ ^211^ ^]^ Moreover, previous researches have shown that the implantation would reduce the 40–50% surface potential of piezoelectric materials, which is not enough to realize optimized osteogenesis in vivo.^[^ ^160^ ^]^ In 2021, Liu et al.^[^ ^212^ ^]^ found that through applying a distant magnetic field, the magnetoelectric scaffolds possess the ability of maintaining surface potential after implanting. The CoFe_2_O_4_ (CFO)/ P(VDF‐TrFE) membranes were fabricated and molecular dynamics (MD) stimulation showed that 10 wt% CFO content membranes significantly achieved the greatest arginylglycylaspartic acid (RGD) sites exposure, which was closely correlated with BMSCs adhesion and spreading area.^[^ ^213^ ^]^ Apart from enhancing osteogenesis, these magnetoelectric membranes also restored the osteoimmune microenvironment by triggering the anti‐inflammatory polarization of macrophages.

Although the above magnetoelectric membranes exhibited excellent biocompatibility and electrical properties, it is still effortful to equip bone defects with an optimal electrical microenvironment due to their low magnetoelectric coupling.^[^ ^214^ ^]^ Therefore, building on previous work, Liu and coworkers have developed multiphase‐structured magnetoelectric materials with better interfacial coupling efficiency by incorporating a core‐shell CoFe_2_O_4_@BaTiO_3_ nanoparticles, which enhances the magnetoelectric coupling of membranes.^[^ ^215^ ^]^ The magnetic force drives ferromagnetic CoFe_2_O_4_, and the force is completely converted to the BaTiO_3_ shell, thereby inducing the interfacial effect on P(VDF‐TrFE) membranes. The strengthened interfacial effects induce β‐phase formation of P(VDF‐TrFE), ultimately indicating that efficient energy conversion was achieved. Moreover, this β‐phase can be repeatedly induced through periodic magnetic field loading. In vivo experiments showed that this smart in situ repowering strategy could enhance bone regeneration under co‐morbidity conditions, including osteogenesis repression induced by dexamethasone and systemic inflammation induced by lipopolysaccharide (LPS).

Other approaches, including 3D printing, solution immersion, melt blending and stretching, self‐assembly, and solution casting, are also promising strategies for constructing piezoelectric composite scaffolds for tissue regeneration. 3D printing technology can combine piezoelectric scaffolds, such as polymers and bioceramic nanoparticles, with bioinks to print complex structures that match tissue shapes and allow adjustments to piezoelectric properties and structural design—features that are ideal for bone regeneration and soft tissue scaffolds, particularly in the creation of complex tissue replacement materials. The solution immersion method involves soaking porous biological materials, such as natural collagen or other biological scaffolds, in a solution containing piezoelectric materials (e.g., BaTiO_3_) to allow piezoelectric nanoparticles to infiltrate the pores of the matrix. This gentle process maintains material biocompatibility and evenly distributes the piezoelectric material within the porous structure, making it eligible for bone regeneration and other hard tissue repair scaffolds, promoting directed cell growth.

For soft tissue repair, piezoelectric materials are mixed with thermoplastic biocompatible polymers, such as PLA or PLGA, and their piezoelectric properties are enhanced through stretching or melt blending to improve the flexibility of the piezoelectric compounds, enabling electrical stimulation for muscle or skin repair. Similarly, piezoelectric particles can be mixed with biocompatible hydrogels through gel injection molding, injected into molds, and then cured to create flexible piezoelectric composites that can form materials in any shape, ideal for large‐area biological scaffolds. The piezoelectric composites produced through this method are especially suited for applications requiring high flexibility, such as in cartilage or tendon regeneration. Self‐assembly of biomolecules, such as collagen or chitosan, with piezoelectric nanoparticles to form composite membranes or fibrous structures can provide materials with microstructures resembling natural tissues, making them suitable for neural tissue repair. The implantation of piezoelectric composites is predominantly in the form of piezoelectric films or hydrogels. **Table** **2** presents a systematic comparative analysis of piezoelectric films versus hydrogels, encompassing their mechanical properties, electrical output, tissue‐specific application scenarios, etc. This comparison serves as a reference framework for the design of piezoelectric composites for tissue regeneration.

**Table 2:** The comparison of piezoelectric films and hydrogels in the application of tissue regeneration.

Classification	Piezoelectric films	Piezoelectric hydrogels
Examples	Pure PLLA/PVDF films; Composite piezoelectric films (combination of BaTiO_3_/ZnO and PLLA/PVDF).	Cs/Gel/PDA‐HA/PDA‐BaTiO_3_ hydrogels; Short PLLA nanofibers (NFs‐PLLA) and collagen hydrogel
Mechanical properties	High mechanical strength; High elastic modulus.	Relatively low mechanical strength; High toughness.
Electrical output	Relatively high piezoelectric coefficient; Stable voltage output.	Adjustable piezoelectric coefficient
Processing Method	Electrospinning technology; Solution casting.	Crosslinking; 3D printing.
Biodegradability	Biodegradation rate is relatively slow (several months to several years).	Adjustable biodegradable rate
Advantages	The preparation method is more convenient; It can be prepared into oriented/biomimetic structure.	Be suitable for the repair of complex‐shaped defects; Injectable
Limitations	The risk of inflammatory response increases due to the slowly biodegradation.	The preparation method is more complex; Relatively lower mechanical strength.
Application scenarios	Cranial defect repair; Wound dressing; Nerve guidance conduit (NGC); Dentin‐pulp regeneration	Cartilage repair; Bone regeneration; The repair of spinal cord injury; Wound dressing; Tendon repair.
References	[162, 171, 225]	[222, 236]

## 4 Molecular Mechanisms for the Piezoelectric Effect on Regulating Tissue Regeneration

**Figure 5:**
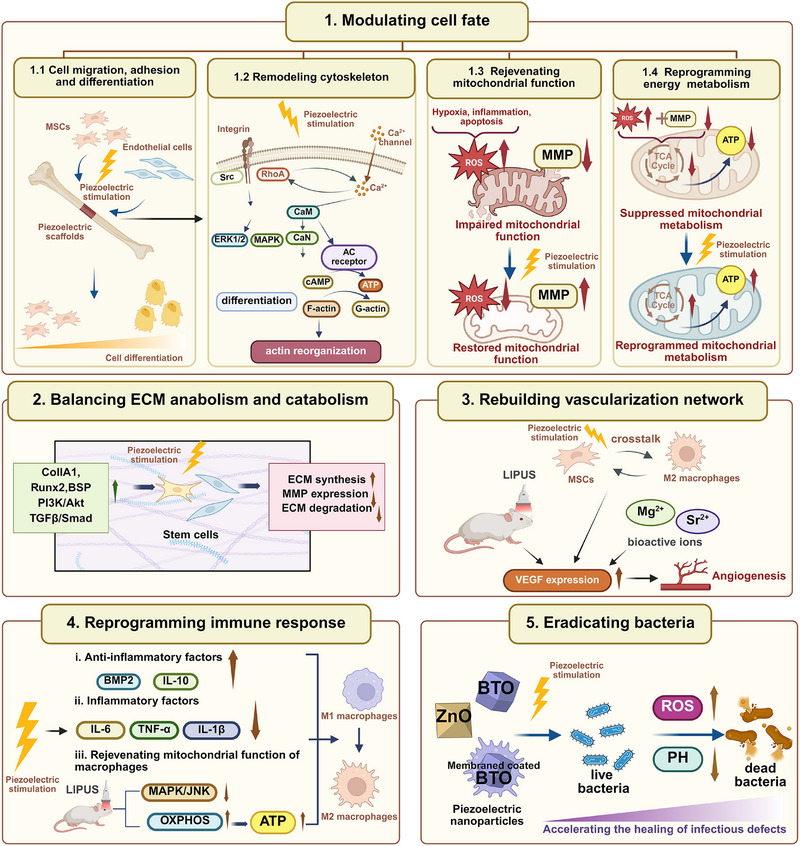
The mechanisms by which the piezoelectric effect generated by piezoelectric scaffolds regulates tissue regeneration. (Created with Biorender.com).

The mechanism of action derived from piezoelectric scaffolds in tissue regeneration primarily relies on their piezoelectric properties, that is, the generation of electric fields under mechanical stimulation, thus modulating cellular behavior and the microenvironment. In this section, we categorize the mechanisms by which piezoelectric materials promote tissue regeneration into five key aspects: modulation of cell fate, ECM formation, angiogenesis, immune microenvironment regulation, and bacterial eradication (**Figure** **5**).

### 4.1 Modulating Cell Fate

#### 4.1.1 Cell migration, Adhesion, and Differentiation

Electrical stimulation generated by piezoelectric biomaterials under mechanical stress, ultrasound, or cell traction has demonstrated positive effects on cell migration, adhesion, and differentiation. Inducing the migration and adhesion of stem cells to defect sites through electrical stimulation could recruit an adequate amount of stem cells to repair tissue defects. Desirable cell adhesion is essential for stem cells to perceive mechano‐electrical signals from piezoelectric biomaterials.^[^ ^237^ ^]^ A hydrophilic layer of the piezoelectric scaffold has been shown to significantly promote cell adhesion. The pattern of piezoelectric scaffolds also acts like a physical stimulation for cell migration and adhesion via influencing the conformation of adsorbed proteins.^[^ ^238^ ^]^ In 2020, Research indicated that the fiber topography of piezoelectric biomaterials interacts with cell adhesion receptors, influencing how cells adhere and determining stem cell fate by altering the morphology or physiology of neural cells.^[^ ^226^ ^]^ Moreover, the amplitude of the electric signal is related to cell migration and adhesion. For example, a study has found that electrical stimulation ranging from 30 to 250 mV mm^−1^ could enhance neural cells’ migration.^[^ ^239^ ^]^ Studies have also shown that the concentration of intracellular Ca^2^⁺ is closely related to the migration and adhesion of MC3T3‐E1 cells, cause the calcium‐sensing receptor can function compatibly with integrins to enhance Ca^2^⁺ signaling and promote cell migration, adhesion, and differentiation.^[^ ^26a^ ^]^

The underlying mechanism of Ca^2^⁺ influx in relation to the migration, adhesion, and differentiation of mesenchymal stem cells may be explained by Rho‐associated protein kinase signaling.^[^ ^240^ ^]^ Specifically, electrical stimulation generated by piezoelectric biomaterials can enhance Ca^2^⁺ influx and activate Ras homolog family member A (RhoA), promoting actin–myosin interactions for focal adhesion and cytoskeletal remodeling.^[^ ^241^ ^]^ As downstream effectors of RhoA, the activation of Mitogen‐activated protein kinase (MAPK) and extracellular signal‐regulated kinase 1/2 (ERK1/2) signaling can trigger a BMP‐2 signaling cascade and upregulate Runx2 expression to enhance osteogenic differentiation.^[^ ^242^ ^]^ In cartilage regeneration, researchers have also demonstrated that piezoelectric PLLA scaffolds can attract ECM proteins to promote chondrocyte adhesion.^[^ ^55^ ^]^ While the functionalization of ECM protein fibronectin on the non‐adhesive fluorinated polymers could enhance cell adhesion.^[^ ^243^ ^]^ The surface potential of piezoelectric scaffolds is closely linked to cell adhesion. For example, a scaffold made of PVDF that underwent corona poling enhanced the adhesion of myoblasts due to its negative surface charge.^[^ ^244^ ^]^ When dopamine (DA) polymerizes on the piezoelectric scaffold, the piezoelectric performance of the composite scaffold may be reduced, while the polydopamine (PDA) could facilitate the adhesion, proliferation, and differentiation of fibroblasts on the materials.^[^ ^245^ ^]^ The 3D construction of piezoelectric materials also influences the adhesion of stem cells. For example, nerve guidance conduit (NGC) could displace autografts and repair nerve defects. Compared to single‐channel NGC, multi‐channel NGC made of piezoelectric biomaterials possesses larger radial permeability that is adaptable for the neural cells' adhesion.^[^ ^246^ ^]^

#### 4.1.2 Remodeling Cytoskeleton

It is widely recognized that applying external direct current (DC) electrical stimulation induces significant remodeling of the actin cytoskeleton, including influencing the filamentous actin (F‐actin) network behavior and fiber bundles.^[^ ^247^ ^]^ To explore the mechanisms by which electrical stimulation drives cytoskeletal reorganization and its association with cell fate, researchers cultured human mesenchymal stem cells (hMSCs) in an electro‐bioreactor exposed to an electric field of 100 mV mm^−2^ for 1 h daily. At day 7, observations revealed that the orientation of the cells and the actin filaments in the cytoskeleton had reorganized perpendicularly to the electric field, particularly around the cathode.^[^ ^248^ ^]^ By applying electrical stimulation to fibroblasts at 50 V, 60 cycles per minute, in a cyclic manner for 0, 2, 5, and 20 h, researchers observed that the fibroblasts’ stress fibers became thicker, and the cells exhibited a contracted morphology. The expression levels of focal adhesion kinase (FAK) and tyrosine‐phosphorylated c‐Src increased over time, suggesting that electrical stimulation influenced cytoskeletal reorganization.^[^ ^249^ ^]^

Integrins contribute to linking the ECM microenvironment to the cytoskeleton by transmitting extracellular signals that induce cellular responses.^[^ ^250^ ^]^ FAK is engaged in the integrin‐enriched adhesion activities, which regulate cell adhesion to the extracellular substance.^[^ ^251^ ^]^ Recent research has shown that the heterogeneous surface potential of piezoelectric membranes can simulate the heterogeneity of the ECM electric potential.^[^ ^187a^ ^]^ This heterogeneity induces a meshwork pattern of fibronectin (FN) for BMSCs adhesion and significantly enhances the traction of actin fibers. These effects promote osteogenesis by upregulating the nuclear transduction of Yes‐associated protein (YAP) and Runx2. Moreover, electrical stimulation can enhance cellular metabolism and accelerate the depletion of adenosine triphosphate (ATP), facilitating cytoskeletal remodeling.^[^ ^252^ ^]^ Cytoskeletal reorganization forms the foundation for driving stem cell differentiation, while ATP production accelerates this process by facilitating the conversion of globular actin (G‐actin) into F‐actin.^[^ ^253^ ^]^ Similarly, direct modifying the topography of piezoelectric scaffolds can regulate cytoskeletal morphology. Researchers have found that neural cells can answer the topological gradient structures of piezoelectric biomaterials by enhancing the expression of cytoskeletal proteins and promoting microtubule assembly.^[^ ^226^ ^]^

#### 4.1.3 Rejuvenating Mitochondrional Function

When tissue damage occurs, the mitochondria of stem cells undergo a series of critical changes, which significantly impact stem cell function and their ability to repair. First of all, tissue damage induces local hypoxia, inflammation, and apoptosis, all of which contribute to elevated levels of mitochondrial ROS. Mitochondria are the main repository of intracellular ROS. High ROS levels can damage mitochondrial membranes, trigger mutations in mitochondrial DNA, and further impair mitochondrial function. In addition, the stressful environment created by high ROS levels may lead to a decline in mitochondrial membrane potential (MMP), thereby weakening the energy conversion efficiency of mitochondria. Disruption of MMP can further activate the apoptosis signaling pathway, adversely affecting cell survival and function. Previous studies have indicated that an optimum level of ROS is beneficial for cell migration by activating redox signaling and modulating the cytoskeleton.^[^ ^254^ ^]^ The generation of MMP is closely linked to the electrochemical gradient established by the mitochondrial electron transport chain, coupled with a sequence of redox reactions. Research has demonstrated that an appropriate degree of electrical stimulation can regulate MMP levels and reduce ROS production, thereby promoting stem cell differentiation. For instance, electrical stimulation resulting from the polarization of a P(VDF‐TrFE) membrane (d_33_ = 10 pC N^−1^) has been shown to produce the lowest ROS levels and the highest MMP levels, facilitating the osteogenic differentiation of BMSCs.^[^ ^53^ ^]^ Moreover, researchers have also discovered that a piezoelectric PCL/ZnO scaffold can enhance osteogenesis by regulating intracellular ROS levels and promoting angiogenesis.^[^ ^255^ ^]^ Thus, it is hypothesized that the surface potential of piezoelectric scaffolds may influence mitochondrial function via altering the oxygen metabolism of stem cells, and ultimately regulate cell fate.^[^ ^256^ ^]^

#### 4.1.4 Reprogramming Energy Metabolism

Apart from high ROS production and the disruption of MMP, mitochondrial metabolism may be significantly suppressed in the stressful environment of tissue damage, as evidenced by decreased activities of the tricarboxylic acid (TCA) cycle and oxidative phosphorylation (OXPHOS). Insufficient energy production can impair the proliferative and differentiation potential of stem cells, thereby reducing their capacity for tissue repair. At the same time, in the damaged environment, stem cells may shift from oxidative metabolism to glycolysis. This metabolic reprogramming may serve as an adaptive response; however, prolonged metabolic imbalance can negatively impact the functional state of stem cells. To rejuvenate mitochondrial metabolism in impaired stem cells, electrical stimulation generated by piezoelectric scaffolds has demonstrated therapeutic potential. For example, as an energy reservoir for cellular activities, ATP can trigger a signaling cascade that facilitates cell differentiation. Electrical stimulation can facilitate the migration of protons across mitochondrial membranes, where they bind with ATP synthase to drive ATP production.

Furthermore, Ca^2^⁺ influx induced by electrical stimulation can enhance this process by activating Ca^2^⁺‐sensitive dehydrogenases and ATP synthase.^[^ ^257^ ^]^ The oscillation of ATP is linked to cyclic adenosine monophosphate (cAMP) kinetics, and increased cAMP levels induced by electrical stimulation can activate AMP‐activated protein kinase (AMPK), protein kinase A (PKA), and liver kinase B1 (LKB1), thereby regulating the cytoskeleton and metabolism of stem cells.^[^ ^258^ ^]^ Recent studies have confirmed that piezoelectric stimulation can induce intracellular Ca^2^⁺ synthesis and elevate MMP through piezoelectric potential‐evoked electron transfer.^[^ ^39^ ^]^ Such positive feedback between Ca^2^⁺ influx and ATP production promotes cytoskeletal reorganization and triggers the osteogenesis of inflammatory PDLSCs. Thus, it can be concluded that electrical stimulation produced from piezoelectric biomaterials can regulate stem cell fate by rejuvenating their mitochondrial function, offering a novel therapeutic strategy for tissue regeneration.

### 4.2 Balancing Extracellular Matrix (ECM) anabolism and catabolism

Extracellular matrix (ECM) is a complicated network structure located outside cells. It supplies physical support to cells and plays a pivotal part in biological processes like cell signaling, proliferation, migration, and differentiation. Tissue damage can significantly impair the function and structure of the ECM, hindering cell function recovery and tissue repair, including the degradation of ECM components, a reduction in elastic modulus, and the interference of matrix fragments with tissue repair.^[^ ^259^ ^]^ Electrical stimulation has been corroborated to enhance stromal reconstitution and stimulate the synthesis of new ECM by cells.^[^ ^260^ ^]^ As previously mentioned, the electrical field produced by piezoelectric materials can facilitate the migration of stem cells to injury sites. Additionally, it improves cell proliferation and differentiation by regulating cell membrane potential and activating Ca^2^⁺ influx, thereby accelerating the synthesis of new ECM.^[^ ^132b^ ^]^ As an exogenously implanted scaffold, piezoelectric materials can stimulate the restoration of ECM stiffness by altering the extracellular mechanical environment and inducing cells to secrete ECM with a high elastic modulus.

The constitution of the ECM differs considerably across various tissues in the body; thus, the mechanisms through which piezoelectric signals promote ECM synthesis must be discussed individually. For example, in bone tissue repair, electrical stimulation induced by piezoelectric materials has been demonstrated to elevate the expression levels of ECM‐related genes (e.g., ColIa1, Runx2, BSP).^[^ ^172^ ^]^ The upregulated expression of these genes directly facilitates the biosynthesis of ECM components of bone, like type I collagen. In addition, incorporating bioactive glass material (BGM), which mimics the components of the bone extracellular matrix, into the piezoelectric material can create a composite that markedly enhances the mineralization of the ECM at bone defect sites.^[^ ^261^ ^]^ As exogenous implanted scaffolds, piezoelectric materials can stimulate the restoration of ECM stiffness by modifying the mechanical environment and inducing cells to secrete ECM with a high elastic modulus.^[^ ^21^ ^]^ Furthermore, electrical stimulation generated by piezoelectric materials, either spontaneously or under external input, can activate integrins on the surface of stem cell membranes, strengthen cell‐ECM interactions, and promote the synthesis of new ECM.^[^ ^187a^ ^]^ Moreover, certain piezoelectric scaffolds also exhibit biophysical cues like those of the bone tissue ECM. For instance, Zhang et al.^[^ ^262^ ^]^ developed a PMMA/PEI/PVDF scaffold with a hierarchical structure and viscoelastic abilities resembling the natural ECM microenvironment, facilitating bone defect regeneration.

As a key strategy to promote the synthesis of cartilage ECM, electrical stimulation produced by piezoelectric scaffolds can suppress the expression and activity of matrix metalloproteinases (MMPs), thereby preventing excessive ECM degradation. As mentioned before, natural ECM of cartilage inherently exhibits a piezoelectricity ranging from 0.2 – 2 pC N^−1^.^[^ ^263^ ^]^ Therefore, cartilage ECM itself is a piezoelectric material that can be utilized for repairing cartilage defects. Building on this concept, Liu and colleagues have employed piezoelectric cartilage‐decellularized ECM to promote chondrogenic differentiation, with an output voltage of 3 mV and a current of 0.25 µA.^[^ ^224^ ^]^ Simultaneously, electrical stimulation activates the energy metabolism of chondrocytes through the PI3K‐AKT signaling pathways, enhancing the energy supply and protein transcription required for cartilage ECM synthesis. Moreover, applying controlled low‐pulse ultrasound (1 MHz at 250 mW cm^−^^2^ for 5 min every other day over 10 days) generates piezoelectric signals that reduce the matrix metallopeptidase 13 (MMP13)/ tissue inhibitor of metal protease 1 (TIMP1) ratio and induce the anabolic factor TIMP1, thereby mitigating ECM degradation.^[^ ^264^ ^]^ Furthermore, the TGF‐β/Smad pathway enhances chondrocyte proliferation and cartilage matrix synthesis.^[^ ^263^ ^]^ Electrical stimulation significantly facilitates the translocation of TGF‐β1 via the calcium/calmodulin pathway, hence enhancing cartilage formation.^[^ ^265^ ^]^ By implanting piezoelectric hydrogel into cartilage defects, ultrasound‐activated electrical stimulation can induce TGF‐β1 expression, facilitating ECM deposition and chondrification.^[^ ^222^ ^]^

Electrical stimulation generated by piezoelectric PVDF can also create an ECM‐like microenvironment, inducing neuron‐like differentiation in BMSCs.^[^ ^7c^ ^]^ Researchers have demonstrated that aligned PCL fibers can simulate the organized constitution of the ECM and promote ECM remodeling both at wound sites and blood vessel.^[^ ^219^, ^234^ ^]^ Thus, piezoelectric materials influence cell signaling, secretion, migration, and the balance of matrix degradation through electrical stimulation, significantly promoting the reconstruction of damaged extracellular matrix. This mechanism offers a novel research direction and therapeutic strategy for tissue regenerative medicine.

### 4.3 Rebuilding Vascularization Network

Angiogenesis is indispensable in tissue regeneration, as it ensures sufficient oxygen and nutrient supply, while facilitating cytokine transport and cell migration. Some external electrical stimulation can mimic endogenous electrical fields, thereby triggering cell migration, proliferation, and differentiation, ultimately promoting faster angiogenesis and expediting tissue regeneration.^[^ ^266^ ^]^ Piezoelectric biomaterials, by converting mechanical stress into electrical stimulation, can generate external electrical fields, playing a pivotal role in regenerative medicine, especially in the repair of bone, skin, and nerve defects. For instance, osteogenesis and angiogenesis have been shown to work synergistically during bone regeneration, because of the intricate crosstalk among MSCs, osteoblasts, and endothelial cells.^[^ ^267^ ^]^ M2 macrophages can improve the proliferation of endothelial cells by secreting IL‐10, BMP2, and VEGF, which play a crucial role in angiogenesis and bone regeneration.^[^ ^208^ ^]^ In addition, the electrical activity produced by piezoelectric biomaterials has been shown to induce the secretion of FGF‐2, which enhances angiogenesis through the MAPK/ERK pathway.^[^ ^30b^ ^]^ Excessive oxidative stress in bone defects impedes the progression of osteogenic differentiation and vascularization by reducing the expression of VEGF.^[^ ^268^ ^]^ Piezoelectric biomaterials have been confirmed to have the therapeutic effect of regulating angiogenesis while simultaneously reducing oxidative stress.^[^ ^269^ ^]^

Electrical stimulation can promote vascular dilation and activate vascular endothelial cells near fracture necrosis, facilitating the restoration of blood supply to defect sites.^[^ ^135^ ^]^ However, not all types of electrical stimulation are effective in upregulating VEGF expression and promoting angiogenesis.^[^ ^270^ ^]^ For example, compared to direct current electrical stimulation, altering electrical stimulation generated by piezoelectrical biomaterials under mechanical forces has shown great potential in promoting VEGF expression.^[^ ^271^ ^]^ Moreover, the ultrasound intensity used to stimulate the energy conversion of piezoelectric biomaterials could also affect the therapeutic effect of angiogenesis. As an additional mechanical stimulation, researchers have demonstrated that low‐intensity pulsed ultrasound (LIPUS) can upregulate the mRNA level of VEGF‐A, thereby promoting the maturation of newly formed bone and enhancing angiogenesis.^[^ ^272^ ^]^

Apart from the types of electrical stimulation and the intensity of mechanical stimulation, the modification of bioactive ions into piezoelectric biomaterials could also enhance the effect of angiogenesis. For instance, Mg^2+^ has been evidenced to promote angiogenesis.^[^ ^273^ ^]^ Building on this observation, researchers have incorporated Mg^2+^ into piezoelectric scaffolds, with its release promoting angiogenic differentiation and facilitating bone formation.^[^ ^43^ ^]^ Similarly, Sr^2+^ has not only been demonstrated to stimulate the differentiation of dental pulp stem cells (DPSCs) into odontoblasts but also to enhance the secretion of proangiogenic VEGF. By incorporating Sr^2+^ into the piezoelectric P(VDF‐TrFE), electrical stimulation coupled with the release of bioactive Sr^2+^ ions produces an enhanced therapeutic effect on dentin regeneration.^[^ ^57^ ^]^

Angiogenesis is also a crucial factor in remedying injured spinal cords and peripheral nerves.^[^ ^274^ ^]^ In situ electrical stimulation produced by piezoelectric scaffolds has been confirmed to promote neurogenesis and endogenous angiogenesis in the spinal cord injury lesion.^[^ ^135^ ^]^ H&E staining observed 12 weeks after the implantation of the piezoelectric material PPy/PDA/PLLA into the peripheral nerve defect showed that the longitudinal incisions exhibited a vessel‐like appearance like the autograft group, indicating that electrical stimulation facilitated angiogenesis, ultimately enhancing nerve regeneration.^[^ ^174^ ^]^ As for wound repair, electrical stimulation can significantly accelerate wound closure by upregulating vascularization and stimulating the expression of α‐SMA, AKT, and ERK.^[^ ^275^ ^]^ To enhance infected wound repair, macrophage membrane‐coated piezoelectric nanoparticles (BaTiO_3_@MMSa) were applied and demonstrated healing properties by enhancing collagen distribution and promoting angiogenesis.^[^ ^217^ ^]^

In addition, the electrical field generated by piezoelectric biomaterials can also enhance endothelial cell viability and promote their migration to form capillary networks facilitating angiogenesis.^[^ ^231^ ^]^ Angiogenesis also contributes to the repair of skeletal muscle loss, as enhanced angiogenesis provides tissue with sufficient oxygen and nutrients, thereby facilitating muscle repair. Local electrical stimulation generated by a piezoelectric elastomer under mechanical force has been evidenced to enhance the myogenic differentiation and angiogenesis.^[^ ^104^ ^]^ In post‐myocardial infarction (MI) cardiac function repair, angiogenesis facilitates oxygen and nutrients supply to restore cardiomyocytes (CMs) in infarcted areas. Following the implantation of piezoelectric PCL/PVDF scaffolds in the myocardial infarction region, the in situ electrical stimulation produced by piezoelectric scaffolds under mechanical force recruited vascular endothelial cells to migrate into the gap, promoting angiogenesis.^[^ ^233^ ^]^

In conclusion, piezoelectric materials generate an electric field through mechanical‐electrical conversion, which, through mechanisms such as upregulating angiogenic factors VEGF, promoting cell function, and releasing bioactive ions, accelerates angiogenesis and ultimately supports tissue regeneration. This mechanism has demonstrated broad applications in bone repair, nerve injury, muscle regeneration, and skin regeneration.

### 4.4 Reprogramming Immune Response

In tissue regeneration, piezoelectric materials not only promote cell migration, adhesion, and differentiation, ECM metabolism, and angiogenesis through direct and indirect function of electrical stimulation, but also profoundly influence tissue repair by regulating immune responses. In the field of bone regeneration, the electric field produced from piezoelectric scaffolds modulates macrophage polarization, inducing the polarization of anti‐inflammatory M2 subtypes and enhancing the secretion of anti‐inflammatory and growth factors, like BMP2 and IL‐10, thereby improving the osteoimmunomodulatory microenvironment to regulate stem cell recruitment, vascularization, and bone repair.^[^ ^276^ ^]^ The modification with polydopamine (PDA) has imparted the composite piezoelectric scaffolds with enhanced immunomodulatory functions, improved cell adhesion, and augmented ROS‐scavenging abilities.^[^ ^277^ ^]^

The crosstalk between macrophages and MSCs within the microenvironment has been demonstrated to play a critical role in determining the fate of bone regeneration, as the polarization of macrophages promotes the recruitment of MSCs and facilitates osteogenesis by secreting C‐C motif chemokine ligand 2 (CCL2), C‐X‐C motif chemokine ligand 8 (CXCL8), and stromal cell‐derived factor 1 (SDF‐1).^[^ ^278^ ^]^ In the later stage of osteogenesis, M2 macrophages dominate the process, significantly enhancing osteogenesis and mineralization.^[^ ^279^ ^]^ Therefore, delayed or impaired bone healing, particularly in a diabetic context, is primarily attributed to an excessive number of M1 macrophages, resulting in an overwhelming inflammatory response.^[^ ^280^ ^]^ To facilitate diabetic bone healing, it is important to promote the homing of MSCs by M1 macrophages in the early stage and to enhance osteogenic differentiation through adequate M2 macrophages in the later stage. This hypothesis has been validated by the application of piezoelectric membranes under varying ultrasound intensities during different phases of diabetic bone healing.^[^ ^44^ ^]^

Furthermore, the mechanisms underlying the electric field produced by piezoelectric scaffolds have been partially elucidated through RNA sequencing. Wu and colleagues discovered that the electrical stimulation generated by BaTiO_3_/Ti (poled) implants under LIPUS significantly inhibited the MAPK/JNK signaling pathways while promoting oxidative phosphorylation (OXPHOS) and ATP production in macrophages.^[^ ^281^ ^]^ These effects contribute to regulating the osteoimmunomodulatory microenvironment and enhancing osteogenesis. In the field of cartilage repair, electrical stimulation generated from piezoelectric hydrogels has been corroborated to alleviate osteoarthritis‐related inflammation by inhibiting the expression of TNF‐α in macrophages.^[^ ^222^ ^]^ During nerve injury, inflammatory responses accompanied by lipid peroxidation, acid‐base disturbances, ROS accumulation, and the release of excessive inflammatory factors activate macrophages, leading to glial scars formation that hinder the repair of neurological tissue.^[^ ^282^ ^]^ To mitigate excessive ROS and regulate immune responses in nerve injury, a piezoelectric‐catalytic nanocomposite has been engineered to generate hydrogen (H_2_), effectively suppressing the production of inflammatory factors and thereby facilitating the rectification of structure and function of spinal cord injury.^[^ ^229^ ^]^

As for the wound healing, electrical stimulation generated by piezoelectric scaffolds can also interferes the inflammation stage and modulate the cell behaviors of immune cells like macrophages to promote wound healing.^[^ ^56^ ^]^ For example, the piezoelectric PVA/PVDF hydrogel has demonstrated the therapeutic outcome in diabetic wound repair by promoting the transformation of macrophages into anti‐inflammatory M2 subtype and inhibit the secretion of inflammatory factors like IL‐6, IL‐1β, and TNF‐α in wound area.^[^ ^232^ ^]^ Similarly, the piezoelectric elastomers VMLRE and PPBE have also been demonstrated to generate an electric field under mechanical stress, modulating macrophage polarization toward the pro‐regenerative M2 subtype to promote skeletal muscle tissue repair.^[^ ^104^, ^189^ ^]^ The mitochondrial function and ATP synthesis in macrophages are enhanced by electrical stimulation induced by the deformation of piezoelectric BaTiO_3_ nanoparticles, promoting the healing of periodontal bone defects associated with periodontitis.^[^ ^39^ ^]^ When natural heat suffers from myocardial infarction (MI), the recruitment of macrophages and secretion of inflammatory factors will lead to pro‐fibrotic process and scar formation, which blocks the cardiac repair.^[^ ^283^ ^]^ Thus, a piezoelectric scaffold PCL/PVDF was specifically designed to reduce the inflammatory response of macrophages in rat MI models through generating electrical field under physiological conditions to enhance cardiac regeneration.^[^ ^233^ ^]^

However, in the process of corneal repair, inflammatory cytokines and chemokines secreted by immune cells like macrophages and neutrophils drive the differentiation of quiescent keratocytes into fibroblasts, promoting corneal repair.^[^ ^235^ ^]^ The piezoelectric field generated by piezoelectric contact lenses during mechanical blinking modulates the immune microenvironment and promotes corneal clarity restoration. In conclusion, electrical stimulation generated by piezoelectric materials demonstrates significant potential in modulating inflammation, facilitating cellular repair, and enhancing the regenerative microenvironment through interactions with immune cells. This offers a novel strategy in regenerative medicine for repair of bone defects, nerve regeneration, and the restoration of skin, myocardium, and corneal tissues.

### 4.5 Eradicating Bacteria

Tissue damage resulting from car accidents, trauma, surgeries, or chronic diseases disrupts the physical barriers of skin and bones, facilitating bacterial invasion into previously sterile tissues, triggering excessive inflammation at the injury site, and hindering tissue healing or leading to chronic non‐healing wounds. Piezoelectric scaffolds produce electric fields or potential differences when subjected to mechanical force, ultrasound stimulation, or other external forces, to exert antibacterial effects and facilitate tissue regeneration by directly sterilizing pathogens or modulating the redox microenvironment at the site of tissue injury.^[^ ^284^ ^]^ Piezoelectric nanoparticles, such as ZnO and BaTiO_3_, possess inherent antibacterial and regenerative properties. As a traditional piezocatalyst with excellent piezoelectric performance, BaTiO_3_ nanoparticles are frequently utilized by researchers in tissue regeneration due to their antibacterial properties. Lei and coworkers developed a sulfur‐doped, oxygen‐defective BaTiO_3_ to achieve enhanced bacterial eradication and promote infected bone healing.^[^ ^45^ ^]^ The underlying mechanism involves oxygen defects, which increase the electron density of the material and enhance charge transfer efficiency. Additionally, sulfur doping can modulate the band gap of BaTiO_3_, enhancing ROS generation and antibacterial efficacy. In vitro antibacterial performance further demonstrated that ultrasound stimulation also exhibited a negligible antibacterial effect, with an antibacterial efficiency of 4.43%. Liu and coworkers designed a piezocatalytic BaTiO_3_ hydrogel for the treatment of bacteria‐infected wounds.^[^ ^216^ ^]^ The strong electric field generated by BaTiO_3_ efficiently generates ROS to eradicate bacteria and promote the wound healing.

Moreover, to enhance the antibacterial properties of BaTiO_3_, photodynamic therapy (PDT) leverages ROS generation as a minimally invasive approach.^[^ ^285^ ^]^ Such a piezophototronic strategy has been implemented by modifying titanium dioxide (TiO_2_) nanomaterials and gold (Au) nanorods with BaTiO_3_ to enhance photodynamic bacterial eradication for infected wound healing.^[^ ^230^ ^]^ Periodontal disease is a common inflammatory disorder primarily caused by bacterial infection, accompanied with progressive destruction of periodontal tissues.^[^ ^286^ ^]^ Incorporating piezoelectric BaTiO_3_ into injectable hydrogel has demonstrated therapeutic efficacy by remodeling the imbalance redox system to deter bacteria for the treatment of periodontitis.^[^ ^39^, ^218^ ^]^ Apart from BaTiO_3_, the piezoelectric nanomaterial ZnO may alter the local pH of defect sites, typically acidifying the environment under the electric charge.^[^ ^287^ ^]^ Reduced viability of bacteria in an acidic environment thereby enhances its antimicrobial efficacy. Liang et al.^[^ ^141^ ^]^ have combined piezoelectric ZnO and PVDF into sodium alginate (SA) hydrogel for killing bacteria, ultimately accelerating wound healing and preventing scar formation.

Piezoelectric polymers like PLLA and PVDF also exhibit inherent electrically driven antibacterial properties. For example, the electrical charge produced by PLLA scaffolds has been shown to inhibit bacterial multiplication through negative charge and improve skin repair through positive charge.^[^ ^56^ ^]^ This outcome could be attributed to the surface charge of piezoelectric scaffold and the local high electric field, which electrostatically attracts bacteria to the material's surface and mechanically disrupts their cell membranes. While the electrical field generated by the deformation of PVDF under mechanical force also demonstrated antibacterial effect for encouraging the repair of infected and diabetic wound healing.^[^ ^220^, ^231^ ^]^ Moreover, the addition of cetyltrimethylammonium bromide (CTAB) onto PVDF conferred the piezoelectric scaffolds enhanced antibacterial activity through its quaternary ammonium cations.^[^ ^41^ ^]^ With the development of membrane coating technology, researchers have discovered that macrophage pre‐activated by *Staphylococcus aureus* (MM_sa_) could specifically target infected areas. By coating MMsa on piezoelectric BaTiO_3_ nanoparticles, this targeted nanosystem could rapidly generate ROS for bacterial eradication under ultrasound irradiation while accelerating the healing of infected wounds.^[^ ^217^ ^]^ All in all, the antibacterial mechanism of piezoelectric materials relies primarily on their piezoelectric properties, which directly or indirectly exert antibacterial effects by generating excess ROS, disrupting bacterial cell membranes, altering surface charges, or modifying the local pH environment. These materials demonstrate significant potential for application in tissue regeneration.

Although some piezoelectric nanoparticles like BaTiO_3_ and ZnO confer certain antibacterial properties, their limited local clearance, undefined in vivo metabolic pathways, and associated biocompatibility concerns significantly impede their clinical translation. As typically cationic peptides with broad‐spectrum antibacterial activity, antimicrobial peptides (AMPs) exhibit a lower propensity to induce bacterial resistance, superior immunomodulatory effects, and enhanced biodegradability.^[^ ^288^ ^]^ Recently, researchers identified the peptide FFRKSKEK (FFRK8) from a piezoelectric diphenylalanine (FF) peptide library, which possesses low toxicity, hydrophobicity, and net positive charge.^[^ ^289^ ^]^ Full‐atom molecular dynamics simulations revealed that US potentiated the membrane‐penetrating capability of FFRK8. Their piezoelectric polarization generated ROS and disrupted the bacterial electron transport chain. In a recalcitrant goat model of spinal infection, sonosensitized peptides demonstrated superior efficacy compared to vancomycin. This finding suggests that US‐activated antimicrobial piezoelectric peptides may represent a promising strategy for combating antibiotic‐resistant infections in tissue regeneration.

## 5 Applications of Piezoelectric Biomaterials in Regenerative Medicine

Piezoelectric materials have garnered considerable attention in regenerative medicine due to their exceptional ability to transform mechanical stress into electrical fields. Endogenous piezoelectricity, inherently present in various human tissues, plays a pivotal role in regulating activities like cell differentiation, ECM formation, angiogenesis, immunomodulation, and antibacterial activity. However, tissue defects caused by trauma, infections, tumors, or other pathological factors disrupt this endogenous electrophysical microenvironment, thereby impeding the natural tissue regeneration process. Recently, a diverse range of piezoelectric biomaterials has been specifically engineered to restore the disrupted electrophysical microenvironment at defect sites, thereby promoting tissue repair. These biomaterials are systematically categorized according to the types of tissues they aim to repair and their associated stimulation strategies, presenting innovative insights and directions for future advancements in regenerative medicine.

### 5.1 Piezoelectric Biomaterials for Bone Regeneration

Bones form the primary structure of the skeletal system, providing structural support and acting as levers for muscles to facilitate movement. The ECM of bone is enveloped by parallel, interwoven collagen fibers and other amorphous inorganic substances, primarily calcium phosphate in the form of HAp. The collagen fibers supply tensile strength and flexibility, while inorganic minerals endow the bone with rigidity and ability to bear weight.^[^ ^290^ ^]^ Notably, the aligned collagen fibers in bone contribute significantly to its piezoelectric properties. This phenomenon is crucial for bone growth and the repair of bone defects or fractures. When subjected to mechanical stress, the piezoelectric effect generates electric charges, which are thought to stimulate bone remodeling and healing by influencing the activity of bone cells.^[^ ^63^ ^]^ In the 1960s, researchers discovered that stress applied to bone could generate local potential along collagen fibers, providing a stimulus in the vicinity for bone cells.^[^ ^291^ ^]^

As previously mentioned, the piezoelectric nature of collagen fibers is attributed to their nonsymmetric structure. Investigations have revealed a repeated piezoelectric pattern in single Type I collagen fibrils, which is inversely correlated with the periodic structure that exhibits the characteristic D‐spacing of the fibrils. This pattern endows collagen with the ability to produce electric charges under mechanical force, which in turn impacts bone growth and repair.^[^ ^61^, ^292^ ^]^ Additionally, changes in ionic strength and viscosity can alter the strain‐generated potential (SGP) of bone.^[^ ^293^ ^]^ These factors influence how mechanical stress is converted into electrical signals within bone tissue. As a result, bone's piezoelectricity does not operate in isolation but may also affect streaming potential by altering the magnitude of the zeta potential under mechanical stress. This interaction between piezoelectric effects and fluid flow within the bone matrix plays a big part in how bone senses and responds to mechanical forces, potentially promoting the processes of bone remodeling and regeneration.^[^ ^9b^ ^]^

Electrical field within bone can modulate bone cell behavior by facilitating charged ions’ flow and ion gradient formation.^[^ ^294^ ^]^ The endogenous electrical field or electrical currents generated by piezoelectric materials under mechanical stress or external ultrasound stimulation play a pivotal role in bone regeneration by enhancing cell–cell communication.^[^ ^109^ ^]^ Understanding the piezoelectric abilities of bone has brought with investigation of the effects of electric field on bone cells. Researches have indicated that electric fields can initiate the differentiation of BMSCs into osteoblasts.^[^ ^295^ ^]^ Bone regeneration includes a series of complicated processes, containing the osteogenic differentiation, angiogenesis, and protection against infection.^[^ ^296^ ^]^ Piezoelectric biomaterials, such as piezoelectric bioceramics, biopolymers, and their composites, can convert mechanical force into an electrical field. This conversion influences surface receptors distribution on BMSCs, facilitates opening of calcium ion channels, and activates intracellular calmodulin. These processes collectively promote osteogenic differentiation, driving the differentiation of BMSCs into osteoblasts, which are critical for bone formation.^[^ ^297^ ^]^ In addition to the piezoelectric abilities of materials, structure arrangement, mechanical properties, and porosity of piezoelectric scaffolds could also affect cell adhesion and differentiation.^[^ ^42^ ^]^ In this part, we will summarize the recent advancements about piezoelectric materials for bone regeneration (**Table** **3**).

**Table 3:** Applications of piezoelectric biomaterials in bone regeneration.

Piezoelectric biomaterials	Stimuli	Piezoelectricity	Major outcomes	Disadvantages	Refs.
Sulfur‐doped BaTiO_3_	Ultrasound	d_33_ = 13.95 pm V^−1^	Antibacterial, anti‐inflammatory and promoted bone regeneration	Lack of stent material for in vivo implantation	[45]
P(VDF‐TrFE)	N/C	d_33_ = 10 pC N^−1^	Improving energy metabolism of BMSCs	In vivo Surface potential stability was lacking	[53]
Random annealed PVDF	N/C	Output voltage = 0.9 V	Remodeling the cytoskeleton of BMSCs	Evaluation of the effectiveness of in vivo implantation was lacking	[21]
P(VDF‐TrFE)/Bioactive glass	N/C	N/C	Improving the proliferation, adhesion, osteogenesis	Evaluation of piezoelectricity was insufficient	[261]
PHBV/PHA/BaTiO_3_	N/C	Output voltage = 11.2 V	Enhancing osteogenesis, inducing M2 macrophage polarization	Lack of description of biodegradability	[277]
BaTiO_3_ nanofibers (BTNF)/ P(VDF‐TrFE)	N/C	d_33_≈7.91 pC N^−1^	Enhancing osteogenesis by promoting nuclear translocation of YAP/Runx2	The stability of surface potential heterogeneity in vivo was not described	[187a]
HA/PDA/PLLA	Ultrasound	d_14_ = 1.82 pC N^−1^	Activating cell membrane calcium channels and PI3K signaling pathways	N/C	[359]
PCL/KNN@PDA	Controllable dose ultrasound	Output voltage = 4.4 V	Promoting recruitment of BMSCs and inducing M2 macrophages polarization	N/C	[44]
Cs/Gel/PDA‐HA/PDA‐BaTiO_3_ hydrogels	N/C	Output voltage = 0.6V	Inducing macrophage polarization to M2, enhancing angiogenesis and osteogenesis	Lack of validation of the mechanism	[236]
PMMA/PEI/PVDF	Mechanical stress	Output voltage≈150 mV	Providing a similar viscoelastic and piezoelectric microenvironment	Lack of description of biodegradability	[262]
BaTiO_3_ coated porous Ti6Al4V scaffold	Low dose ultrasound	N/C	Enhancing osteogenesis and osseointegration	Evaluation of piezoelectricity was insufficient	[272]
BaTiO_3_/Ti6Al4V	Polarization	d_33_ = 0.89 pC N^−1^	Enhancements in macrophage M2 polarization and bone regeneration	Surface potential stability after in vivo implantation was lacking	[281]
CS/PHB@ZnO	Controllable ultrasonic stimulation	Output voltage = 800 mV	Promoting bone formation by activating CaM/CaN/NFAT signal pathway.	Lack of description of biodegradability	[172]
Ag‐TMSPM‐pBaTiO_3_	Ultrasound	d_33_ = 0.9 pC N^−1^	Promoting osteogenic differentiation	Lack of validation of the mechanism	[358]
Gel‐PD‐CMBT	Polarization	N/C	Reconstruction of electroactive microenvironment	Evaluation of piezoelectricity was insufficient	[310]
3D printed whitlockite	N/C	N/C	Facilitating neuro‐vascularized bone regeneration	Evaluation of piezoelectricity was insufficient	[43]
AuHp@rGO/ calcium sulfate	N/C	Output voltage≈100 mV	Antimicrobial, osteoinductive, and osteoconductive	Lack of validation of the mechanism	[30b]
CoFe_2_O_4_/ P(VDF‐TrFE)	Magnetic field	Surface potential = 91.15 mV	Mimicking the magnetoelectric microenvironment for osteogenesis	Lack of description of biodegradability	[212]
CoFe_2_O_4_@BaTiO_3_/ P(VDF‐TrFE)	Magnetic field	Surface potential = −70 mV	Promoting bone regeneration under inflammatory conditions	Lack of description of biodegradability	[215a]
WP‐TENG	Triboelectric stimulation	Output current≈86 µA	Rejuvenating aged BMSCs	The stability of output current in vivo was not described	[356]
HTP‐NG	Mechanical stress	Output voltage = 35 V	Enhancing calcium ion import and osteogenic differentiation	N/C	[357]

#### 5.1.1 Structural Optimization and Element Doping Strategies

##### Structural Optimization for Enhanced Piezoelectricity

**Figure 6:**
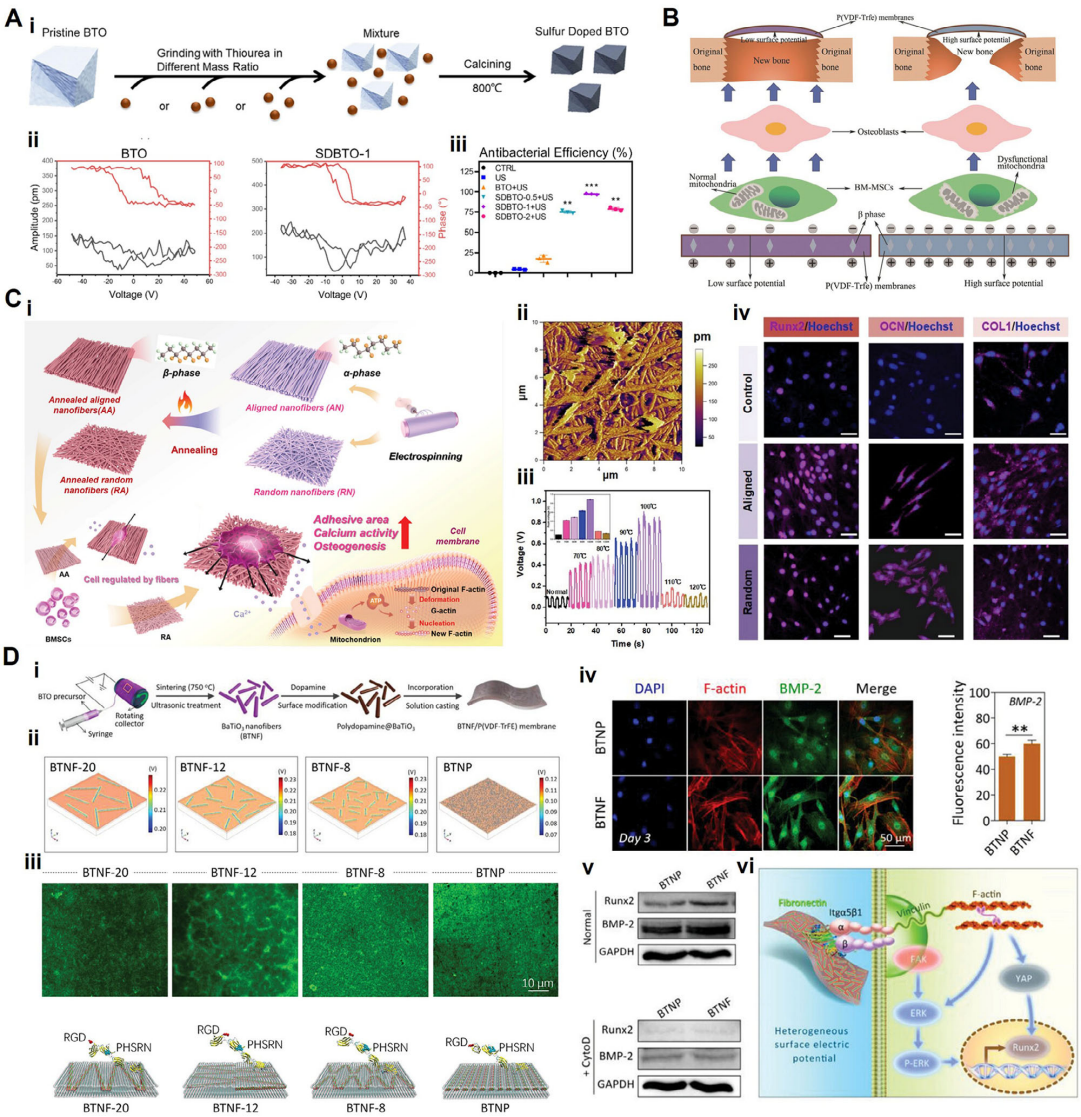
Structural optimization strategies for promoting bone regeneration. A) Ultrasound‐responsive sulfur‐doped BaTiO_3_ for infected bone repair. A‐i. Schematic illustration of the procedure to synthesis sulfur‐doped BaTiO_3_ nanoparticles. A‐ii. The PFM amplitude and hysteresis loop plot of BaTiO_3_ and sulfur‐doped BaTiO_3_ nanoparticles. A‐iii. Antibacterial efficiency. Reproduced with permission.^[^ ^45^ ^]^ Copyright 2022, Elsevier. B) Optimizing β content of P(VDF‐TrFE) for promoting bone regeneration via modulating its surface potential. Reproduced with permission.^[^ ^53^ ^]^ Copyright 2018, Wiley‐VCH. C) The orientation of PVDF affected stem cells differentiation. C‐i. Schematic illustration about preparing progress of annealed align and random PVDF fibers and differentiated mechanisms of BMSCs on fibers. C‐ii. The PFM amplitude of PVDF fibers. C‐iii. Output voltage and peak voltages of the random nanofibers under an external force of 0.5N and different annealing temperatures. C‐iv. Immunofluorescent staining of Runx2, OCN, and COL‐1 of BMSCs cultured on tissue culture dishes, aligned and random PVDF fibers after 7 days of incubation. (Scale bar: 50 µm.) Reproduced with permission.^[^ ^21^ ^]^ Copyright 2023, Wiley‐VCH. D) Surface electric potential optimization for enhanced osteogenesis. D‐i. A diagram of preparation process. D‐ii. The theoretical model of heterogenous surface potential. D‐iii. Heterogeneous surface potential distribution actuates fibronectin (FN) adsorption. D‐iv. The immunofluorescence staining images of BMP‐2. D‐v. Runx2 and BMP‐2 protein expression. D‐vi. The corresponding molecular mechanism. Reproduced with permission. ^[^ ^187a^ ^]^ Copyright 2023, Wiley‐VCH.

Bacterial infection is one of the common complications during the healing process of bone defects, which can impede bone regeneration and potentially lead to secondary bone loss.^[^ ^298^ ^]^ Consequently, the design of bone tissue engineering scaffolds should prioritize anti‐infection and anti‐inflammatory outcomes. The development of nanomaterials responsive to external stimuli, such as ultrasound (US), has witnessed explosive growth.^[^ ^299^ ^]^ Notably, US‐responsive nanomaterials can generate ROS during electron‐hole pair separation under US stimulation.^[^ ^300^ ^]^ Elevated ROS levels play a crucial role in antibacterial therapy.^[^ ^301^ ^]^ Piezoelectric ceramics, exemplified by BaTiO_3_ and ZnO, also exhibit the capability to produce ROS under US irradiation.^[^ ^116^, ^302^ ^]^ Recent research has integrated bone regeneration and bacterial elimination by constructing sulfur‐doped oxygen‐defect BaTiO_3_ for the repair of *Staphylococcus aureus (S. aureus)*‐infected rat tibia bone defects (**Figure** **6**A).^[^ ^45^ ^]^ Under 1.5 W cm^−^^2^ US irradiation, these sulfur‐doped BaTiO_3_ not only enhance osteogenic differentiation by upregulating the TNF‐β signaling pathway but also eliminate S. aureus by generating ROS during electron‐hole pair separation. Moreover, sulfur‐doped BaTiO_3_ significantly increases the piezoelectric properties of BaTiO_3_, with the piezoelectric coefficient (d_33_) rising from 4.78 pm V^−1^ for BaTiO_3_ to 13.95 pm V^−1^ for sulfur‐doped BaTiO_3_, potentially resulting in enhanced osteogenic differentiation.

The piezoelectric biomaterials could provide localized external stimuli in response to deformations.^[^ ^89^ ^]^ The repair of bone defects is a well‐orchestrated physical progress that requires a certain range of certain surface potential adapted to the electric microenvironment of bone defects.^[^ ^303^ ^]^ However, the relationship about surface potential of piezoelectric biomaterials and osteogenic differentiation has not been fully elucidated yet. In 2018, Zhang and coworkers fabricated P(VDF‐TrFE) via annealing treatment and demonstrated that d_33_ = 10 pC N^−1^ (surface potential = ‐53mV) membranes are more suitable for osteogenesis and energy metabolism than those membranes of d_33_ = 20 pC N^−1^ (surface potential = ‐78mV) (Figure 6B).^[^ ^53^ ^]^ Through controlling the annealing treatment from 90 to 120 °C, they discovered that more β phase in P(VDF‐TrFE) scaffolds were formed because of the higher temperature. In addition to the annealing temperature, researchers also found that the positioning of PVDF nanofibers is also crucial for osteogenic differentiation of BMSCs (Figure 6C).^[^ ^21^ ^]^ The aligned and random annealed PVDF possessed more β phases (86.45% and 74.14%, respectively) than polarized pure PVDF. By seeding BMSCs on the different orientated PVDF membranes, they found that BMSCs on random annealed PVDF membranes had more adhesion area and osteogenic potential without external stimulation. This study provided novel perspective for next‐generation piezoelectric scaffolds for bone repair.

The d_33_ value of BaTiO_3_ thin film varies from 14.3 to 54.0 pC/V when the crystal orientation is altered.^[^ ^304^ ^]^ Recent studies indicate that BaTiO_3_ nanofibers (BTNFs) induce a sustained electric field compared to BaTiO_3_ nanoparticles (BTNPs) because of the vastly larger surface acreage of BTNFs. Bai et al.^[^ ^187a^ ^]^ incorporated the BTNFs into P(VDF‐TrFE) and optimized the aspect ratio of BTNFs (Figure 6D). They observed that the anisotropy of the surface potential of BTNFs incorporated membrane can be altered via the spontaneous polarization of BTNFs, resulting in fibronectin aggregation into a network that imitates the topology of the ECM. This study demonstrates that the shape and aspect ratio of BaTiO_3_ can affect the differentiation fate of stem cells through changing the potential distribution on film surface, offering a novel strategy for tissue regeneration.

##### Cooperative Promotion of Osteogenesis via Bioactive/Electroactive Ions and Piezoelectric Stimulation

The inherent conductivity and piezoelectricity of bone play a big part in bone healing.^[^ ^133^ ^]^ When bone defects form due to trauma or diseases, the conductivity and piezoelectricity of the bone microenvironment are significantly impaired, impeding bone regeneration.^[^ ^43^ ^]^ Thus, biomaterials possessing natural or enhanced conductivity and piezoelectricity are of great significance for clinical bone repair.^[^ ^305^ ^]^ As mentioned earlier, piezoelectric ceramics like BaTiO_3_ can enhance the influx of Ca^2+^ to accelerate osteogenesis under mechanical force or polarization.^[^ ^306^ ^]^ However, the single piezoelectric effect of BaTiO_3_ is insufficient to meet the diverse needs of bone repair.^[^ ^307^ ^]^ Research findings indicate that incorporating conductive materials into the design of piezoelectric composites can significantly enhance their piezoelectric output performance by establishing efficient charge transport pathways.^[^ ^308^ ^]^ This breakthrough beyond the limitations of traditional piezoelectric biomaterials holds substantial potential in the field of tissue regeneration, like bone repair.

Recent studies have made multiple attempts to enhance the osteogenic potential of BaTiO_3_ by incorporating it into conductive or piezoelectric polymers in the form of piezoelectric fillers or by adding osteogenic and bioactive ions such as Ca^2+^ and Mn^4+^ to BaTiO_3_.^[^ ^126^, ^309^ ^]^ Recently, researchers have combined the above strategies to construct conductive and piezoelectric composite cryogels aimed at restoring the electroactive microenvironment of bone defects.^[^ ^310^ ^]^ The authors chose cryogel scaffolds for in vivo implantation due to their interconnected porous structures with excellent mechanical strength and rapid shape recovery.^[^ ^311^ ^]^ First, Ca^2+^ and Mn^4+^ doped BaTiO_3_ nanofibers (CMBT) were prepared. The conductive Poly (3,4‐ethylene dioxythiophene)/polystyrene sulfonate (PEDOT: PSS) and CMBT were incorporated into gelatin (Gel) via stirring and ultrasonication. The CMBT‐PD‐Gel composite was obtained after freeze‐drying. This type of combined conductive and piezoelectric cryogel scaffold significantly enhanced osteogenic differentiation.

The piezoelectricity of natural bone has resulted from the polarization of collagen fibers, which undergo misalignment under shear forces, playing a decisive part in the differentiation of BMSCs.^[^ ^312^ ^]^ The osteogenic differentiation is activated by the Ca^2+^ influx when voltage‐gated Ca^2+^ channels (VGCCs) are opened by electrical stimulation, and the cell metabolism of BMSCs and their adenosine triphosphate (ATP) production would be significantly increased.^[^ ^252^ ^]^ Apart from inducing the cell behaviors of BMSCs, electrical stimulation could also inhibit bacterial activity.^[^ ^313^ ^]^ Conventional electrical stimulation realized on the implanted or wearable piezoelectric materials, which could generate electrical potential to regulate cell behavior under mechanical force or external stimulations.^[^ ^214^, ^314^ ^]^ However, heterogeneous interfaces of piezoelectric materials are vary from natural bone, which impede the cell recruitment and differentiation. Thus, the development of implanted piezoelectric materials should consider the chemical composition and surface topology.^[^ ^315^ ^]^ Moreover, the electrical potential generating is highly dependent on external stimulation like ultrasonic waves. Although some piezoelectric films could generate surface potential under deformation induced by cell attachment, such electric signal is not sufficient to satisfy the need of bone repair because of the weak traction force of cells.^[^ ^304^ ^]^

#### 5.1.2 Piezoelectric Effect‐Mediated Vascularized and Neutralized Bone Formation

HAp, alongside collagen, exhibits piezoelectric properties.^[^ ^316^ ^]^ Calcium sulfate (CS), a traditional artificial augmentation material, has attracted considerable interest due to its similarity to bone mineral, facilitating the repair of bone defects in minor surgeries, particularly irregular bone defects.^[^ ^317^ ^]^ However, CS also has some drawbacks in clinical applications due to its rapid absorption and limited mechanical stability.^[^ ^318^ ^]^ Herein, researchers have made significant efforts to enhance the mechanical properties and improve osteogenic activity, such as through surface modification with conductive nanoparticles or piezoelectric nanocomposites.^[^ ^319^ ^]^ For example, to impart to CS piezoelectric properties, enhanced mechanical strength, and antibacterial activity, Chopra et al.^[^ ^30b^ ^]^ synthesized gold nanodots and nanohydroxyapatite on reduced graphene oxide sheets (AuHp@rGO) through a one‐pot hydrothermal reaction, and further functionalized them with antibacterial vancomycin. The acquired nanocomposites were incorporated into CS to create injectable CS bone cements. Experiments have shown that this composite CS had piezoelectric, osteogenic, antibacterial, and pro‐angiogenesis properties.

It is generally acknowledged that the collagen and HAp enable the bone with piezoelectricity (d_33_ = 0.7–2.3 pC N^−1^)^[^ ^320^ ^]^ The whitlockite, derived from HAp doped with rich Mg^2+^, displaying significant polarization because of the domain switch above the phase transition temperature, which endows it similar piezoelectricity with natural bone to promote bone repair.^[^ ^321^ ^]^ Apart from natural slowly degradable HAp with released Ca and P ions, the WH could provide extra Mg^2+^ during the degradation, which could enhance angiogenesis and osteogenesis.^[^ ^322^ ^]^ Wang and coworkers utilized a melt extrusion 3D printing technology to construct the piezoelectric WH (PWH)‐contained composite scaffolds for neuro‐vascularized bone regeneration.^[^ ^43^ ^]^ The PWH composite scaffolds took advantage of bioactive ions and the piezoelectricity in modulating neuron differentiation, angiogenesis, and osteoclasts inhibition. However, the piezoelectricity of PWH and PWH composite scaffolds was not mentioned in detail.

#### 5.1.3 Mechano‐Electric Conversion Strategy for Immunomodulation

In addition to mimicking the heterogeneous potential of bone ECM, precisely modulating cell behavior within the bone microenvironment is still a significant obstacle in developing piezoelectric implants for bone repair. With an aging population and changing lifestyle habits, the global prevalence of diabetes mellitus (DM) has increased dramatically.^[^ ^323^ ^]^ Bone regeneration in patients with diabetes mellitus (DM) is more challenging because the healing process of bone defects is often accompanied by excessive inflammatory responses, leading to delayed or non‐healing of the defects.^[^ ^324^ ^]^ Pro‐inflammatory M1 macrophages accumulate in early healing progress of DM bone defect, recruiting BMSCs via secreting CCL2, CXCL8, and SDF‐1.^[^ ^278c^ ^]^ While anti‐inflammatory M2 macrophages are more important in the late stage, specifically in regenerative process, by promoting osteogenesis, reshaping ECM structure, and facilitating mineralization.^[^ ^325^ ^]^ The retarded or non‐healing of bone defects in DM patients is primarily due to the overactivity of M1 macrophages and their failure to transition to M2 macrophages.^[^ ^326^ ^]^

**Figure 7:**
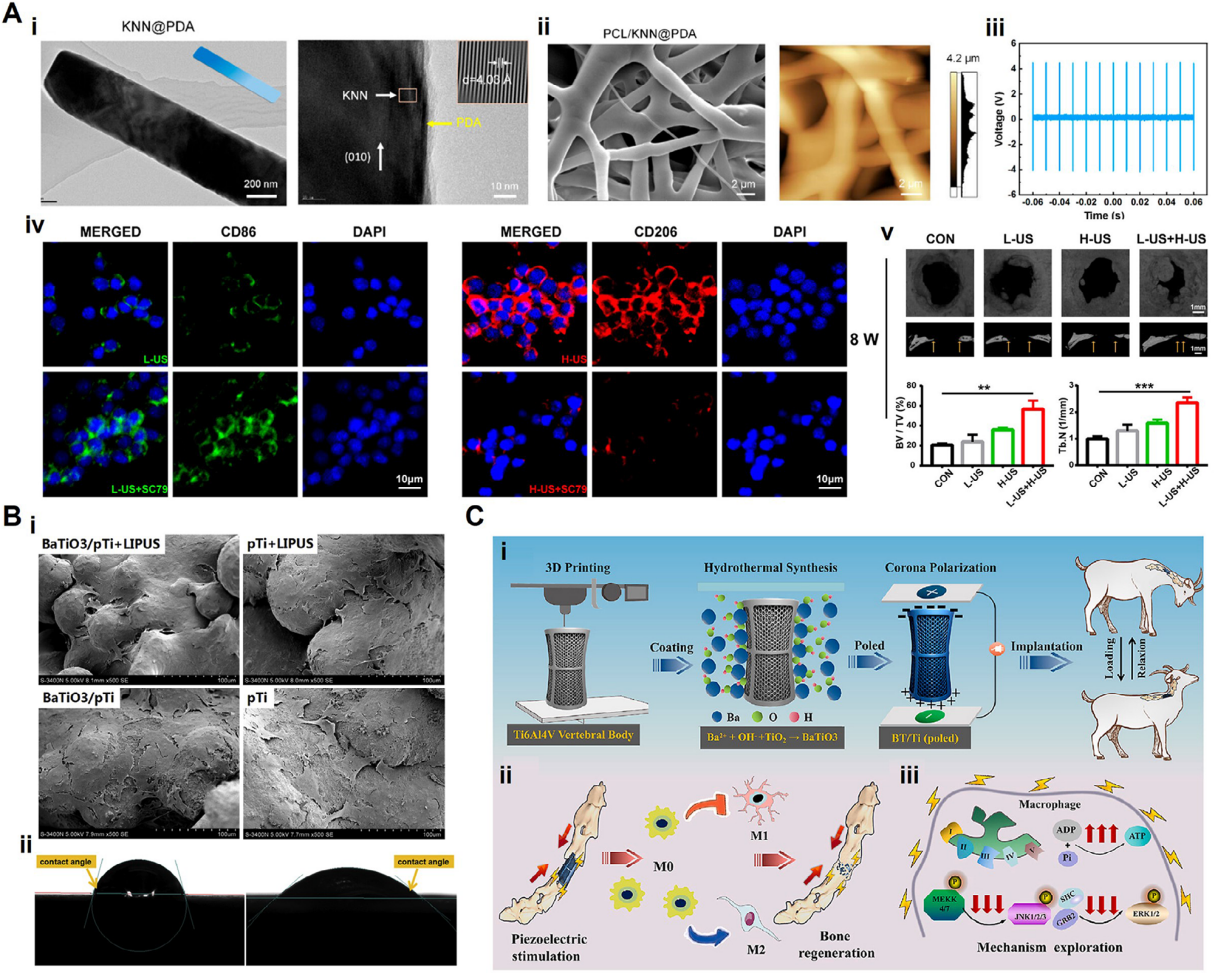
Mechano‐electric conversion strategy for immunomodulation. A) An ultrasound‐activated piezoelectrical membrane achieves optimized diabetic bone regeneration. A‐i. Transmission electron microscopy images of the KNN@PDA. A‐ii. SEM and AFM images show the morphologies of PCL/KNN@PDA film. A‐iii. Open‐circuit voltage of PCL/KNN@PDA film under ultrasound activation. A‐iv. Representative immunofluorescence images of CD86 and CD206 in macrophages treated with or without AKTs inhibitor. (Scale bar: 10 *µm*.) A‐v. Representative micro‐CT images of cranial defect samples at week 8 after implantation, as well as corresponding quantitative analysis of new bone formation by values of BV/TV and Tb.N. (Scale bar: 1 mm.) Reproduced with permission.^[^ ^44^ ^]^ Copyright 2023, American Chemical Society. B) BaTiO_3_‐coated titanium scaffold enhanced osteogenic differentiation for large segmental bone defects using low‐intensity pulsed ultrasound. B‐i. SEM images of BMSCs morphology in different groups after incubation for 4 days. B‐ii. Water contact angle measurement. Reproduced with permission.^[^ ^272^ ^]^ Copyright 2020, Elsevier. C) BaTiO_3_‐coated Ti6Al4V promoted the polarization of macrophages into anti‐inflammatory subtype and bone regeneration. C‐i. The synthetic procedure. C‐ii. Piezoelectric implant promoted M2 polarization and bone regeneration. C‐iii. The mechanisms about how piezoelectric stimulation promoted bone regeneration. Reproduced with permission.^[^ ^281^ ^]^ Copyright 2023, Elsevier.

Therefore, it is crucial for researchers to consider immunomodulation when designing scaffolds for DM bone defects healing. Recently, electroactive materials are proposed to have potential in modulating macrophage polarization.^[^ ^212^ ^]^ Sun and coworkers developed a composite piezoelectric scaffold capable of temporal immunomodulation through varying doses of ultrasonic stimulation during diabetic bone regeneration (**Figure** **7**A).^[^ ^44^ ^]^ First, KNN nanowires were synthesized through a molten‐salt reaction, followed by PDA coating on their surface to prevent aggregation within the PCL matrix. The composite piezoelectric PCL/KNN/PDA scaffolds were then produced via electrospinning. Interestingly, the researchers found that low‐dose ultrasound (US) irradiation downregulated the significant gene expression of M1 macrophages without upregulating M2 macrophage gene expression, which only occurred with high‐dose US irradiation. With programmed external stimulation, these piezoelectric composite scaffolds remarkably facilitated the healing of critical‐sized bone injuries in diabetic rats. This study highlights the potential of piezoelectric biomaterials to modulate immune cells behaviors in bone healing through wirelessly controlled US stimulation.

The healing of bone injuries remains challenging because of uncontrollable inflammation, excessive ROS production, and the inhibition of osteogenesis and angiogenesis.^[^ ^268^ ^]^ Electrical stimulation holds great potential in bone repair by regulating macrophage behavior, cytokine production, and osteocyte metabolism.^[^ ^129^, ^327^ ^]^ New bone also needs an enhanced vascularization, and electrical stimulation contributes to remedying angiogenic function in bone defects. Srirussamee and coworkers discovered that direct current (DC) did not enhance vascular endothelial growth factor (VEGF) expression,^[^ ^271a^ ^]^ while Kim and coworkers found that alternating current (AC) could noticeably enhance the expression of VEGF.^[^ ^271b^ ^]^ Inspired by these discoveries, Wu et al.^[^ ^236^ ^]^ constructed bone tissue engineering piezoelectric scaffolds that combined immunomodulation of the bone microenvironment with AC generation under mechanical loading. The integration of BaTiO_3_ significantly promoted the mechanical abilities of piezoelectric hydrogel and enhanced osteogenic activity at the same time. Moreover, the piezoelectric hydrogel significantly modulated M2 macrophage polarization, and reconstructed vascularization.

The physical, well‐coordinated processes involved in bone repair include early immune response, cell recruiting, vascular network formation, osteogenic differentiation and mineralization.^[^ ^328^ ^]^ As a fibrous and vascularized membrane, periosteum contributes a lot to osteogenesis and chondrogenesis during early stages of repair after autograft.^[^ ^276^, ^329^ ^]^ The ideal biomimetic periosteum materials should participate in these biological events and facilitate bone regeneration and healing. Previous studies have shown that PDA‐functionalized materials are potential candidates for bone repair, capable of modulating immune response and promoting cell adhesion and proliferation.^[^ ^330^ ^]^ In 2023, Liu and coworkers combined PDA functionalization and piezoelectric materials by adding PDA‐modified HAp and BaTiO_3_ to PHBV solution and constructed composite periosteum membranes using a spin‐coating method.^[^ ^277^ ^]^ The combination of PDA‐modified HAp and BaTiO_3_ significantly enhanced the surface hydrophilicity and roughness of the PHBV membrane. The composite periosteum membranes demonstrated osteogenic and immunomodulatory functions both in vitro and in vivo, accelerating bone healing in rats with critical‐sized cranial defects.

In clinical practice, addressing large bone defects that exceed the critical size remains a formidable challenge, as the defect site often fails to achieve complete bone regeneration.^[^ ^331^ ^]^ Ideal bone engineering scaffolds for large bone defects healing should prioritize key factors such as robust mechanical abilities to support locomotion, and the capacity to accelerate angiogenesis and osteogenic differentiation.^[^ ^332^ ^]^ Porous titanium (pTi) alloy scaffolds are potential candidates for regenerating large segmental bone defects because of satisfied mechanical strength and osseointegration potential.^[^ ^332^, ^333^ ^]^ However, despite these advantages, the low biological activity and lack of a piezoelectric effect in pTi hinder its ability to support effective bone regeneration.^[^ ^334^ ^]^ BaTiO_3_ is frequently selected as a bone engineering material capable of mimicking the stress‐generated potential (SGP) observed in natural bone.^[^ ^335^ ^]^ Previous researches have indicated that BaTiO_3_ can be coated onto the surface of pTi using a wet chemical strategy for repairing limited‐sized bone defects. To address larger bone defects, external mechanical stimulation, such as mechanical waves, is required to activate the piezoelectric effect of BaTiO_3_. Fan and colleagues deposited BaTiO_3_ on the surface of Ti6Al4V in order to create a piezoelectric Ti scaffold. Under LIPUS, the piezoelectric implant significantly enhanced osteogenic differentiation and osseointegration at week 6 and 12 after implanting in rabbits with large segmental defects (Figure 7B).^[^ ^272^ ^]^ In addition, Wu and coworkers have found that piezoelectric stimulation resulted from poled BaTiO_3_ nanoparticles on Ti6Al4V scaffolds effectively promoted macrophage polarization toward anti‐inflammatory subtypes via curbing MAPK/JNK pathway, activating OXPHOS, and enhancing bone repair of cervical vertebral defect in sheep (Figure 7C).^[^ ^281^ ^]^ Herein, coating piezoelectric nanoparticles on bone implants has enhanced bone regeneration outcomes, thereby broadening the application scenarios.

#### 5.1.4 Mechanotransduction Strategy for Biomimetic Piezoelectric Periosteum

Limited‐size bone defects resulted from trauma or tumors can heal effectively, as the periosteum covering the defect supplies sufficient osteoblasts.^[^ ^336^ ^]^ As for critical‐size bone defects, lack of periosteum impedes bone healing because osteoblasts are difficult to move to distant area of defects.^[^ ^337^ ^]^ The repair of critical‐size bone defects has garnered increasing attention due to advancements in electroactive piezoelectric biomaterials. For instance, piezoelectric polymers such as P(VDF‐TrFE) (PVFT) can stimulate the function of the periosteum, facilitate stem cell adhesion, and enhance osteogenic differentiation.^[^ ^338^ ^]^ The periosteum and bone matrix, comprising cortical and cancellous bone, form the structure of bone. Developing novel piezoelectric scaffolds that mimic the constitution and function of periosteum and bone matrix is crucial for critical‐size bone defects healing.^[^ ^339^ ^]^ Motivated by the natural characteristics of bone tissue, multilateral biomimetic scaffolds were constructed by depositing bioactive glass micro‐nano particles (BGM) onto the negative surface of polarized PVFT.^[^ ^261^ ^]^ This biomimetic nanofibrous and piezoelectric structure of the composite scaffolds can facilitate the formation of bone‐binding surface and accelerate the bone healing by activating the calcium‐sensing receptor of osteoblasts through Ca^2+^ signaling.

Heterogeneity is one of the fundamental characteristics of ECM, originating from the diversity of bone cells and the arrangement of ECM components, and it plays a big part in regulating cell behavior and fate.^[^ ^340^ ^]^ In other words, bone cells, such as BMSCs, are surrounded by anisotropic collagen fibers, and the piezoelectricity generated by their anisotropic structure produces physical electrical signals that influence the stem cells.^[^ ^341^ ^]^ Recent research has revealed that the topological structure and piezoelectric properties of piezoelectric biomaterials can also modulate stem cell behavior.^[^ ^342^ ^]^ To some extent, especially in the physiological activities, the heterogeneity of electricity may represent the electrophysical microenvironment of ECM.^[^ ^343^ ^]^

Therefore, it is crucial to elucidate how piezoelectric biomaterials, with their heterogeneous distribution of electric charge, modulate cell behavior and fate at cellular and molecular levels. As we mentioned before, Bai et al.^[^ ^187a^ ^]^ have found that the heterogeneous potential of piezoelectric membranes could be modified via adjusting the integrated BTNF aspect ratios. In vitro results indicated that heterogeneous membrane potential resulted in the formation of a meshwork pattern of fibronectin, which subsequently enhanced cell adhesion and proliferation. The composite membranes were implanted in mature rats with 8 mm critical‐sized calvarial defects, and as a result, bone regeneration was significantly improved due to the heterogeneous surface electric potential.

In addition to the natural piezoelectric effect of collagen fibers in ECM, viscoelastic properties are also important in modulating BMSCs behavior through mechanotransduction between cells and the matrix.^[^ ^344^ ^]^ Researchers have demonstrated that BMSCs can sense and adapt to nanoscale vibrations, leading to their differentiation into mineralized bone, while mechanical force, or movements can initiate the expression of signaling pathways like Wnt, TRPV4, and Rho.^[^ ^345^ ^]^ These biological cues provide valuable insights for designing artificial piezoelectric scaffolds that incorporate the viscoelastic and piezoelectric abilities of ECM. Zhang et al.^[^ ^262^ ^]^ developed porous poly(methylmethacrylate) (PMMA)/polyethyleneimine (PEI)/PVDF composites that exhibit piezoelectric and viscoelastic properties close to those of the bone microenvironment. PMMA and PVDF were combined with PEI through strong hydrogen bonding, leading to the formation of entangled conformations among the three segments. This composite exhibited viscoelastic properties similar to those of the bone microenvironment and converted mechanical force into electric fields during locomotion. These composite bionic implants significantly upregulated osteogenic genes in human BMSCs and triggered the expression of Piezo 1 proteins via mechanotransduction signaling pathways.

#### 5.1.5 Smart and Personalized Strategy for Precise Bone Regeneration

##### Smart 3D Printing Piezoelectric Scaffolds

To address bone defects, restore locomotion, and protect internal organs, autogenous bone grafting is a common strategy, albeit with prolonged healing times and chronic pain.^[^ ^346^ ^]^ However, autogenous transplantation alone cannot meet clinical demand, necessitating the urgent advancements of bone engineering materials which are capable of mimicking the bone microenvironment and expediting the bone repair process.^[^ ^332^ ^]^ As discussed earlier, the ECM of bone comprises piezoelectric collagen fibers and non‐collagen components.^[^ ^63^ ^]^ Among these, the parallel interspersed collagen fibers confer bone tissue with an endogenous electrical field, facilitating the osteogenic differentiation of BMSCs.^[^ ^59^ ^]^ Therefore, constructing a 3D porous ECM‐like scaffold to revitalize the endogenous electrical field of bone tissue represents a promising approach to expedite bone tissue regeneration.^[^ ^64^ ^]^ Integrating piezoelectric ceramics and polymers into 3D ECM‐like scaffolds can imbue the materials with the ability to produce electrical stimulation under mechanical force, thereby modulating cell behavior within the bone tissue microenvironment.^[^ ^53^ ^]^

Regarding natural piezoelectric polymers, PHB emerges as a promising substitute for bone repair owing to exceptional biocompatibility and biodegradability. However, the limited piezoelectric properties of PHB prove insufficient for restoring the electrical field necessary for bone healing.^[^ ^347^ ^]^ To enhance both mechanical properties and the capability to generate an electrical field, ZnO nanoparticles are incorporated.^[^ ^348^ ^]^ For instance, Chen and coworkers designed a 3D nanofiber/aerogel composite by incorporating 2% ZnO into PHB through electrospinning. The resulting composite nanofibers were then integrated with chitosan, and the mixture was frozen into 3D porous scaffolds via lyophilization.^[^ ^348^ ^]^ Subjected to controllable ultrasonic stimulation (0.5 W/cm^2^), the 3D porous ECM‐like nanofiber‐aerogel significantly restored the local electric microenvironment and promoted osteogenesis.

**Figure 8:**
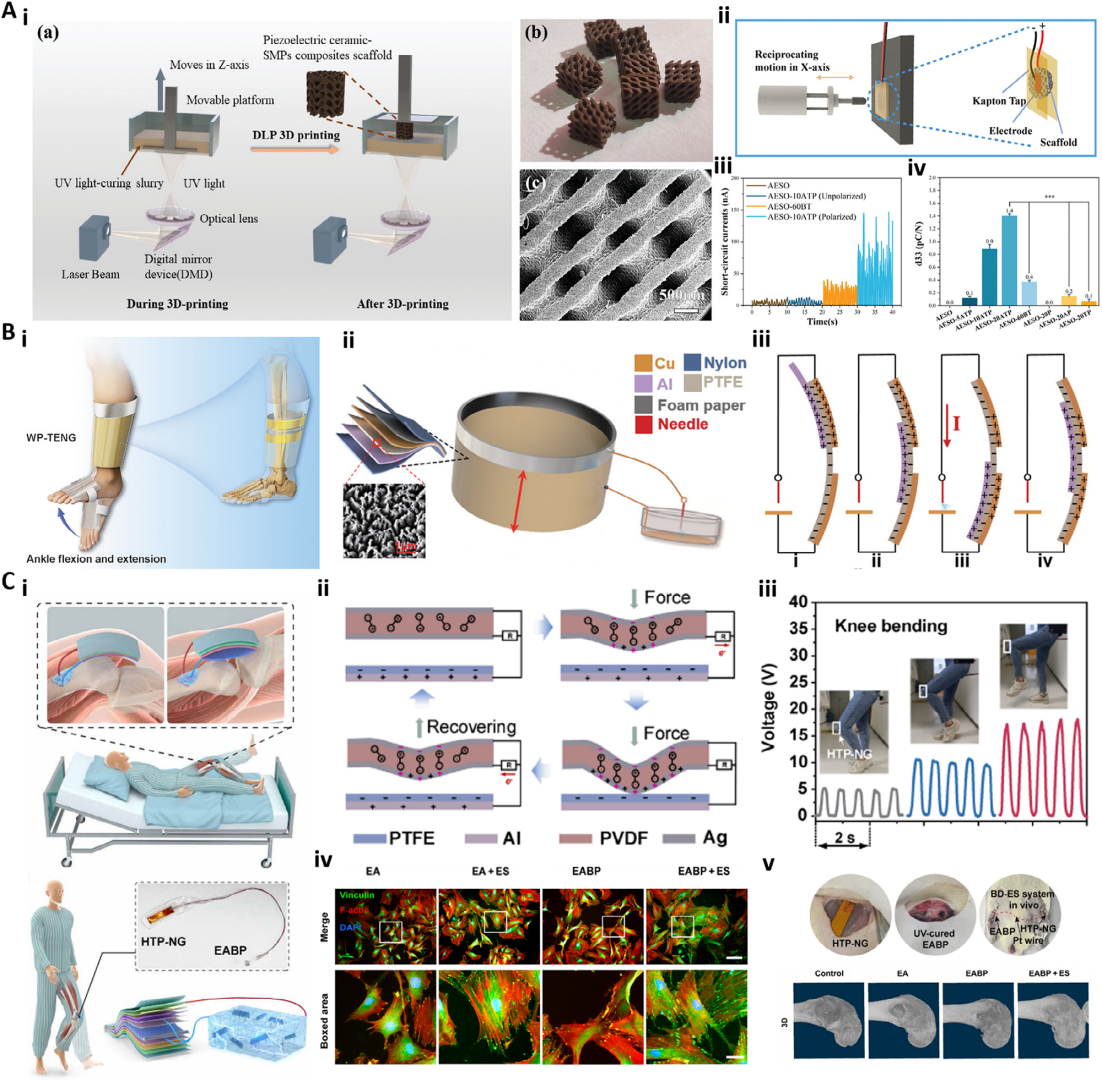
Smart and personalized strategy for precise bone regeneration. A) A 3D shaped piezoelectric scaffold for bone repair. A‐i. The prepared process, macroscopic and SEM picture of 3D shaped piezoelectric scaffolds. A‐ii. Reciprocating mechanical stimulation device. A‐iii. Short‐circuit currents. A‐iv. Piezoelectric constant (d_33_) of different scaffolds. Reproduced with permission.^[^ ^358^ ^]^ Copyright 2023, Wiley‐VCH. B) Wearable pulsed triboelectric nanogenerator for bone repair. B‐i. The schematic illustration of wearable pulsed triboelectric nanogenerator (WP‐TENG) stimulated by movement. B‐ii. The schematic picture of WP‐TENG. B‐iii. Electricity generation process. Reproduced with permission.^[^ ^356^ ^]^ Copyright 2022, Wiley‐VCH. C) Exercise‐activated electrical stimulation platform for accelerated bone repair. C‐i. Illustration of the BD‐ES system. C‐ii. Schematic depicting the operational principle of HTP‐NG. C‐iii. Output voltage of HTP‐NG under knee bending. C‐iv. Immunofluorescence images of F‐actin and vinculin. Scale bars, 200 and 40 µm. C‐v. 3D reconstruction images of the distal femur by micro‐CT. Reproduced with permission.^[^ ^357^ ^]^ Copyright 2024, American Association for the Advancement of Science.

In the realm of piezoelectric biomaterial development, 3D printing emerges as a compelling approach for fabricating bone repair scaffolds. Leveraging digital light processing (DLP) 3D technology enables fast prototyping with high resolution.^[^ ^349^ ^]^ Among the prevalent piezoelectric biomaterials, BaTiO_3_ nanoparticles possess notable piezoelectric properties (d_33_ = 191 pC N^−1^).^[^ ^350^ ^]^ However, certain drawbacks hinder the widespread application of BaTiO_3_, including poor processability and toughness.^[^ ^351^ ^]^ Combining shape memory polymer (SMP) with BaTiO_3_ nanoparticles via digital light processing printing (DLP) offers a streamlined preparation method, yielding composite scaffolds more conducive to in vivo implantation.^[^ ^352^ ^]^ For instance, Li and colleagues capitalized on the benefits of acrylate epoxidized soybean oil (AESO), which can revert to its primary structure under near‐infrared (NIR) light irradiation, to integrate piezoelectric ceramics like BaTiO_3_ through DLP 3D printing (**Figure** **8**A).^[^ ^327^ ^]^ AESO exhibits excellent biocompatibility and effectively transfers stress to piezoelectric ceramics. Furthermore, incorporating conductive Ag nanoparticles and 3‐(trimethoxysilyl) propyl methacrylate (TMSPM) onto PDA‐coated BaTiO_3_ nanoparticles notably enhances the piezoelectricity of composite scaffolds, thereby promoting bone regeneration in vivo.

##### Personalized and Wearable Piezoelectric Materials

Electrical stimulation is an effective tool to enhance osteogenesis and angiogenesis, limit local inflammation, and promote graft integration via modulating intracellular signaling pathways.^[^ ^353^ ^]^ The triboelectric nanogenerator (TENG) could achieve the output of electrical flow under friction.^[^ ^354^ ^]^ The triboelectric stimulation has a wide range of applicating scenarios, such as achieving bone repair through activating Piezo 1, promoting the influx of Ca^2+^, regulating the expression of HIF‐1α.^[^ ^355^ ^]^ Wang and coworkers have developed a wearable pulsed triboelectric nanogenerator (WP‐TENG) to utilize the triboelectric stimulation driven by human movement (Figure 8B).^[^ ^356^ ^]^ The WP‐PENG could not only rejuvenate the osteogenesis of aged BMSCs via activating the mechanosensitive ion channel Piezo 1, but also enhance tube formation capacity of HUVECs. In clinical applications, designing a flexible and lightweight piezoelectric scaffold for bone defect repair remains a challenge. Recently, a hybrid tribo/piezoelectric hydrogel was specifically developed to enhance bone defect regeneration in response to rehabilitation exercises.^[^ ^357^ ^]^ The piezoelectric PVDF scaffolds were implanted subcutaneously to utilize mechanical stimuli from knee movements, transferring electrical stimulation to the conductive hydrogel in femur defects to enhance osteogenesis (Figure 8C). Nevertheless, due to the poor biodegradability of PVDF, a secondary surgery was required to eliminate the device after healing.

### 5.2 Piezoelectric Biomaterials for Cartilage Regeneration

Osteoarthritis is a degenerative, debilitating disorder characterized by cartilage's deterioration, resulting in knee pain and reduced mobility.^[^ ^264^ ^]^ Normally, pads of cartilage provide cushioning for bones. However, as the cartilage deteriorates with age or wears out, bones begin to rub against each other, and daily activities like walking become extremely painful.^[^ ^360^ ^]^ The best way to treat osteoarthritis at the moment is to replace the damaged cartilage with healthy cartilage from autologous or allogeneic sources.^[^ ^361^ ^]^ There are still many challenges to healthy cartilage transplantation, such as tissue suitability, host immune response after transplantation, and chondrocyte activity, among others.^[^ ^362^ ^]^ To regenerate healthy cartilage, scientists have attempted to induce cartilage regeneration by enhancing the effects of chemical growth factors,^[^ ^363^ ^]^ or using bioengineering materials to provide scaffolds for cartilage growth.^[^ ^364^ ^]^ However, the effect of these two methods to induce cartilage growth remains unsatisfactory. That is, the regrown cartilage does not function as well as natural cartilage, and it breaks under the normal pressure of joint.

Researches have elucidated that piezoelectricity is one of the key factors in achieving cartilage repair.^[^ ^42^ ^]^ Electrical polarization occurs when cartilage is deformed under mechanical force.^[^ ^7b^ ^]^ The electrical polarization of ECM under mechanical loads can affect the membrane receptors of chondrocytes and eventually transmit the signal into the nucleus to alter cellular behaviors.^[^ ^290^ ^]^ As the single type of cells in cartilage, chondrocytes are accountable for keeping and renewing cartilage via synthetic and metabolic balance.^[^ ^365^ ^]^ Mechanical signals act a pivotal part in both health and pathology of cartilage, and various mechanisms are involved in the mechanotransduction of chondrocytes.^[^ ^366^ ^]^ Piezo1 and Piezo2 mechanosensitive ion channels are responsible for mechanotransduction of high‐strain mechanical stress in chondrocytes through increased Ca^2+^ influx.^[^ ^367^ ^]^

Efficiently repairing cartilage is difficult due to its non‐vascular and weak metabolic properties. Although cartilage is a piezoelectric tissue, the use of piezoelectricity in cartilage repair remains poorly studied.^[^ ^263^ ^]^ The piezoelectric materials generate electric signals which derived from asymmetric ions shifting under mechanical force or other external stimuli.^[^ ^337^ ^]^ The electrical stimulation then prompts the calcium ion channels’ opening and ultimately promotes the translation of growth factors such as TGFβ and BMP2, and transcription of several proteins that are propitious to the ECM production.^[^ ^368^ ^]^ The production of TGFβ facilitates the chondrocytes differentiation in cartilage growth and repair. Taking advantage of piezoelectric characteristics of cartilage and the effect of piezoelectric properties on chondrocytes, piezoelectric bioceramics like BaTiO_3_^[^ ^14a^ ^]^ and piezoelectric polymers like PVDF^[^ ^369^ ^]^ and PLLA^[^ ^55^ ^]^ have been developed to construct piezoelectric scaffolds for optimized cartilage regeneration. In this section, we elaborate the design, construction, and mechanisms of piezoelectric scaffolds in cartilage repair.

#### 5.2.1 Piezoelectric Biomaterials for Cartilage Regeneration

The piezoelectric effect of piezoelectric materials is responsible for energy conversion, shifting mechanical energy to electrical energy under mechanical force and vice versa, bridging the mechanical stimulation and electrical polarization.^[^ ^370^ ^]^ Compared to other implanted piezoelectric materials, piezoelectric nanomaterials are able to be stimulated by external stimulation (US irradiation) and generated electrical polarization in situ.^[^ ^371^ ^]^ Moreover, piezoelectric nanomaterials can be mixed with other functional nanomaterials or polymers to construct composite scaffolds for tissue regeneration. However, the application of piezoelectric nanomaterials is seldom been explored in boosting chondrogenesis. Among piezoelectric nanomaterials, BaTiO_3_ possesses high piezoelectric coefficient, well biocompatibility and potential osteogenic inducing activity.^[^ ^372^ ^]^ As we mentioned before, the piezoelectricity of BaTiO_3_ enhance the osteogenesis, and modification of BaTiO_3_ intensify the mechanical properties of composites to a certain extent.^[^ ^373^ ^]^ The composite scaffolds comprised PHBV and 20% BaTiO_3_ fabricated by electrospinning technology demonstrated piezoelectric coefficient similar to natural cartilage (ranged from 0.63 to 1.4 pC N^−1^) and excellent mechanical performance.^[^ ^14a^ ^]^ The piezoelectric scaffolds significantly promoted chondrogenesis and accelerated the gene expression of collagen II.

**Figure 9:**
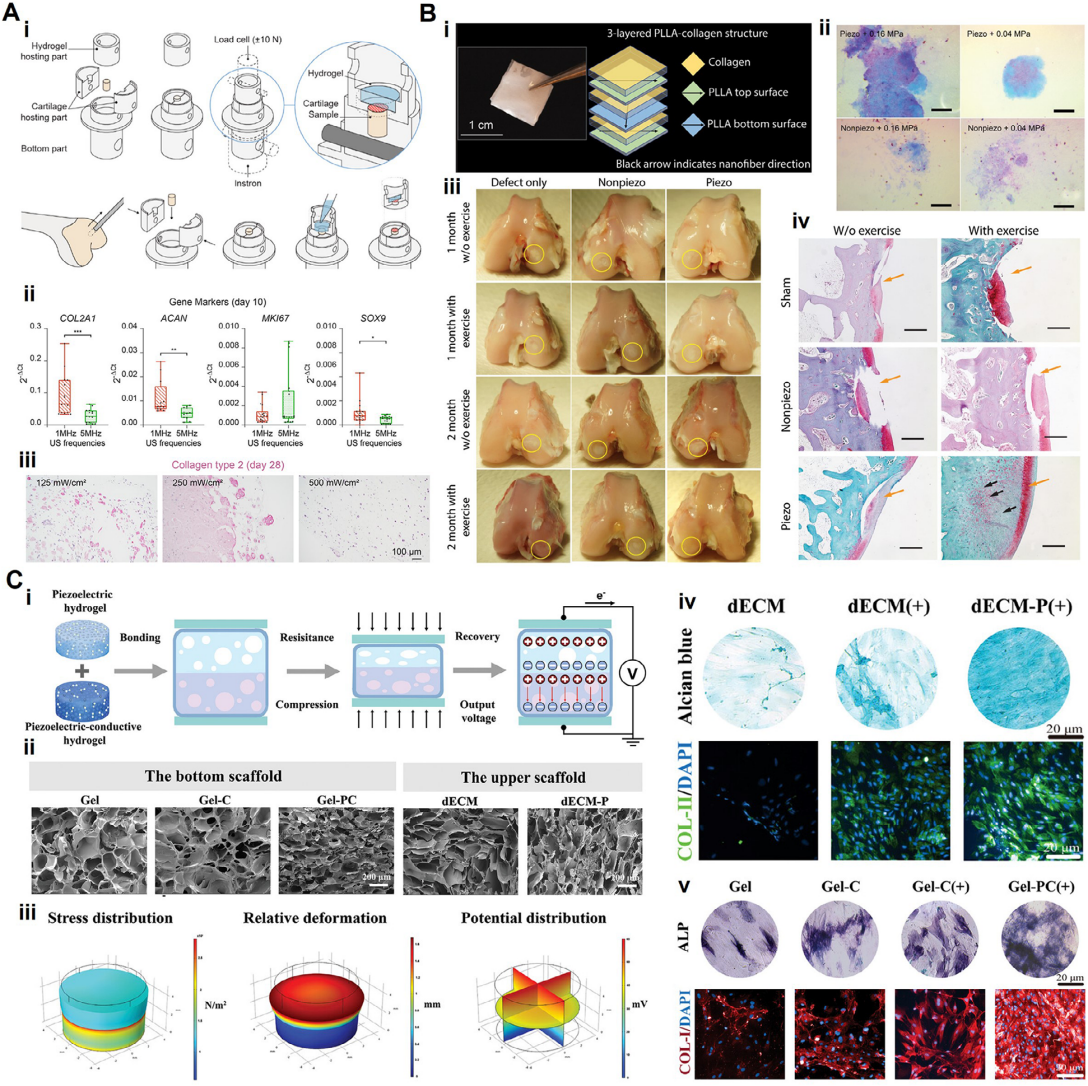
Piezoelectric scaffolds for cartilage and osteochondral defects and repair. A) Ultrasound‐activated piezoelectric nanocomposite hydrogel for chondrogenesis. A‐i. The procedure of adhesion strength evaluation. A‐ii. Expression of *COL2A1*, *ACAN*, *SOX9*, and *MKI67* genes. A‐iii. Immunostaining of collagen type 2 on day 28 under various US frequencies. (Scale bar: 100 *µm*.) Reproduced with permission.^[^ ^375^ ^]^ Copyright 2024, American Chemical Society. B) Exercise‐induced piezoelectrical stimulation for enhanced chondrogenesis. B‐i. The Optical image and schematic illustration of material preparation. B‐ii. Alcian blue staining of GAGs (Scale bars: 200 µm.). B‐iii. Gross images of femurs. Yellow circle indicates the defected area. B‐iv. Safranin O/fast green staining (Scale bars: 500 µm.) Reproduced with permission.^[^ ^55^ ^]^ Copyright 2022, American Association for the Advancement of Science. C) Piezoelectric/Conductive integrated hydrogel for the repair of osteochondral defects. C‐i. Characteristics of piezoelectric‐conductive hydrogel. C‐ii. SEM images of the hydrogel. C‐iii. Distribution of the stress, relative deformation, and electric potential in the D‐P hydrogel calculated from finite element analysis. C‐iv. Alcian blue staining and immunofluorescence images of COL‐II, along with a 2.5D view of immunofluorescence in different groups. C‐v. ALP staining and immunofluorescence images of COL‐I, along with a 2.5D view of immunofluorescence in different groups. Reproduced with permission.^[^ ^224^ ^]^ Copyright 2024, Wiley‐VCH.

To better introduce BaTiO_3_ into the application of cartilage repair, macroscopic materials design should be paid more attention. Recent studies have indicated that LIPUS has potential capabilities of enhancing chondrogenesis.^[^ ^374^ ^]^ Recent research validated the combined application of piezoelectric nanomaterials and ultrasound stimulation in boosting chondrogenesis by embedding piezoelectric BaTiO_3_ nanomaterials and graphene oxide (GO) nanoflakes in hydrogels, activated by dose‐controlled US stimulation (**Figure** **9**A).^[^ ^375^ ^]^ The BaTiO_3_ nanoparticles (BTNPs) were fabricated with a diameter of ≈60 nm (piezoelectric coefficient ≈118.6 pm V^−1^). To avoid BTNPs aggregated in clusters outside the cells, propylene glycol alginate (PGA) was coated on the surface of BTNPs to promote the solution stability and enhancing the incorporating efficiency. The synergic use of US at 1 MHz and 250 mW/cm^2^ significantly enhanced chondrogenesis at day 10 and demonstrated a considerable anti‐inflammatory effect.

Piezoelectric materials can produce electrical stimulation when joints move, thereby promoting cartilage regeneration.^[^ ^376^ ^]^ Normal devise for ES production like direct current or capacitive coupling have risks for infection and pain, limiting electrical stimulation in clinical use.^[^ ^377^ ^]^ Thus, piezoelectric materials combined with physical stress like joint movement could realize the self‐production of ES and accelerate the healing of hyaline cartilage. To introduce piezoelectricity for cartilage regeneration, the PVA/PVDF hydrogel (15:15) with Ag nanowires (0.3%) was constructed to enhance β phase formation of PVDF and inhibit the bacteria formation in contact with chondrogenic layer with the aim of achieving a better outcome of osteochondral defects repair.^[^ ^369^ ^]^ Unfortunately, PVDF are undegradable and not the ideal candidates for cartilage repair. While PLLA are degradable piezoelectric polymers which has an extensive application as erodible implants. Early researches have elucidated that PLLA could promote bone healing under ultrasound irradiation. Inspired by the joints structure and piezoelectrical properties of PLLA, Liu et al.^[^ ^55^ ^]^ further demonstrated that piezoelectric PLLA scaffolds could produce surface potential that was beneficial for chondrogenesis under US or joints load (Figure 9B). The 3D PLLA scaffolds were constructed to constitute a sandwich structure via using rat tail collagen I served as an adhesive layer between PLLA nanofibers. The first layer‐PLLA and third‐layer PLLA were parallel and the medium layer was oriented perpendicularly to the other two layers. The surface of PLLA scaffolds with negative charge possessed better outcome of chondrogenesis compared to surfaces with positive charges, cause negative charges considerably important to the glycosaminoglycans (GAGs) that are crucial to cartilage health. Experiments have revealed that the PLLA scaffolds noticeably promotes cell adhesion and triggered Ca^2+^ influx, leading to the secretion of TGF‐β1 that facilitates chondrogenic differentiation.

PLLA is a biodegradable piezoelectric material that degrades slowly (1‐2 years), and it is suitable to serve as a biocompatible implant to provide long‐term surface potential for cell migration and differentiation.^[^ ^169^, ^378^ ^]^ To implant PLLA scaffolds into joints, invasive surgery may cause damage, inflammation, and infection to other healthy tissue so it is urgent to find a novel strategy to implant the piezoelectric PLLA scaffolds for cartilage regeneration. Hydrogels are potential candidates for tissue implanting due to the porous aqueous structure for cell growth, easily form irregular defects and reform cartilage defects, and non‐invasive injection.^[^ ^379^ ^]^ An injectable piezoelectric hydrogel was introduced to repair the cartilage defects, consisting of short PLLA nanofibers (NFs‐PLLA) and collagen matrix.^[^ ^222^ ^]^ This piezoelectric hydrogel promoted chondrocytes migration and differentiation through the secretion of TGF‐β1, and promoted subchondral bone formation. However, a deep understanding of the mechanisms of how piezoelectricity upregulates the chondrogenic gene expression and the cartilage layer of rabbits and humans are different (rabbits: 250–700 µm; human: 1–3mm). So large animals with similar cartilage thickness to human such as sheep and horses should be further executed to reflect the therapeutic outcomes of piezoelectric hydrogels and ultrasound parameters of clinical use to activate piezoelectric hydrogel need to be reconfirmed cause the different anatomy of human joints.

Although several studies have demonstrated that PLLA could mimic the natural piezoelectricity of cartilage, the application of PLLA in cartilage regeneration has some drawbacks due to its limited piezoelectric coefficient, owing to its relatively high modulus. Recently, researchers have reported that incorporating PHBV into PLLA (the mass ratio is 7:3) has the potential to increase piezoelectric coefficient of PLLA by reducing its modulus.^[^ ^380^ ^]^ This approach was implemented because both of these two polymers have homologous monomers which allowed for miscibility to a certain extent when forming composites due to their analogous chiral carbon atoms.^[^ ^381^ ^]^ The piezoelectric coefficient of the composite was 2.0‐2.5 times more than that of PLLA, which promoted the repair of cartilage defects via calcium and TGF‐β signaling pathways.

#### 5.2.2 Piezoelectric Composites for Osteochondral Defects Repair

Osteochondral defects are prevalent due to sports‐related injuries and an aging population, for which there is no specific treatment, and are particularly common in North America and Europe.^[^ ^382^ ^]^ The repair of both cartilage and bone is challenging due to the differing requirements for the biphasic differentiation of BMSCs. Piezoelectricity in bone and cartilage, mainly originating from the deformation of collagen in ECM, contributes to osteochondral defect healing.^[^ ^383^ ^]^ Researches have proven that the chondrogenesis requires low voltage outputs, whereas the osteogenic differentiation of BMSCs needs higher voltage outputs.^[^ ^55^, ^384^ ^]^ Motivated by this phenomenon, Liu and coworkers constructed a piezoelectric‐conductive composite scaffold, modified with the piezoelectric amino acid phenylalanine, which self‐assembles within a piezoelectric cartilage‐decellularized extracellular matrix (dECM) on the upper layer and a gelatin matrix on the lower layer (Figure 9C).^[^ ^224^ ^]^ As an essential amino acid, the self‐assembling diphenylalanine (FF) forms a hexagonal structure with a d_33_ value of 18 pm V^−1^. Moreover, the integration of conductive poly(3,4‐ethylenedioxythiophene) (PEDOT) enhanced the electrical stimulation in the lower layer, supporting osteogenic differentiation. This piezoelectric‐conductive hydrogel scaffold significantly enhanced both chondrogenic and osteogenic differentiation in vitro and promoted osteochondral defect repair in Parma pig models.

### 5.3 Piezoelectric Biomaterials for Skeletal Muscles Repair

Skeletal muscles form 30–40% of the mass of an adult body and are essential to daily exercise and activities.^[^ ^385^ ^]^ They possess a robust regenerative capacity even at a mature age to produce new muscle cells and ECM, surrounded by re‐established vascular and neural networks.^[^ ^386^ ^]^ However, in the case of severe trauma such as surgery and trauma, it is difficult for muscles to repair completely. Electrical stimulation has significant potential in tissue regeneration, as the endogenous electrical signal produced from the deformation of collagens, transepithelial potential, among other factors, could promote cell migration and differentiation.^[^ ^387^ ^]^ Currently, piezoelectric biomaterials are widely used in bone and cartilage repair, as well as in wound healing, because of the property to produce electrical stimulation under mechanical force or ultrasound, which enhances stem cell migration and promotes tissue repair.^[^ ^388^ ^]^ Due to the reciporal motion of skeletal muscles, piezoelectric scaffolds for tissue engineering must achieve both high piezoelectric performance and excellent tensile strength.^[^ ^389^ ^]^ Unfortunately, balancing high piezoelectricity with a high elastic modulus for effective skeletal muscle repair remains challenging.

**Figure 10:**
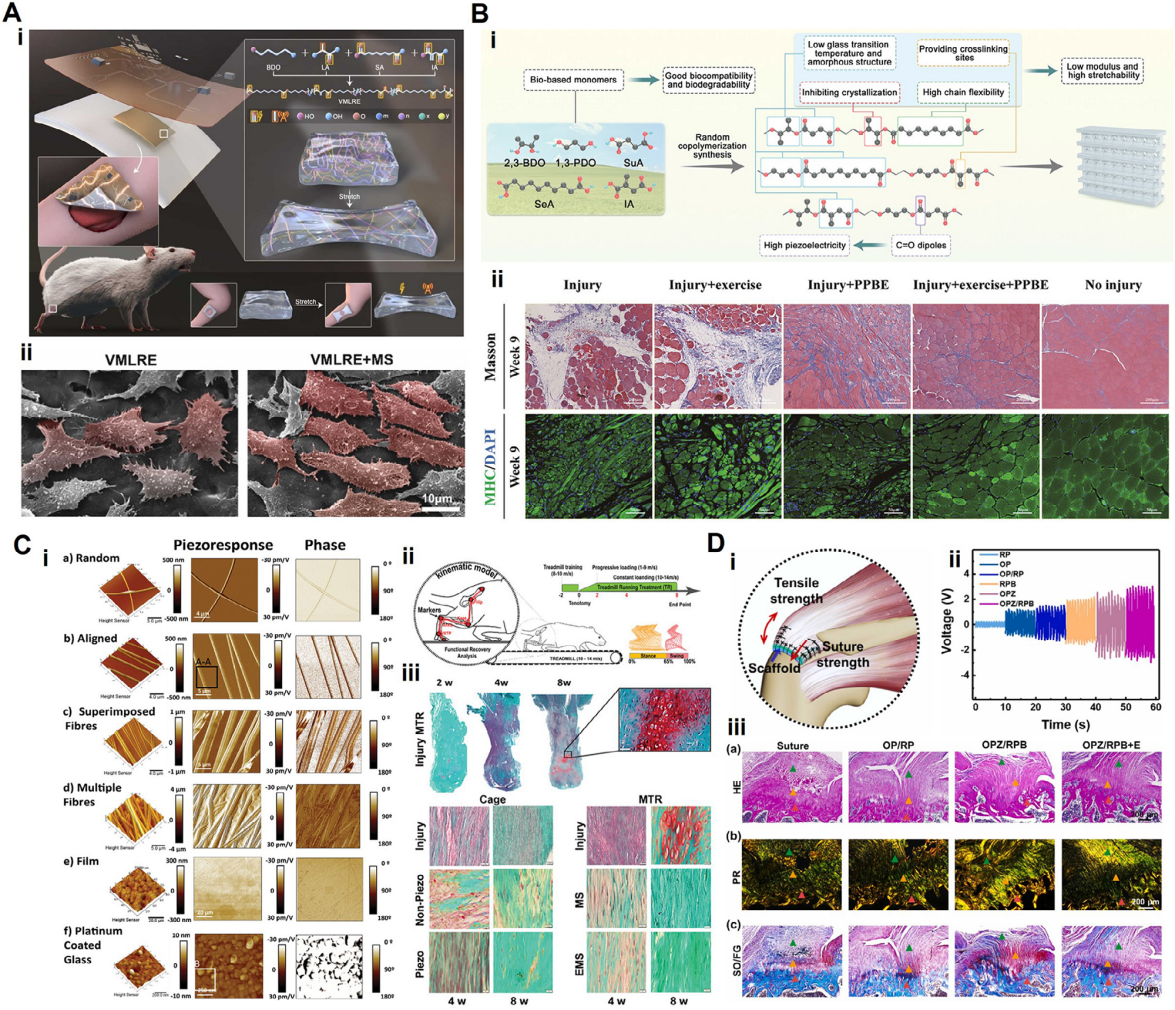
Piezoelectric scaffolds for skeletal muscle loss and tendon repair. A) A piezoelectric elastomer for the repair of volumetric muscle loss. A‐i. The schematic illustration of the composition and characteristics in treating volumetric muscle loss. A‐ii. C2C12 cells on different groups, cells are marked in pink. Reproduced with permission.^[^ ^189^ ^]^ Copyright 2023, Elsevier. B) A piezoelectric elastomer for the repair of skeletal muscle loss. B‐i. The composition of piezoelectric elastomer. B‐ii. The images of Masson and MHC staining (Scale bar: 50 µm). Reproduced with permission.^[^ ^104^ ^]^ Copyright 2024, Wiley‐VCH. C) A self‐powered piezo‐bioelectric device for tendon repair. C‐i. PFM amplitude and phase images. C‐ii. The schematic illustration of functional recovery of tendon. C‐iii. Safranin‐O stained images. Reproduced with permission.^[^ ^38^ ^]^ Copyright 2021, Wiley‐VCH. D) A Janus piezoelectric scaffolds for tendon‐to‐bone healing. D‐i. The force condition of scaffolds after implantation. D‐ii. Voltage output when subjected to periodic force (20 kPa, 1 Hz). D‐iii. Representative images of tissue recovery (red triangle: bone; orange triangular: cartilage; green triangular: tendon). Reproduced with permission.^[^ ^225^ ^]^ Copyright 2024, Elsevier.

In a bit of surprise, piezoelectric elastomers exhibit both high piezoelectricity and a robust elastic modulus, making them particularly suitable for skeletal muscle repair. For example, a piezoelectric elastomer (VMLRE) with good biocompatibility was specifically designed for the function monitoring and regeneration of volumetric muscle loss via the copolymerization of several bio‐based diacids and diols (**Figure** **10**A).^[^ ^189^ ^]^ The abundant C═O dipoles enable the elastomer with excellent piezoelectric performance for myoblast proliferation and differentiation. Another piezoelectric elastomer (PPBE), synthesized via the random copolymerization of 1,3‐propanediol (1,3‐PDO), 2,3‐butanediol, sebacic acid, succinic acid, and itaconic acid, is reported to possess both piezoelectricity and stretchability (Figure 10B).^[^ ^104^ ^]^ The C═O dipoles in the ester bonds, formed through the copolymerization of selected diacid and diol monomers, impart piezoelectricity to the material.^[^ ^390^ ^]^ Such biodegradable PPBE scaffolds have been shown to effectively treat skeletal muscle loss by promoting myogenic differentiation through the Ca^2+^ signaling pathway. Furthermore, optimizing the 2,3‐butanediol (2,3‐BDO) content in PPBE can enhance its elastic modulus, making it more suitable for muscle stretching.

### 5.4 Piezoelectric Biomaterials for Tendon Repair

Tendon injuries caused by repetitive exercises and athletic activities place an increasing economic burden on the healthcare system.^[^ ^391^ ^]^ The gold standard for treating tendon injuries is direct end‐to‐end suturing through surgical intervention; however, this method still carries a risk of failing to fully restore normal tendon function.^[^ ^392^ ^]^ During tendon repair, disorganized tissue deposition and proteoglycan accumulation can lead to chronic inflammation, which gradually progresses to tendinopathy.^[^ ^393^ ^]^ Therefore, to achieve complete tendon repair and restore normal structure and function, various approaches have been implemented to activate the endogenous signaling pathways involved in tendon regeneration.^[^ ^394^ ^]^ Moreover, recent studies have confirmed that tendon cell populations are highly mechanosensitive and contribute to tendon repair via remote mechanosensory mechanisms, including mechanosensitive ion channels.^[^ ^395^ ^]^ To a certain extent, low‐intensity exercise or in vitro shock waves have been shown to have beneficial effects on tendon repair.^[^ ^396^ ^]^ Moreover, tendon tissues are sensitive to mechanical forces and undergo 3–4% strain during daily activities, adjusting their structure and biochemical composition accordingly.^[^ ^397^ ^]^

The surface polarization of piezoelectric scaffolds has been verified to regulate stem cell behaviors, playing a crucial role in tissue repair.^[^ ^398^ ^]^ External electrical stimulation (ES) facilitates cell adhesion and collagen synthesis, and the development of self‐powered ES devices enables tissue regeneration without the need for implanted batteries.^[^ ^399^ ^]^ PVDF‐TrFE is a common piezoelectric polymer which possesses non‐centrosymmetric electrical dipoles due to the high electronegativity variation among hydrogen and fluorine atoms.^[^ ^400^ ^]^ It can be restructured into a nanofibrous scaffold in order to mimic the native electrical microenvironment and the mechanical characteristics of collagen I.^[^ ^401^ ^]^ The piezoelectric response generated by PVDF‐TrFE under mechanical loading or other external stimulation is closely related to its crystallinity, particularly the β‐phase content.^[^ ^402^ ^]^ To recover the mechanical abilities and electrochemical microenvironment of tendon tissue, piezoelectric PVDF‐TrFE fibers were fabricated with a lower annealing temperature (90 °C) and a cold drawing process to increase the β‐phase content (Figure 10C).^[^ ^38^ ^]^ Interestingly, researchers have found that fiber alignment significantly influences the piezoelectric response of PVDF‐TrFE, with higher alignment leading to greater piezoelectricity (from −16.92 to −24.61 pm V^−1^), likely because of electromechanical interactions of near fibers and stiffness gradient of materials.^[^ ^403^ ^]^

Through short‐distance electrospinning and subsequent fibronectin functionalization, the composite piezoelectric aligned PVDF‐TrFE fibers provided motion‐powered electromechanical stimulation to tendon tissue, regulating mechanosensitive signaling pathways and promoting tendon‐specific repair over non‐tenogenic tissue repair in rats with full‐thickness Achilles injuries. However, the precise mechanisms underlying tendon repair and the dominant mechanism in collagen II synthesis require further elucidation. To develop optimal piezoelectric biomaterials for tendon repair, it is important to understand how mechanical forces influence the physical abilities of tendons. Recently, Nakamichi and coworkers discovered that the PIEZO1 channel expressed in tendons could modulate physical performance. Nevertheless, how electrochemical stimulation affects the expression of ion channels like PIEZO1/2 and ultimately influences mechanosensitive signaling pathways remains unclear.^[^ ^404^ ^]^

Although piezoelectric polymers such as PVDF‐TrFE have shown effectiveness in tendon rupture repair, they are not well‐suited for in vivo transplantation involving reciprocal movements because of poor biodegradability, excellent elastic modulus, and limited elastic deformation. As previously mentioned, piezoelectric elastomers exhibit both high piezoelectricity and a low elastic modulus, meeting the requirements for tendon rupture repair.^[^ ^188^ ^]^ In this context, Ge and colleagues constructed a piezoelectric elastomer by copolymerizing 1,3‐propanediol (1,3‐PDO), succinic acid (SuA), and sebacic acid (SeA) to create a piezoelectric scaffold for regenerating rat Achilles tendons.^[^ ^190^ ^]^ The low elastic modulus (0.3 MPa) of this piezoelectric elastomer allowed the C = O dipoles to rotate, producing additional piezoelectric charge. Apart from Achilles tendons, high rotator cuff tears can result in tendon‐to‐bone injuries in the shoulder, leading to mobility dysfunction.^[^ ^405^ ^]^ Repairing tendon‐to‐bone interfaces is challenging due to the difficulty in mimicking their complex structures. Recently, researchers developed a piezoelectric scaffold with the combination of PLLA/ZnO and PLLA/BaTiO_3_ (Figure 10D).^[^ ^225^ ^]^ This bionic alignment exhibited remarkable tensile strength and suture retention, making it particularly adaptable for tendon‐to‐bone healing.

### 5.5 Piezoelectric Biomaterials for Wound Healing

Skin defects resulted from trauma, burns, and chronic diseases, including diabetes, are major medical challenges worldwide.^[^ ^406^ ^]^ Multiple treatments, including surgical debridement, antibiotic‐infused wound dressings, and tissue engineering scaffolds, have been proposed to accelerate wound healing, aiming to enhance cellular behaviors and remodel the microenvironment around the defect area.^[^ ^407^ ^]^ However, most traditional methods exhibit certain limitations, such as damage to the skin donor site, prolonged healing periods, and inadequate vascularization.^[^ ^408^ ^]^ Electrical stimulation is widely recognized as a promising tactic to accelerating wound healing.^[^ ^409^ ^]^ Piezoelectric biomaterials can produce electrical potential under mechanical or ultrasound stimulation, offering promising potential for localized electrical stimulation in wound repair.^[^ ^47^ ^]^ At this part, we have elucidated the advancements in piezoelectric biomaterials for skin repair, focusing on the healing of chronic, infected, and burn wounds.

**Figure 11:**
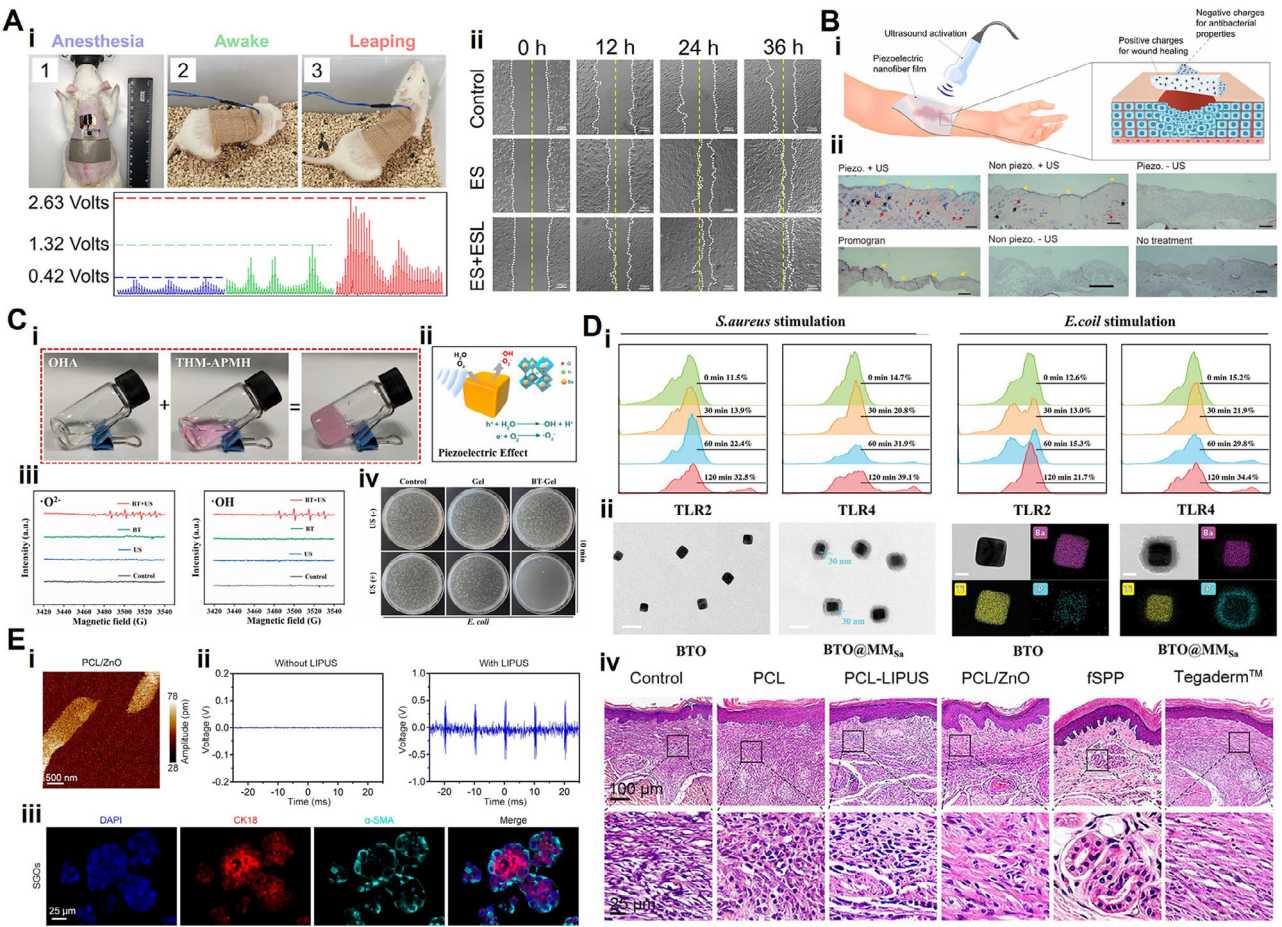
Piezoelectric scaffolds for skin regeneration. A) A wearable piezoelectric nanogenerator (PENG) for accelerated wound healing. A‐i. Open‐circuit voltage of the PENGs. A‐ii. Scratched areas (Scale bar: 100 *µm*) Reproduced with permission.^[^ ^220^ ^]^ Copyright 2023, American Chemical Society. B) A biodegradable piezoelectric scaffolds for wound healing. B‐i. The schematic illustration of PLLA scaffolds repaired a wound under ultrasound stimulation. B‐ii. H&E staining the healing of the wound. (Scale bars: 200 µm). Reproduced with permission.^[^ ^56^ ^]^ Copyright 2023, Elsevier. C) An ultrasound‐triggered scaffold for bacterial‐infected wound healing. C‐i. The digital photos. C‐ii. The generation of •OH and •O^2−^. C‐iii. Electron spin resonance spectra. C‐iv. Representative images of *E. coli* CFUs. Reproduced with permission.^[^ ^216^ ^]^ Copyright 2023, Elsevier. D) Bioinspired piezoelectric nanomaterials for infected wound repair. D‐i. Flow cytometry showing the levels of TLR2 and TLR4 expression on macrophages at different time points. D‐ii. The TEM images and elemental mapping of BaTiO_3_ nanoparticles and BaTiO_3_@MM_Sa_ nanoparticles. (Scale bars: 500 nm) Reproduced with permission.^[^ ^217^ ^]^ Copyright 2024, Wiley‐VCH. E) Flexible sono‐piezo patch for sweat gland repair. E‐i. PFM amplitude mapping of PCL/ZnO fibrous scaffolds. E‐ii. Output voltage of PCL/ZnO fibrous scaffolds. E‐iii. Immunofluorescence images of luminal marker CK18 (red) and myoepithelial marker α‐SMA (cyan) in SGOs. E‐iv. HE staining images of wound samples.^[^ ^219^ ^]^ Reproduced with permission. Copyright 2024, American Chemical Society.

The skin itself is a natural piezoelectric biomaterial, with its piezoelectricity derived from collagen fibers with a reticular structure. Recently, multiple piezoelectric skin‐wound scaffolds have been designed to generate electrical potential, aiming to suppress bacterial activity by elevating ROS levels,^[^ ^410^ ^]^ regulating the migration, proliferation, and differentiation of fibroblasts, and promoting collagen synthesis and angiogenesis (**Figure** **11**A).^[^ ^220^ ^]^ Additionally, the piezoelectric wound dressings should be capable of adsorbing exudate and should not cause patient discomfort, prolonged hospital stays, or scar formation.^[^ ^411^ ^]^ Nanofibrous piezoelectric polymers like PVDF and PLLA show promise as skin‐wound repair materials, capable of generating electrical potential to aid wound healing under mechanical stress or ultrasound. Due to its excellent biodegradability, PLLA‐based skin‐wound dressings have been reported to improve exudate drainage, inhibit bacterial infections through a negative charge, and promote re‐epithelialization through a positive charge, thereby facilitating wound healing (Figure 11B).^[^ ^56^ ^]^

Diabetic foot ulcers (DFU) occur in people with diabetes mellitus (DM) and accompanied by deep plantar lesions, as well as peripheral vascular and nerve damage.^[^ ^412^ ^]^ Common treatments for DFU, including wound debridement, skin grafts, and antimicrobial therapy, are often insufficient for all patients. Recent clinical trials have shown that electrical stimulation is a prospective strategy to enhance wound repair in DFU. However, due to patient compliance issues, the application of electrical therapy has encountered obstacles. The development of piezoelectric biomaterials is attracting significant attention because they can produce electrical stimulation subjected to mechanical force or ultrasound, providing localized electrical stimulation for DFU wound healing. For example, a composite piezoelectric hydrogel was prepared by carbonized PDA/PDA/polyacrylamide and paired with PVDF membrane to provide the protection of wound and generate electrical stimulation for faster healing.^[^ ^231^ ^]^ In addition to accelerating wound healing, wound dressings should also attract exudate and maintain a moist environment to facilitate skin repair in DFU.^[^ ^413^ ^]^ Thus, Wang et al.^[^ ^232^ ^]^ developed a self‐powered PVA/PVDF hydrogel to deliver localized electrical signal for wound repair through a sequence of freezing/thawing, solvent replacement, annealing, and swelling. The connection of PVA and PVDF enhancing β‐phase formation and crystalline structure in the piezoelectric composite hydrogel.

Bacterial colonization and proliferation around the wound area, leading to infection, is one of the primary reasons of delayed wound healing.^[^ ^414^ ^]^ Some piezoelectric biomaterials, such as ZnO nanoparticles, exhibit strong antibacterial properties with minimal need for drug assistance.^[^ ^287^ ^]^ Incorporating ZnO nanoparticles into PVDF through 3D printing technology can produce a piezoelectric wound dressing with antibacterial properties and enhanced piezoelectricity.^[^ ^141^ ^]^ An alternative antibacterial strategy, such as photodynamic therapy (PDT), could utilize the ROS to destroy bacteria.^[^ ^285^ ^]^ Researchers have reported that constructing a piezoelectric field between titanium dioxide (TiO_2_) and Au could enhance ROS production and facilitate the regeneration of infected wounds.^[^ ^230^ ^]^ Apart from PDT, another non‐invasive strategy to generate toxic ROS for bacterial killing is sonodynamic therapy (SDT), which offers enhanced soft tissue penetration without additional skin phototoxicity.^[^ ^415^ ^]^ Thus, combining ultrasound (US) with piezoelectric biomaterials, such as BaTiO_3_, shows significant potential for infectious wound healing (Figure 11C).^[^ ^216^ ^]^

Moreover, incorporating antibiotic molecules, such as, could also confer antimicrobial and hydrophilic characteristics to the piezoelectric wound dressing.^[^ ^41^ ^]^ With the development of precision therapy, researchers have indicated that by coating piezoelectric nanomaterials with macrophage membranes pre‐activated by *Staphylococcus aureus*, these materials could specifically recognize bacteria and rapidly generate ROS under US irradiation to suppress bacterial metabolism, thereby enhancing wound healing (Figure 11D).^[^ ^217^ ^]^ Besides the need to quickly repair the wound and eliminate bacterial infection, the repair of sweat glands in burn injuries remains challenging and poorly understood. Recently, Pi and coworkers reported that electrical stimulation generated by a sono‐piezoelectric patch could create a regenerative microenvironment to facilitate sweat gland regeneration, offering a promising approach for the clinical repair of burn injuries (Figure 11E).^[^ ^219^ ^]^

### 5.6 Piezoelectric Biomaterials for Nerve Regeneration

The impairments to the central and peripheral nerves resulting from traffic‐related trauma or injuries pose an increasing burden on the field of neural regenerative medicine.^[^ ^416^ ^]^ Electrical stimulation has demonstrated a significant therapeutic effect on nerve repair.^[^ ^417^ ^]^ Traditional electrical stimulation therapy is invasive and can cause inflammation or infection at a high risk. Therefore, the development of tissue engineering scaffolds capable of generating electrical stimulation for nerve repair is highly urgent and holds significant application potential. Among the various types of nerve injuries, spinal cord injury is one of the most severe forms of nerve damage due to the disruption of sensory and motor functions.^[^ ^418^ ^]^ Surgical decompression is utilized in the management of spinal cord injury to restore blood supply and reduce secondary injury.^[^ ^419^ ^]^ However, this approach primarily alleviates symptoms and yields limited nerve repair outcomes.^[^ ^420^ ^]^

**Figure 12:**
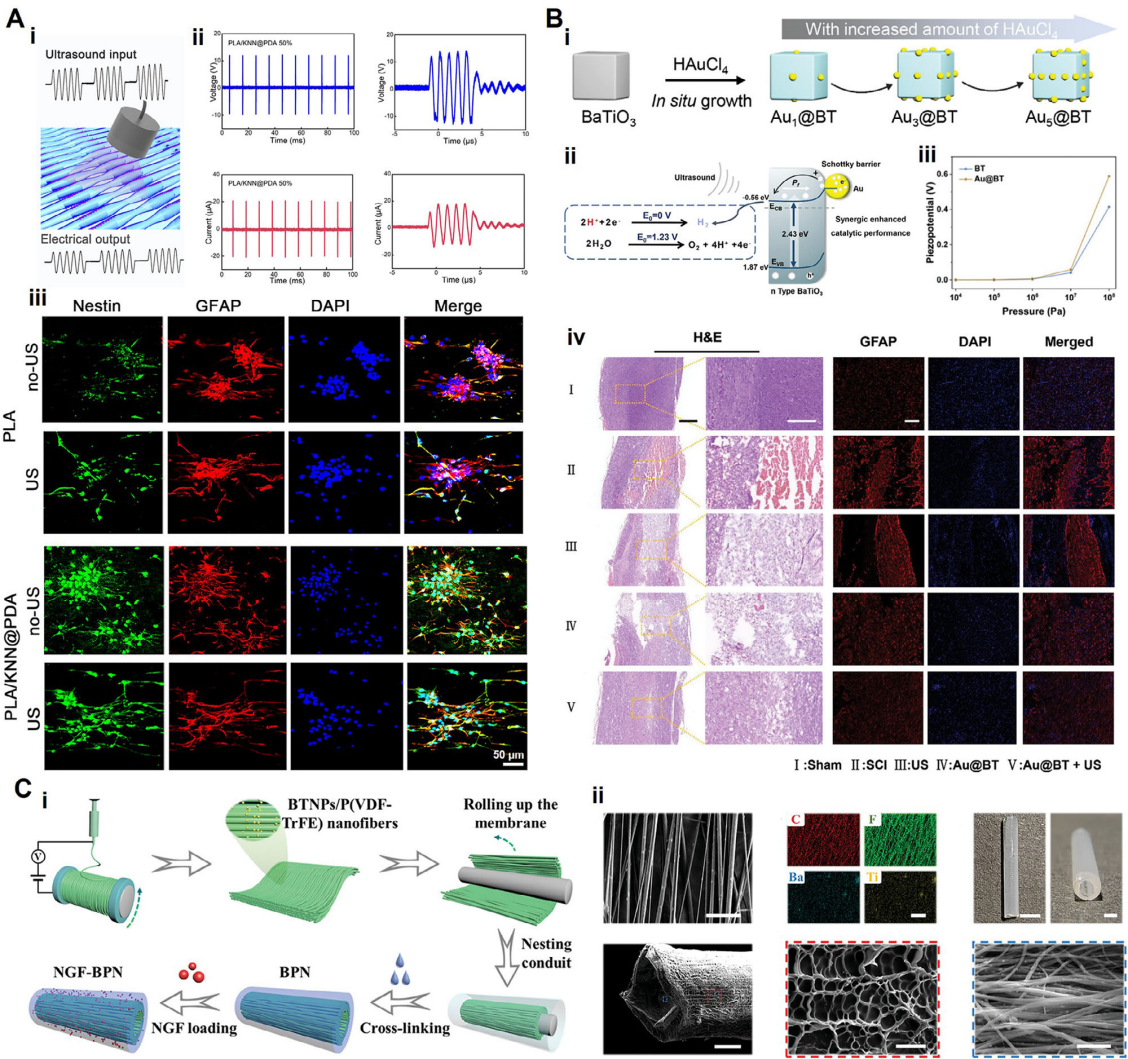
Piezoelectric biomaterials for the spinal cord injury repair and peripheral nerve regeneration. A) A 3D piezoelectric scaffold for promoted spinal cord injury repair. A‐i. Schematic illustration of the electrical output. A‐ii. The open‐circuit voltage and short‐circuit current of PLA/KNN@PDA nanofibers. A‐iii. Immunofluorescent staining of Nestin and glial fibrillary acidic protein (GFAP). (Scale bars: 50 µm). Reproduced with permission.^[^ ^135^ ^]^ Copyright 2022, American Chemical Society. B) Piezoelectric‐catalytic nanoparticles for acute spinal cord injury repair. B‐i. The synthetic process of Au_x_@BaTiO_3_ nanoparticles. B‐ii. Scheme of band tilting and piezoelectric catalytic reaction of Au@BaTiO_3_. B‐iii. Piezoelectric potentials. B‐iv. H&E staining images of spinal cord tissue. (Scale bar: 500 µm.) The yellow boxes indicate enlarged images of the injured site. (Scale bar: 200 µm.) Immunofluorescence images of GFAP. Reproduced with permission.^[^ ^229^ ^]^ Copyright 224, Wiley‐VCH. C) Ultrasound‐responsive aligned piezoelectric nanofibers derived hydrogel conduits for peripheral nerve. C‐i. Schematic illustration of the fabrication process. C‐ii. SEM images of the piezoelectric nanofibers derived hydrogel NGC. Scale bars are 5 µm in (top left corner), 15 µm in (EDS mapping), 3 mm and 1 mm in (top right corner), 500 µm in (bottom left corner), 50 µm in (the inside of the red box), and 10 µm in (bottom right corner), respectively. Reproduced with permission.^[^ ^227^ ^]^ Copyright 2024, Wiley‐VCH.

Acute spinal cord injury is often accompanied by an excessive inflammatory response, with glial scars more readily forming due to the activity of astrocytes and macrophages activated by lipid peroxidation, acid‐base disturbances, ROS accumulation, and the secretion of inflammatory factors, which hinder spinal cord recovery.^[^ ^282a^ ^]^ Therefore, alleviating inflammation and eliminating excess ROS are essential strategies to promote spinal cord regeneration. In 2022, Chen and coworkers designed a piezoelectric scaffold for spinal cord injury repair (**Figure** **12**A).^[^ ^135^ ^]^ In vitro and in vivo results demonstrated that electrical stimulation generated by piezoelectric PLA/KNN/PDA nanofibers under ultrasound stimulation promotes neural differentiation. Although piezoelectric scaffolds can generate electrical stimulation to facilitate spinal cord repair, the rate of nerve repair and regeneration remains inhibited by inflammation and excessive ROS. Inspired by this, You and colleagues developed a gold nanoparticle‐modified piezoelectric BaTiO_3_, capable of continuously producing H_2_ in aqueous solution under ultrasound irradiation (Figure 12B).^[^ ^229^ ^]^ The H_2_ exhibits potent therapeutic effects in clearing excess ROS in cells and alleviating the pathological inflammatory microenvironment in acute spinal cord impairments.

As another form of central nervous system injury, traumatic brain injury (TBI) represents the leading cause of disability and mortality in young populations.^[^ ^421^ ^]^ Following injury, damaged neurons are cleared by glial cells, which proliferate to form glial scars, resulting in persistent neurological deficits.^[^ ^422^ ^]^ Electrical stimulation has been demonstrated to promote neuronal differentiation of neural stem cells (NSCs) and facilitate the reconstruction of neural circuits.^[^ ^353a^ ^]^ However, conventional piezoelectric nanoparticles are prone to endocytosis by NSCs, where ultrasound‐induced ROS generation within lysosomes triggers cell death. In a recent study, Wang et al.^[^ ^423^ ^]^ engineered a BaTiO_3_/rGO hybrid piezoelectric nanopatch for NSC‐based therapy in TBI. This nanopatch, when activated by US, generated sustained piezoelectric potentials on NSC membranes, thereby activating VGCCs and downstream signaling pathways to accelerate neuronal differentiation and maturation of NSCs. In a rat TBI model, transplantation of NSCs integrated with the nanopatch combined with US significantly restored brain tissue integrity and neurological functions within 28 days.

Besides central nervous system injury, peripheral nerve injury (PNI) is also a common neurological disorder, which can be categorized as peripheral nerve necrosis, defects, or ruptures resulting from external force injuries, among other causes.^[^ ^424^ ^]^ Currently, the gold standard for remedying PNI remains autologous transplantation, which is curbed by the availability of donor tissue, mismatched graft size, and secondary injury to the donor site. Therefore, artificial nerve guidance conduits (NGCs) have vast potential in treating PNI.^[^ ^425^ ^]^ In recent years, researchers have reported that electrical stimulation produced by piezoelectric biomaterials under mechanical stress or ultrasound could induce the Ca^2+^ influx and activate neurite growth and differentiation.^[^ ^426^ ^]^

From a microscopic perspective, the peripheral nerve comprises neurons, myelin sheaths, and connective tissues.^[^ ^427^ ^]^ Piezoelectric materials are utilized to repair PNI in the form of biomimetic grafts and NGCs, which are designed to restore the microenvironment of the defect area and modulate the proliferation and maturation of Schwann cells.^[^ ^428^ ^]^ For instance, a composite piezoelectric nanogenerator, PHBV/PLLA/KNN, was developed to produce localized electrical stimulation for sciatic nerve injury repair, monitored by ultrasound.^[^ ^221^ ^]^ Schwann cell cords formation is important to facilitate the repair of axon.^[^ ^429^ ^]^ Mao et al.^[^ ^137b^ ^]^ have reported that ZnO, a piezoelectric bioceramic incorporated into the biodegradable polymer PCL, could deliver effective endogenous electrical stimulation to accelerate peripheral nerve repair through the RAS/MAPK pathway, which is activated by the expression of growth factor receptor‐bound protein 2 (GRB2). As for large nerve defects, it remains challenging to achieve complete repair solely by implanting an artificial piezoelectric NGC. To solve this obstacle, a multi‐channel piezoelectric NGC has been proposed to facilitate PNI repair via the synergistic outcome of piezoelectric PVDF and conductive SF/PEDOT cryogel.^[^ ^54^ ^]^ Moreover, electrical stimulation has been reported to enhance axon extension and myelination, and attenuate muscle atrophy.^[^ ^430^ ^]^

The structure and function of nerves are compatible; anatomically, peripheral nerves are primarily composed of myelinated parallel axon tracts, indicating that constructing a bionic NGC scaffold could guide neuronal growth.^[^ ^431^ ^]^ Inspired by this, Xiong and colleagues developed a PPy/PDA/PLLA fibrous scaffold to repair a 10 mm sciatic nerve defect in vivo.^[^ ^174^ ^]^ It has been corroborated that the aligned structure of the fibrous scaffold can lead to the aligned extension and alignment of dorsal root ganglion (DRG) neurites. The fiber topography of piezoelectric fibrous scaffolds strongly influences the migration, alignment, proliferation, and differentiation of neuronal cells.^[^ ^432^ ^]^ Compared to custom‐patterned scaffolds, research has shown that piezoelectric fibrous scaffolds with a topological gradient structure can significantly modulate neuronal cell behaviors by influencing the cytoskeleton.^[^ ^226^ ^]^ Moreover, to accelerate the healing speed of PNI, nerve growth factor (NGF) was loaded into a thermoresponsive hydrogel coating on the outer layer of oriented piezoelectric BaTiO_3_/P(VDF‐TrFE) nanofibers (Figure 12C).^[^ ^227^ ^]^ Interestingly, researchers have found that ultrasound‐induced local temperature increases can accelerate the shrinkage of the outer hydrogel containing NGF, ultimately enhancing neuronal outgrowth. Therefore, the development of biomimetic piezoelectric NGCs could modulate neural cell fate through the synergistic effects of topographical structuring and electrical stimulation.

### 5.7 Piezoelectric Biomaterials for Teeth and Periodontal Tissues Regeneration

Teeth and periodontal tissues are important elements of oral health. Dentin is the primary structural component of the tooth's hard tissue, covered by enamel on the exterior and connected internally to the pulp cavity.^[^ ^433^ ^]^ Periodontal tissue mainly comprises the gingiva, periodontal ligament, alveolar bone, and cementum. The piezoelectric effect in teeth is primarily exhibited in dentin and cementum, which contain abundant collagen fibers. These tiny electrical signals are crucial for biomechanical sensing, restoration, and maintaining tooth health. In periodontal tissues, both the cementum and periodontal ligament display piezoelectric properties. These properties in periodontal tissues are connected to the biomechanical function of teeth, tissue repair, and regeneration processes. Piezoelectricity introduces new avenues for research and development in dental restoration materials. For instance, biomaterials with piezoelectric effects can support dental injury repair and strengthen bonding between restoration materials and natural teeth. Additionally, these scaffolds can produce electrical fields under occlusal forces, which may facilitate periodontal tissue health.

**Figure 13:**
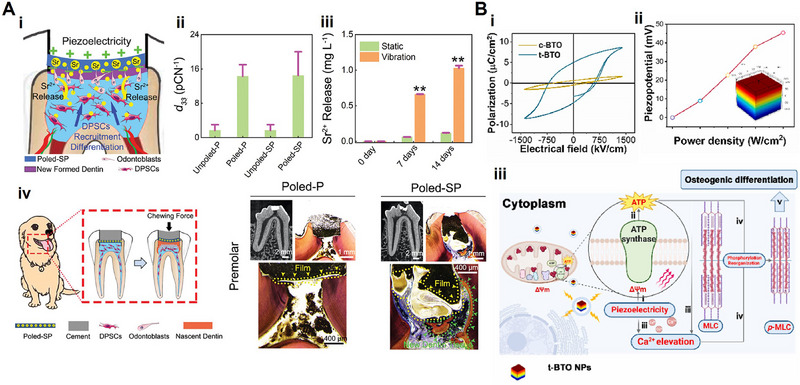
Piezoelectric scaffolds for tooth and periodontal tissue regeneration. A) A strontium‐contained piezoelectric film for dentin regeneration. A‐i. The progress of dentin‐pulp complex repair. A‐ii. Piezoelectric coefficient (d_33_) of different groups. A‐iii. Sr^2+^ concentration. A‐iv. Schematic illustration and X‐ray images and Van Gieson stain. Reproduced with permission.^[^ ^57^ ^]^ Copyright 2024, Wiley‐VCH. B) A piezoelectric hydrogel for the treatment of periodontitis. B‐i. The polarization‐electric field hysteresis curves. B‐ii. COMSOL simulation of the piezopotential. Inset indicates the piezopotential distribution under 2 W cm^−2^ US stimulation. B‐iii. Mechanism schematic illustration. Reproduced with permission.^[^ ^39^ ^]^ Copyright 2024, Elsevier.

For example, in efforts to repair dental tissue, piezoelectric BaTiO_3_ nanoparticles have been incorporated into dental‐filling resin to enhance remineralization.^[^ ^434^ ^]^ Nevertheless, resin may not be an ideal material for tooth regeneration, as its acidic monomer could potentially cause severe pulp damage.^[^ ^435^ ^]^ Recently, a novel piezoelectric biofilm has been developed to support dentin tissue repair during daily physiological activities like talking and chewing (**Figure** **13**A).^[^ ^57^ ^]^ The piezoelectric strontium‐containing P(VDF‐TrFE) possessed a piezoelectric d_33_ coefficient of 14 pC N^−1^, which could provide an electrical microenvironment to recruit dental pulp stem cells (DPSCs) and induced them into odontoblasts. As for the repair of periodontal tissues, bacterially caused inflammation has caused a significant obstacle, preventing bone formation. In 2023, Roldan and coworkers designed a piezoelectric hydrogel with piezoelectric BaTiO_3_ modified in gelatin methacryloyl (GelMA) (PiezoGEL) for the remedy of periodontal disease, indicating that electrical stimulation generated by PiezoGEL under mechanical loading could inhibit bacteria activity and promote osteogenesis.^[^ ^218^ ^]^ In the development of periodontitis, the periodontal ligament stem cells (PDLSCs) suffer from severe mitochondrial dysfunction with inadequate ATP production.^[^ ^436^ ^]^ A recent work have reported that electrical stimulation generation by a piezoelectric hydrogel incorporated by BaTiO_3_ nanoparticles under mechanical activation could improve the mitochondrial function of PDLSCs and induce osteogenic differentiation (Figure 13B).^[^ ^39^ ^]^ Moreover, the piezoelectrical stimulation and anti‐inflammatory property of hydrogel led macrophages switched into anti‐inflammatory subtypes for osteogenesis.

### 5.8 Piezoelectric Biomaterials for Myocardial Regeneration

Myocardial tissue engineering aims to reconstruct the regenerative microenvironment of myocardial tissue through the use of polymer scaffolds, thereby promoting the functional maturation of seeded cardiomyocytes.^[^ ^437^ ^]^ This approach facilitates the formation of a biomimetic, tissue‐engineered myocardium, with significant potential for repairing or replacing damaged heart tissue. Numerous researches have shown that some external electrical stimulation can mimic the electrical conduction characteristics of natural myocardium, facilitate the rapid propagation of electrical signals in myocardial cells, and support cell coupling, synchronized contraction, and the formation of ultrastructural organization.^[^ ^438^ ^]^

**Figure 14:**
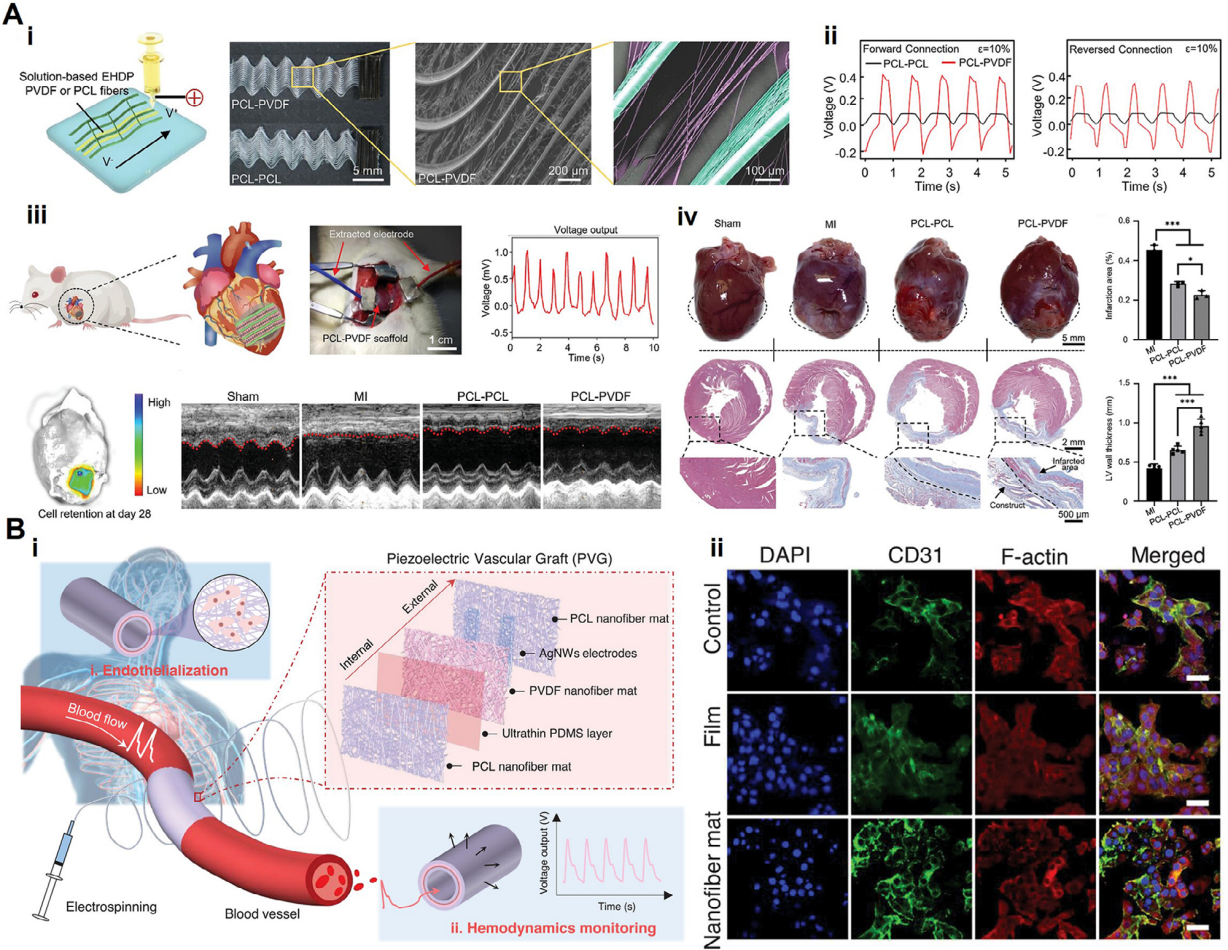
Piezoelectric scaffolds for myocardial regeneration and served as artificial vascular graft. A) Synergistic mechanical/piezoelectric stimulation to enhance cardiomyocytes‐specific function. A‐i. Schematic diagram of solution‐based EHD printing of PVDF or PCL fiber among the serpentine melt‐based EHD printed PCL microarchitectures. Representative optical images and SEM images. Green represents melt‐based PCL fibers, and purple represents solution‐based PVDF fibers. A‐ii. The output voltage of encapsulated PCL‐PCL and PCL‐PVDF scaffolds. A‐iii. Schematic of PCL‐PVDF scaffold placed on the infarcted rat heart. Output voltage of the PCL‐PVDF scaffold. High CM retention in MI area after 28 days of post‐transplantation using PCL‐PVDF cardiac constructs. Representative M‐mode parasternal long‐axis view of LV echocardiographic images. A‐iv. Overall and Masson's trichrome staining from all groups after 28 days of post‐transplantation. Reproduced with permission.^[^ ^233^ ^]^ Copyright 2024, Wiley‐VCH. B) A piezoelectric vascular graft for hemodynamic monitoring. B‐i. Overall view for PVG, illustrating structure design and advantages. The proposed PVG is based on exquisite electrospinning nanofibers, with endothelialization facilitation and hemodynamics monitoring objectives. B‐ii. Fluorescent images presenting the HUVECs morphologies and phenotype on different substrates, including control (dish), PCL film, and PCL nanofiber mat. (Scale bar: 50 µm.) Reproduced with permission.^[^ ^234^ ^]^ Copyright 2024, Wiley‐VCH.

Piezoelectric scaffolds, such as PVDF and PHBV, can produce in situ electrical stimulation when they are deformed, offering a novel strategy for electrical stimulation in tissue regeneration.^[^ ^439^ ^]^ Furthermore, using 3D printing and other methods, piezoelectric materials that mimic the myocardial ECM can be prepared with various biomimetic physical characteristics, such as topology, mechanical properties, and piezoelectricity, enhancing their suitability for myocardial regeneration.^[^ ^440^ ^]^ For instance, Han et al.^[^ ^233^ ^]^ applied a multimaterial 3D printing technology to manufacture piezoelectric materials for cardiac tissue repair (**Figure** **14**A). The scaffolds were composed of PCL, PVDF, and magnetic PCL/Fe_3_O_4_ microfibers, where PCL provided mechanical support, the PCL/Fe_3_O_4_ component enabled external magnetic control, and the deformation of PVDF generated an in situ electric field for myocardial regeneration. Such engineered cardiac piezoelectric scaffold significantly restored ventricular structure and function, reduced fibrosis and inflammation, and promoted angiogenesis. However, the mechanisms by which cardiomyocytes respond to piezoelectric materials and electrical stimulation remain unclear, and the interactions between piezoelectric‐induced electrical stimulation and the ion‐based electricity of cells and tissues require further investigation.

### 5.9 Piezoelectric Biomaterials for Artificial Vascular Grafting

Artificial vascular grafts are medical devices specifically designed to replace or repair damaged vascular structures and are frequently employed in cardiovascular surgeries, revascularization procedures, or vascular grafting necessitated by trauma, tumor resection, and other medical conditions.^[^ ^441^ ^]^ Blood vessels exhibit inherent piezoelectricity due to the specific molecular structures within their walls, particularly those related to collagen arrangement and cellular mechanical properties.^[^ ^69^ ^]^ Recently, researchers have concentrated on the piezoelectric properties of blood vessels in physiological functions and pathological processes, with particular attention to cardiovascular disease, vascular regeneration, and repair.^[^ ^442^ ^]^

Notably, researchers have reported that artificial vascular grafts prepared by piezoelectric polymers could sense physical deformations such as extrusion, compression, and bending, and convert them into electrical signals to accelerate vascular endothelialization (Figure 14B).^[^ ^234^ ^]^ To test this hypothesis, electrospinning technology has been adapted to fabricate a piezoelectric artificial vascular graft that mimics the multilayer composition of a blood vessel. The inner layer of the composite graft consists of a porous PCL nanofiber mat permeated with an ultrathin PDMS layer that promotes cell proliferation and differentiation, thereby accelerating vascular endothelialization. The middle layer comprises a PVDF nanofiber mat with patterned silver nanowire (AgNW) electrodes specifically designed to generate in situ electrical potential under deformation. The outer layer, a PCL nanofiber mat, provides the scaffold with additional mechanical support. This piezoelectric artificial vascular graft promotes vascular endothelialization, monitors hemodynamics, exhibits excellent biocompatibility, supports the advancement of intelligent artificial blood vessels, and enables real‐time hemodynamic monitoring and intelligent vascular health management.

### 5.10 Piezoelectric Biomaterials for Cornea Repair

Similar to native bone, the cornea's piezoelectric properties stem from its distinctive collagen fiber arrangement. When subjected to external forces—such as pressure or stretching—the collagen fibers in the cornea undergo slight deformation, inducing a charge shift and microcurrent generation. However, the dense arrangement and transparency of corneal fibers render this effect uniquely subtle. Due to the cornea's significantly lower Young's modulus compared to bone, the piezoelectric coefficient of the cornea is ≈20 000 times greater.^[^ ^443^ ^]^ Consequently, the cornea exhibits more pronounced strain under equivalent stress, resulting in a higher piezoelectric coefficient.^[^ ^444^ ^]^ Piezoelectric stimulation has been observed to initiate certain signaling pathways in corneal cells, including calcium channels and growth factor receptors. The electric current generated within the cornea can alter the potential difference across the cell membrane, thereby enhancing signal transduction, which in turn regulates cell growth and differentiation. Thus, the piezoelectric properties of the cornea present significant potential for applications in corneal regeneration and repair.

However, existing device limitations and the complexity of implementation have impeded the broader application of endogenous piezoelectricity in corneal repair. Notably, researchers have proposed piezoelectric contact lens designed to switch the mechanical motion of blinking into unidirectional electric signals, enabling the repair of moderate corneal injuries.^[^ ^235^ ^]^ This flexible piezoelectric contact lens comprises a piezoelectric electret film (d_33_∼145 pC N^−1^) featuring a fractal constitution, a miniaturized rectifier, and a pair of transparent, stretchable stimulation electrodes. This study demonstrates that piezoelectric scaffolds can bridge the electrical field and physical movements, employing artificial endogenous enhancement signals resulted from spontaneous human movements to promote corneal regeneration, thereby offering a novel approach to corneal injury repair.

In this section, we systematically review piezoelectric biomaterials for regenerating diverse tissues, encompassing hard and soft tissue repair as well as composite tissue regeneration. Specific regenerative scenarios necessitate tailored design and selection of piezoelectric biomaterials with tissue‐specific adaptability. Primarily, piezoelectric films (e.g., PLLA or PVDF) are suitable for defects subjected to relatively low mechanical loads, such as cranial defects and cutaneous wound healing. Conversely, for large‐scale bone defects or spinal cord injuries, 3D‐printed piezoelectric composite implants or surface‐functionalized orthopedic implants incorporating piezoelectric nanoparticles are required to augment mechanical integrity and piezoelectric output. Injectable piezoelectric hydrogels demonstrate unique advantages for cartilage repair due to the clinically requirement for minimally invasive implantation. These hydrogels can be engineered by incorporating piezoelectric nanoparticles, nanorod‐structured piezoelectric polymers, and biomimetic extracellular matrices. This integrated design enhances the piezoelectric performance of the hydrogel system while simultaneously promoting chondrocyte differentiation via electrical stimulation.

As for the repair of skeletal muscle and Achilles tendon subjected to cyclic mechanical strain, highly stretchable piezoelectric elastomers demonstrate exceptional compatibility. These materials maintain stable piezoelectric output while sustaining long‐term reciprocating motion, thereby providing continuous electromechanical stimulation critical for tissue regeneration. Clinical pathologies such as diabetic bone/skin defects and acute brain injuries are frequently complicated by bacterial infections, necessitating integrated anti‐infective strategies during tissue repair. Piezoelectric nanomaterials with intrinsic bactericidal properties (e.g., ZnO) and some piezoelectric antimicrobial peptides offer a dual‐functional solution: Under external ultrasound stimulation, these piezoelectric biomaterials can generate electrical stimulation to promote tissue regeneration simultaneously producing ROS that disrupt bacterial biofilms. More importantly, for constructing highly specialized tissues (e.g., myocardium, peripheral nerves, cornea), 3D printing and other microfabrication technologies enable the fabrication of biomimetic piezoelectric scaffolds. These engineered constructs reconstitute tissue‐specific electromechanical microenvironments to promote functional restoration of damaged regions.

## 6 Conclusion and Perspectives

Piezoelectric biomaterials, including piezoelectric bioceramics, polymers, natural materials, and composites, hold significant potential for regenerating various hard and soft tissues because of their unique property to produce electrical stimulation under mechanical force or other external stimuli. The electrical stimulation produced from piezoelectric biomaterials can regulate cell behavior, enhance ECM formation, promote angiogenesis, modulate immune responses, and exhibit antibacterial effects, making it a highly promising approach for regenerative medicine. It is crucial to mention that piezoelectric biomaterials have a long history of regenerative application beginning in ≈1957. The translational potential of piezoelectric biomaterials is immense, as ongoing research has significantly broadened their application scenarios by integrating multiple therapeutic constituents to achieve synergistic outcomes. This review presents an in‐depth understanding of recent advances in endogenous piezoelectricity and piezoelectric biomaterials for tissue engineering, with a particular emphasis on strategies for designing piezoelectric scaffolds tailored to the specific piezoelectric properties and structural requirements of various tissue defects. These insights aim to facilitate the development of novel piezoelectric scaffolds and devices for regenerative medicine. Nonetheless, several challenges remain that hinder the clinical translation of piezoelectric biomaterials and their associated devices in the medical field.

Regarding endogenous piezoelectricity, extensive research has been conducted to elucidate the molecular structures and underlying mechanisms responsible for piezoelectric effects in key biological macromolecules, particularly collagen. While other biomacromolecules such as HAp and elastin exhibit comparatively weaker piezoelectric properties, their distinct molecular architectures and associated piezoelectric phenomena nevertheless offer valuable insights for the development of novel piezoelectric biomaterials. This underscores the need for further systematic investigation into these endogenous piezoelectric components. Moreover, it should be noted that current understanding of tissue‐level endogenous piezoelectricity primarily derives from studies on isolated tissue specimens. However, such experimental conditions may not accurately replicate the physiological piezoelectric responses observed in living tissues. Consequently, significant challenges remain in precisely characterizing the piezoelectric properties of in vivo tissues and determining their specific biological origins. These knowledge gaps highlight critical directions for future research in this field.

From the perspective of precise mechanisms and intracellular signaling pathways participated in the interaction between piezoelectric biomaterials and tissue defects, it is essential to elucidate the sources of electrical stimulation. These sources may stem from the endogenous piezoelectricity of natural tissues, the deformation of piezoelectric biomaterials under external stimuli, or their spontaneous formation. Currently, research efforts have predominantly concentrated on optimizing piezoelectric scaffolds and enhancing the current output of piezoelectric biomaterials, often overlooking how variations in output currents influence cellular behaviors, ECM formation, immune cell interactions, and microscopic changes in the behavior of organelles. Furthermore, despite the integration of functional molecules and electrical stimulation, even minor fluctuations in current output can significantly alter the processes and outcomes of therapeutic interventions. Moving forward, it is imperative to determine the specific output forms and optimal current intensities of piezoelectric materials that can facilitate cell differentiation and tissue regeneration, tailored to the requirements of various cell types and tissues. Additionally, the mechanical characteristics and biodegradability of these materials need to be further refined to match specific application scenarios, thereby minimizing the need for secondary surgical interventions post‐implantation.

In terms of clinical translation, the convenience of piezoelectric biomaterials and the compliance of patients have limited the promotion and application of piezoelectric implants. To solve this problem, researchers have utilized rehabilitation training after clinical surgery and set the piezoelectric implants in the organized areas of movements, such as knee joints, to provide electrical stimulation for the repair of tissue defects under mechanical stress. Most piezoelectric biomaterials require the application of appropriate external stimuli, which may influence the patient's compliance and limit their application to a certain extent. Meanwhile, the source of piezoelectric biomaterials will affect their application scenarios, for instance, natural piezoelectric materials such as collagen, silk fibroin and cellulose possess weak piezoelectricity but excellent biocompatibility, while the synthetic piezoelectric bioceramics or polymers have more satisfied piezoelectric properties and their biosafety and in vivo degradation product toxicity is an issue that deserves the attention and resolution of researchers.

Moreover, insufficient long‐term electroactivity stability represents a critical bottleneck hindering the clinical translation of piezoelectric biomaterials. Piezoelectric polymers like PLLA and PVDF exhibit significant attenuation in piezoelectric coefficient under prolonged cyclic mechanical loading, while inherent brittleness renders piezoelectric bioceramics prone to fracture within physical movements of tissues. Consequently, advancing novel piezoelectric composite designs is imperative. Self‐powered piezoelectric biomaterials capable of sustained electrical output without external stimuli, alongside β‐phase stabilization of piezoelectric polymers, hold substantial translational potential.

Last but not least, tissue‐specific regenerative scenarios impose distinct requirements on the biodegradation kinetics of piezoelectric biomaterials. For instance, peripheral nerve repair demands shorter regeneration periods, where PVDF‐based NGCs may cause nerve compression due to material retention. Conversely, large‐segment bone defects necessitate extended repair timelines, while PLLA scaffolds often degrade prematurely. It will result in mechanical support failure. Similarly, rapidly degrading collagen‐based piezoelectric hydrogels for cartilage repair often lead to compromised outcomes, as incomplete regeneration occurs due to the early material resorption. Thus, designing personalized piezoelectric composites with multistage degradation system is critical to synchronize degradation with tissue‐specific regenerative timelines.

In recent years, optimizing the morphology and structure of piezoelectric scaffolds to optimize their piezoelectric properties, and improving the targeting efficiency of piezoelectric nanomaterials to damaged tissues through methods such as cell membrane coating, have become significant research hotspots. Studies have demonstrated that coating piezoelectric nanoparticles with macrophage membranes pre‐activated by bacteria can enhance the expression of toll‐like receptors on cell membranes, thereby improving the delivery efficiency of the nanosystem. However, additional strategies to enhance the targeting efficiency of piezoelectric nanomaterials require further exploration and validation in disease models. Moreover, with the advancement of 3D printing technology, individualized, layer‐by‐layer, multilateral, and customizable piezoelectric scaffolds can be fabricated for applications in wearable and sensitive electronics, enabling the monitoring of body functions and promoting tissue repair. The future integration of artificial intelligence will further assist researchers in predicting the output voltage of piezoelectric biomaterials after implantation in the body, thereby advancing their use in clinical applications.

In conclusion, piezoelectric biomaterials offer immense potential in regenerative medicine because of their unique piezoelectric characteristics and the substantial scope for further development. This work provides a comprehensive overview of recent advancements, challenges, and future perspectives regarding the application of piezoelectric scaffolds in tissue regeneration. We believe that this review will enhance the understanding of existing piezoelectric biomaterials and elucidate the specific mechanisms by which their electrical stimulation promotes tissue regeneration. Additionally, it aims to assist researchers in optimizing scaffold designs for the regeneration of both hard and soft tissues.

## Conflict of Interest

The authors declare no conflict of interest.

## Author Contributions

X. D., Y. Z., and J. C., contributed equally to this work. X.D. contributed to writing – review and editing, writing – original draft, and investigation; Y.Z. to writing – review and editing and data curation; J.C. to writing – review and editing, data curation, and investigation; L.W. to formal analysis and investigation; H.Z. to formal analysis and investigation; and X.W., K.L., and C.Y. to writing – review and editing, supervision, project administration, methodology, funding acquisition, and conceptualization.
